# Deficiency of UBE3D in mice leads to severe embryonic abnormalities and disrupts the mRNA of Homeobox genes via CPSF3

**DOI:** 10.1038/s41420-025-02387-y

**Published:** 2025-03-12

**Authors:** Yiwei Mi, Lu Yan, Yu Wu, Yufang Zheng

**Affiliations:** 1 Institute of Developmental Biology & Molecular Medicine, Dept. of Cellular & Developmental Biology, State Key Laboratory of Genetic Engineering at School of Life Sciences, Fudan University, Shanghai, 200433 China; 2 Obstetrics and Gynecology Hospital, The institute of Obstetrics and Gynecology, Fudan University, Shanghai, 200011 China

**Keywords:** Neurulation, Ubiquitylated proteins

## Abstract

Neurulation is a crucial event during vertebrate early embryogenesis, and abnormalities in this process can result in embryonic lethality or congenital disorders, such as neural tube defects. Through our previous phenotypic-driven screening in mice, we have identified UBE3D as a key factor for the neurulation process. By generating *Ube3d* knockout mice using CRISPR/Cas9 technology, we observed that homozygous mice exhibited severe growth retardation and malformation, ultimately dying between E10.5 to E11.5. In contrast to their wild-type and heterozygote littermates, homozygous embryos displayed small heads and unturned caudal neural tubes at E9.5. Our in situ hybridization and immunofluorescence experiments revealed high expression of UBE3D in the forebrain, neural tube, and heart at E9.5–10.5. Furthermore, RNA-seq analysis of the E10.5 embryos demonstrated that deficiency in UBE3D resulted in the downregulation of multiple Homeobox genes, including those specifically expressed in the forebrain and lumbosacral regions. We also discovered that UBE3D interacts with CPSF3, which is an endonuclease essential for the pre-mRNA 3’ end process. UBE3D could de-ubiquitinate CPSF3, and a deficiency of UBE3D leads to reduced levels of CPSF3 in both mouse and human cells. Overexpression of dominant negative mutants of CPSF3 was found to partially reduce mRNA levels of several Homeobox genes. In summary, our findings highlight that UBE3D is critical for early embryonic development in mice.

## Introduction

The neurulation stage is a critical period in mouse embryonic development, during which both the formation of the neural tube and organogenesis begins. In mouse, the period from E8.5 to E11.5 is particular crucial for neurulation [1]. This intricate process is tightly regulated by numerous genes, including Homeobox genes that control the anterior-posterior (A-P) body plan. As the A-P axis elongates, the predetermined neural ectoderm begins to pattern along this axis to form the neural tube, with its anterior portion giving rise to the brain.

Homeobox genes, containing a highly conserved Homeobox domain, function as transcription factors to regulate cell fate determination [2]. In vertebrates, there are ~250–300 proteins encoded by Homeobox genes. These proteins can be classified into different subgroups based on their DNA-binding domain and protein-protein interaction domain, such as HOX, ANTP, PRDHOX, LIM, SIX, and CUT [3–5]. The classical Homeobox genes are the *HOX* genes which consist of 39 genes in humans and mice. These *HOX* genes are divided into four paralogous clusters (*HOXA* through *HOXD*), with each cluster numbered from 1 to 13 and located linearly on one chromosome. This genomic organization of the *HOX* gene clusters is correlated with their transcriptional activity, where those at the 3′ end are expressed in more anterior regions of the embryo while those at the 5′ end are expressed in more posterior regions- a phenomenon known as spatial collinearity. Additionally, the activation of *HOX* genes follows a temporal collinearity pattern where those at the 3′ end of each cluster are activated first, followed by those located at more 5’ positions [6]. The spatial-temporal collinearity of *HOX* plays a crucial role in embryogenesis by regulating axial elongation and setting up the schematization of neural tube, somite, heart, etc.

UBE3D (Ubiquitin protein ligase E3D), previously identified as an E3 ubiquitin ligase due to its HECT-like sequence at the C-terminus, has been shown to ubiquitinate Cyclin B in vitro [7]. The yeast homolog of UBE3D, Ipa1, can interact with several ubiquitin-conjugating enzymes (E2s), and the activity of the ubiquitin-proteasome system was decreased in Ipa1-depleted cells [8]. A recent study showed that UBE3D can ubiquitinate 3βHSD1 in prostate cancer cells [9]. These evidences suggest the potential role of UBE3D as an E3 ligase participating in the proteasome degradation pathway. Surprisingly, two separate studies have revealed that UBE3D can stabilize CPSF3 (cleavage and polyadenylation specific factor 3) in 3T3-L1 cells and breast cancer cells [10, 11]. However, the mechanism by which UBE3D stabilizes CPSF3 remains unknown.

Recently, UBE3D has been identified as an important protein associated with age-related macular degeneration in humans, and the retina phenotype can be replicated in *Ube3d*^*+/−*^ mice [12–14]. Notably, Huang et al. reported the absence of *Ube3d*^*−/−*^ mice in offspring [12], indicating the crucial role of UBE3D in mouse embryonic development. However, the specific function of UBE3D during embryonic development remains unknown.

In our study, we generated *Ube3d* knockout mice and observed that *Ube3d*^*−/−*^ mice exhibit developmental delays at E8.5. Severe growth retardation and malformation were evident in *Ube3d*^*−/−*^ mice from E9.5 to E11.5, with over 80% *Ube3d*^*−/−*^ mice died between E10.5 and E11.5. UBE3D is primarily expressed in the neural tube, heart, and limb buds at E9.5–11.5. Deletion of *Ube3d* resulted in significant downregulation of multiple Homeobox genes at the transcriptional level. Furthermore, we found that UBE3D interacts with the endonuclease CPSF3 and stabilizes it by de-ubiquitination. Deficiency of UBE3D resulted in CPSF3 deficiency in both mouse and human cells. Overexpression of dominant negative mutants of CPSF3 in HEK 293T cells can partially reduce the mRNA levels of several Homeobox genes. Overall, our study has uncovered a novel and critical role of UBE3D in regulating CPSF3/Homeobox genes during early embryonic development in mice.

## Results

### *Ube3d*^*−*/*−*^ mice exhibited significant developmental delays and malformations during E8.5–E11.5

**Fig. 1:**
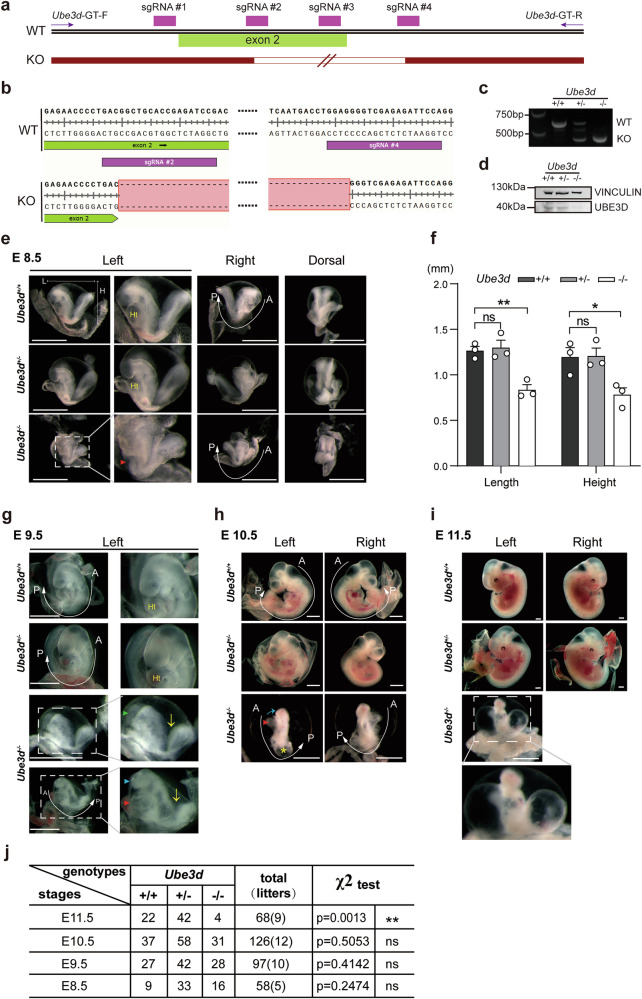
Construction of *Ube3d* knockout mouse and phenotypes between E8.5 and E11.5. **a** The four sgRNAs used for *Ube3d* knockout were illustrated here. **b** The allele sequences around exon2 in both wild-type (WT) and knockout (KO) were presented. **c** Genotyping of *Ube3d*^*+/+*^, *Ube3d*^*+/−*^, and *Ube3d*^*−/−*^ mice was performed using PCR. **d** Protein expression of UBE3D in E10.5 mouse was examined by immunoblotting, with VINCULIN as a loading control. **e**–**i** The phenotypes of mice with different genotypes at E8.5, E9.5, E10.5, and E11.5 were observed and documented, including measurements of embryo length (L) and height (H) at E8.5 (**f**). Statistical analysis in (**f**) was performed using a two-sided unpaired *t*-test (*p < 0.05, **p < 0.01, ns not significant). A anterior, P posterior, Ht heart. All scale bars are 1 mm. Red arrowhead: abnormal heart; green arrowhead: open forebrain; blue arrowhead: small head; yellow arrow: open and unturned caudal neural tube. **j** Quantification of embryos from intercrossing *Ube3d*^+/*−*^ mice at stages E8.5-11.5 was conducted and analyzed using a Chi-square test for significance based on expected Mendelian frequencies (ns not significant; **p < 0.01).

We previously screened a mutagenesis mouse library generated by piggyBac (PB) transposon in IDM at Fudan University [15] and observed that *Ube3d*^PB/PB^ mice are embryonic lethal and exhibit multiple defects at E10.5, including growth retardation and unturned caudal neural tube (Fig. S1). To investigate the role of UBE3D in embryogenesis, we generated *Ube3d*^*+/−*^ mice through the deletion of exon2 of *Ube3d* using the CRISPR/Cas9 technique. Figure 1a illustrated the sequence alignment of the four sgRNAs used for *Ube3d* knockout, while the WT and KO allele sequences were shown in Fig. 1b. The *Ube3d*^*+/−*^ mice were viable and appeared normal. We crossed *Ube3d*^*+/−*^ mice to obtain *Ube3d*^*−/−*^ embryos, and collected the yolk sacs for genotyping (Fig. 1c). Total RNA was isolated from E10.5 embryos and reverse transcribed for use as a template in PCR with a pair of primers spanning from exon2 to exon6. The results confirmed the absence of matured *Ube3d* mRNA in *Ube3d*^*−/−*^ embryos (Fig. S2). Protein extraction was performed from E10.5 embryos, and immunoblotting analysis revealed almost undetectable levels of UBE3D protein in *Ube3d*^*−/−*^ embryos (Fig. 1d), confirming the successful knockout of UBE3D in these embryos.

During the mouse developmental process, the period from E8.5 to E11.5 is critical for neurulation [1]. To investigate the impact of UBE3D deficiency on neurulation, embryos were collected during this time frame for phenotype observation. Heterozygote (HET, *Ube3d*^*+/−*^) embryos appeared similar to wild-type (WT, *Ube3d*^*+/+*^) littermates at all stages (Fig. 1e–i). However, homozygote (HOMO, *Ube3d*^*−/−*^) embryos exhibited significant abnormalities at all the investigated stages. The size of E8.5 HOMO embryos is smaller than the WT and HET littermates, with a 34% reduction in height and length (Fig. 1e, f). Unlike the WT and HET littermates, the heart tube in HOMO embryos is not visible at E8.5 (Fig. 1e, red arrowhead). By E9.5, the HOMO embryos displayed more severe developmental delay and malformation (Fig. 1g), characterized by reduced size and incomplete turning along the A-P axis (Fig. 1g, yellow arrow). Additionally, approximately 20% of the HOMO embryos exhibited unclosed forebrain (Fig. 1g, green arrowhead), and despite partial closure in other cases, there was a lack of brain vesicles (Fig. 1g, blue arrowhead). Furthermore, the hearts of HOMO embryos were underdeveloped with reduced blood supply (Fig. 1g, red arrowhead). At E10.5, HOMO embryos displayed exacerbated growth retardation and malformation compared to their WT and HET littermates. In addition to reduced size and unfinished turning, the HOMO embryos exhibit incomplete heart looping and pericardial swelling even though the hearts continued beating upon dissection (Fig. 1h, red arrowhead). They also showed underdeveloped brain vesicles (Fig. 1h, blue arrow) and unfinished embryo turning at the posterior region (Fig. 1h, A-P axis). A swollen vesicle was presented at the posterior end (Fig. 1h, yellow star). Unlike WT and HET embryos, the limb buds are not visible in the HOMO embryos at this stage (Fig. 1h). At E11.5, only 4 HOMO embryos were collected from 9 litters with similar morphological abnormalities observed at E10.5 (Fig. 1i). Despite these severe abnormalities, Chi-squared analysis indicated consistent Mendelian single gene inheritance ratios for WT, HET, and HOMO embryos at E8.5–10.5; however, there was a significant deviation at E11.5 (Fig. 1j). Thus, most HOMO embryos perish between E10.5 and E11.5 due to these development defects.

Our phenotypic data indicate that HOMO mice exhibit developmental delays at E8.5 and have more severe growth retardation and malformation during E9.5–10.5. Our findings highlight the essential role of *Ube3d* in neurulation and morphogenesis in mice.

### The expression patterns of UBE3D in mice at E9.5–E11.5

**Fig. 2:**
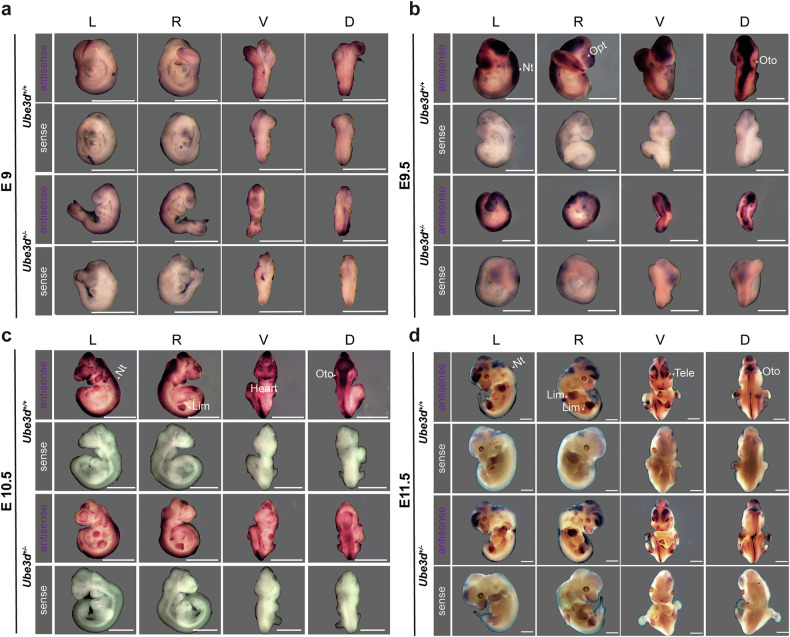
ISH of *Ube3d* in mice from E9.0 to E11.5. The mRNA expression of *Ube3d* in mice at **a** E9.0, **b** E9.5, **c** E10.5, and **d** E11.5 was detected using whole-mount in situ hybridization, and representative figures are presented here. *N* ≥ 3 embryos were used for each genotype of mice at each stage. The RNA probes were labeled with digoxigenin. The sense probe was used as a negative control. L left view, R right view, V ventral view, D dorsal view, Nt neural tube, Opt optic pit, Oto otocyst; Tele telencephalon, Lim limb buds. All scale bars are 1 mm.

**Fig. 3:**
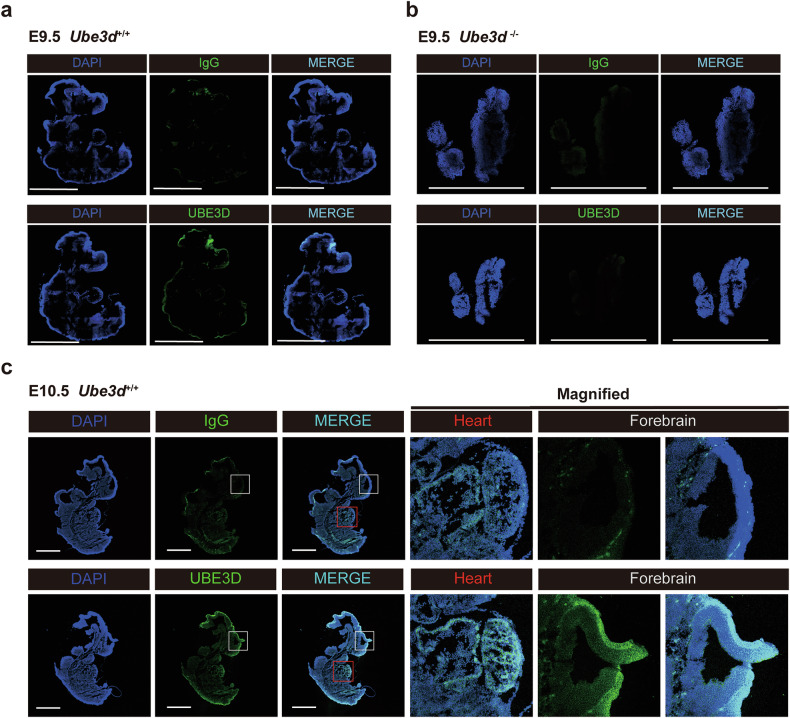
IF staining of UBE3D in mice from E9.5 to E11.5. The protein expression of UBE3D was detected through immunofluorescent staining on sagittal sections from **a** E9.5 WT embryos and **b** E9.5 HOMO embryos; or **c** E10.5 WT embryos. The heart and forebrain of E10.5 WT embryos were magnified to show the detailed staining pattern. Representative figures are shown here, and *N* ≥ 3 embryos were used at each stage. All scale bars are 1 mm.

We then conducted in situ hybridization and immunofluorescence experiments to analyze the expression patterns of UBE3D in E9.0-11.5 mice. Single-stranded RNA probes labeled with digoxigenin were utilized for whole-mount in situ hybridization on WT and HET embryos during E9.0-11.5, revealing no significant difference between HET embryos and WT littermates at all stages (Fig. 2). An intermediate signal (light purple) could be detected in the neural tube at E9.0 (Fig. 2a). Strong signals (purple) were detected in the neural tube and optic pit at E9.5–10.5 (Fig. 2b), as well as in the heart and limb buds at E10.5 (Fig. 2c). By E11.5, a high signal was observed in forebrain and limb buds (Fig. 2d). Furthermore, immunofluorescence staining on the sagittal sections of E9.5–10.5 WT embryos showed a prominent UBE3D signal in the neural tube at both time points (Fig. 3a, c). No UBE3D signal was detected on E9.5 HOMO embryos (Fig. 3b). The forebrain and heart of the E10.5 WT embryo was magnified in Fig. 3c to show the expression of UBE3D. These expression patterns of UBE3D are consistent with the phenotypes observed in HOMO embryos.

### Multiple Homeobox genes were downregulated at E10.5 in *Ube3d*^*−*/*−*^ mice

**Fig. 4:**
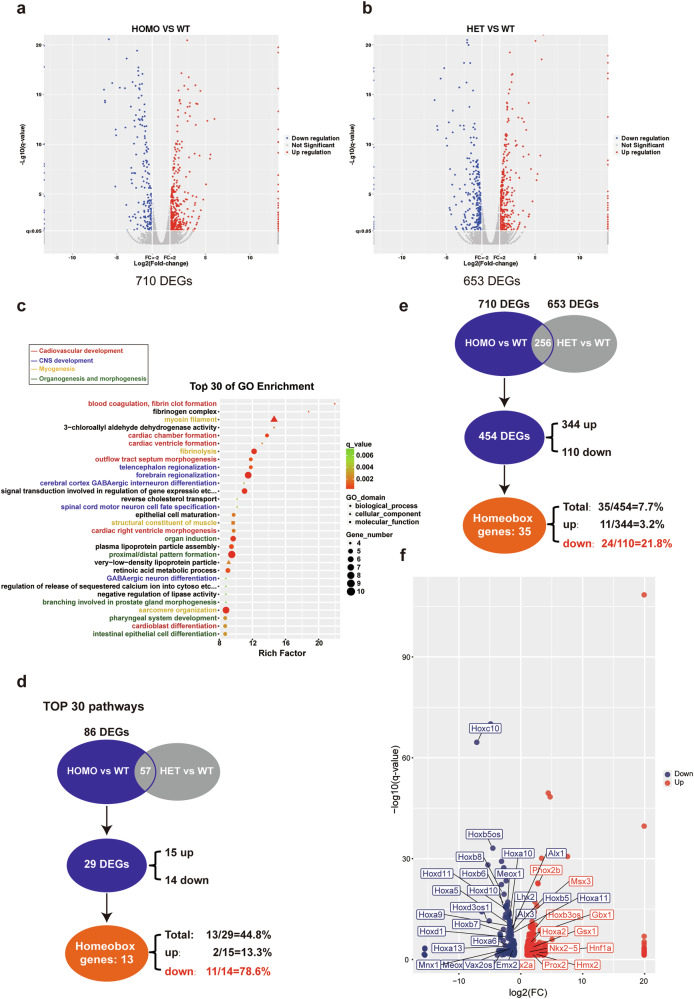
Transcriptome analysis of mice at E10.5 revealed downregulation of multiple Homeobox genes in HOMO mice. Volcano plots were generated to compare mRNA expression in HOMO versus WT mice (**a**) and HET versus WT mice (**b**), using a minimum of seven embryos for each genotype. DEGs (differentially expressed genes) were identified using the following criteria: Fold change = 2, *q* value ≤0.05. A total of 710 DEGs were found between HOMO and WT mice, and 653 DEGs between HET and WT mice. **c** GO enrichment analysis was conducted on the 710 DEGs between HOMO and WT mice, revealing the TOP 30 pathways here. **d** Intersection analysis was carried out on the 86 DEGs enriched in the TOP 30 pathways, revealing 29 DEGs uniquely detected between HOMO and WT mice. Among these 29 unique DEGs, there are 11 upregulated genes and 14 downregulated genes. Notably, out of the 14 downregulated genes, 78.6% (11/14) are Homeobox genes. **e** Intersection analysis was also performed between the 710 DEGs and 653 DEGs, which revealed that there are 454 unique DEGs between HOMO and WT mice. Among these 454 DEGs, there are 344 upregulated genes and 110 downregulated genes; and among them, there are 11 upregulated Homeobox genes and 24 downregulated Homeobox genes, respectively. **f** These 35 Homeobox genes are marked on a scatter diagram of 454 DEGs.

Since the HOMO embryos at E10.5 are still viable and the ratios for WT, HET, and HOMO embryos at E10.5 continue to adhere to Mendelian single gene inheritance patterns, whole embryos were collected at E10.5 for RNA-seq experiments. A total of 710 differentially expressed genes (DEGs) were identified between HOMO and WT embryos (Fig. 4a and Table S1), as well as 653 DEGs between HET and WT embryos (Fig. 4b and Table S2). There are 256 DEGs identified in both HOMO vs WT and HET vs WT embryos (Table S3). GO enrichment analysis on those 710 DEGs between HOMO and WT embryos revealed that the top 30 pathways are predominantly involved in cardiovascular development, central nervous system development, myogenesis, organogenesis, and morphogenesis (Fig. 4c), which aligns with the observed phenotypes in HOMO mice. We then further analyzed the 86 DEGs between HOMO and WT mice within the top 30 pathways. Given the similarity of HET embryos to their WT littermates at E10.5, we excluded genes detected between HET and WT embryos, resulting in 29 DEGs (15 upregulated and 14 downregulated) uniquely changed in HOMO mice. We noticed that 78.6% of the downregulated genes (11/14) are Homeobox genes; while there are only two Homeobox genes within the group of upregulated genes (2/15) (Fig. 4d and Table 1).

**Table 1:** The list of Homeobox genes identified to be downregulated and upregulated in *Ube3d*^*−/−*^ mice at E10.5, within the TOP30 pathways.

Gene name	Log2FC (vs WT)	*p*_value	*q*_value	Up/down
*Hoxa5*	−1.824655347	1.60E-13	2.44E-11	Down
*Hoxa9*	−1.768991881	7.88E-13	1.09E-10	Down
*Hoxa10*	−1.867918238	9.97E-15	1.65E-12	Down
*Hoxa11*	−1.58023	1.81E-10	2.00E-08	Down
*Hoxa13*	−1.998732667	1.91E-06	0.000113586	Down
*Hoxc10*	−7.113478502	5.12E-69	2.48E-65	Down
*Hoxd10*	−2.474942494	8.48E-19	2.28E-16	Down
*Hoxd11*	−2.19639009	5.28E-18	1.28E-15	Down
*Emx2*	−1.124200335	5.48E-06	0.000298875	Down
*Lhx2*	−1.608237327	1.42E-12	1.95E-10	Down
*Mnx1*	−1.879437671	1.29E-05	0.000643992	Down
*Hnf1a*	2.534976	0.000422	0.011746	Up
*Nkx2-5*	1.074782	0.000187	0.006204	Up

We then performed a similar analysis for the 710 DEGs between HOMO and WT mice. After excluding genes also detected between HET and WT embryos, there are 454 DEGs (344 upregulated and 110 downregulated) uniquely changed in HOMO mice. Among the 454 DEGs, there are 35 Homeobox genes. Again, the downregulated group contains a higher proportion of Homeobox genes, with 21.8% (24/110) of the downregulated genes being classified as such, compared to only 3.2% (11/344) within the upregulated genes group (Fig. 4e and Table S4). These 35 Homeobox genes are marked on a scatter diagram of 454 DEGs (Fig. 4f).

**Fig. 5:**
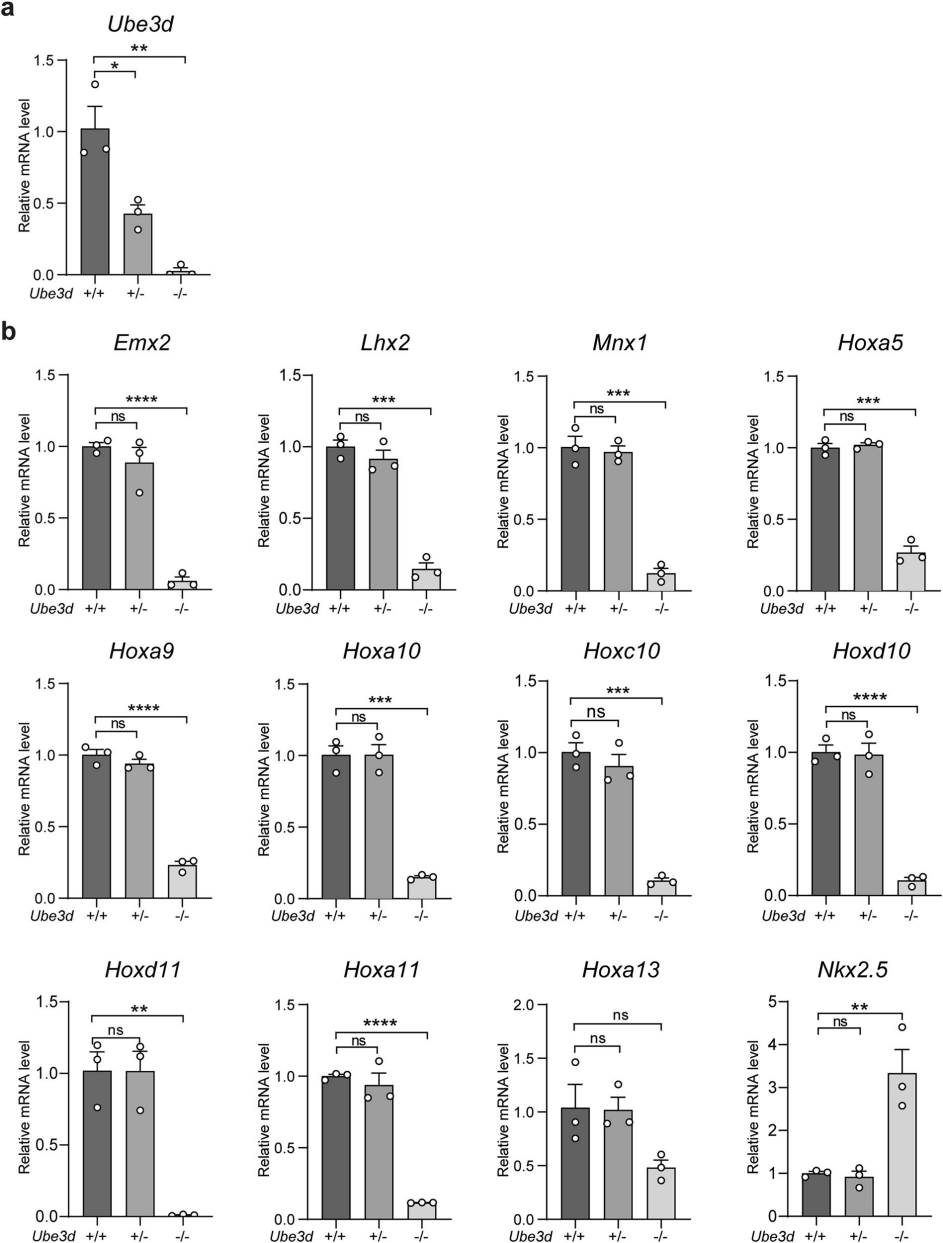
The mRNA expression of 11 downregulated Homeobox genes in E10.5 mice. Total mRNA was extracted from fresh E10.5 embryos for qRT-PCR analysis to confirm the relative mRNA expression of *Ube3d* (**a**), the 11 downregulated Homeobox genes, and one upregulated *Nkx2-5* (**b**), with *Gapdh* serving as the internal control. In each experiment, at least 3 embryos per genotype were pooled together in each sample, and three independent experiments were conducted. The data were presented as mean ± SEM, and statistical analysis was performed using a two-sided unpaired *t*-test (*p < 0.05, **p < 0.01, ***p < 0.001. ns not significant).

The particularly high ratio of Homeobox genes among the downregulated genes, especially within the top 30 pathway genes, has piqued our interest, prompting further investigation into the abnormal expression of multiple Homeobox genes in UBE3D deficient embryos. As 78.6% (11/14) of the downregulated genes within the top 30 pathways are Homeobox genes, we then proceeded to confirm the downregulation of these 11 Homeobox genes in E10.5 HOMO embryos. Total mRNA was collected from WT, HET, and HOMO embryos at E10.5 for qRT-PCR experiments. Due to the small size of HOMO embryos, at least 3 embryos of the same genotype were pooled together for each test. The mRNA levels of UBE3D were reduced by ~50% in HET embryos compared to their WT littermates and were nearly undetectable in HOMO embryos (Fig. 5a). In HET embryos, the mRNA levels of those 11 Homeobox genes are similar to their WT littermates; however, in HOMO embryos, 10 out of 11 downregulated Homeobox genes exhibited a significant reduction compared to their WT littermates (Fig. 5b). We also included *Nkx2-5*, one of the upregulated Homeobox genes identified in Fig. 4f in this test and the results confirmed its upregulation in HOMO embryos (Fig. 5b). Among these 11 downregulated Homeobox genes, there are 8 *Hox* genes (*Hoxa5*, *Hoxa9*, *Hoxa10*, *Hoxc10*, *Hoxd10*, *Hoxa11*, *Hoxd11,* and *Hoxa13*), with 5 of them specifically expressed at the lumbar/sacral region (*Hoxa10*, *Hoxa11*, *Hoxc10*, *Hoxd10*, and *Hoxd11*) [16]. The other 3 downregulated Homeobox genes are *Emx2*, *Lhx2*, and *Mnx1*. It has been reported that *Lhx2* and *Emx2* are essential for forebrain development [17–20]. These results are consistent with the observed phenotypes (small head and unturned caudal neural tube) in HOMO embryos. We also conducted similar qRT-PCR experiments in E8.5 embryos (Fig. S3). At this stage, only 5 out of 11 Homeobox genes were detectable. Once again, the expression levels of *Hoxa5*, *Hoxa9*, *Lhx2*, and *Mnx1* were significantly lower in the HOMO embryos compared with those in the WT and HET littermates (Fig. S3b). Taken together, UBE3D deficiency resulted in a significant decrease in multiple Homeobox genes during this period, which are essential for morphogenesis at this stage of mouse embryo development.

### Deficiency of UBE3D impairs CPSF3, and UBE3D decreases the level of polyubiquitination of CPSF3

In order to investigate the molecular function of UBE3D, we previously conducted yeast two-hybrid screening [21] and searched the BioPlex Network [22, 23] for protein interactions to unbiasedly identify potential targets of UBE3D. CPSF3 is the only protein identified by both our screening and the database [21]. Our findings indicate that deficiency of UBE3D results in reduced mRNA levels of multiple Homeobox genes in vivo. Given that CPSF3 is an endonuclease involved in mRNA maturation as a subunit of the CPSF complex [24–26], we aimed to explore its role in the downregulation of Homeobox genes in UBE3D deficient mice.

**Fig. 6:**
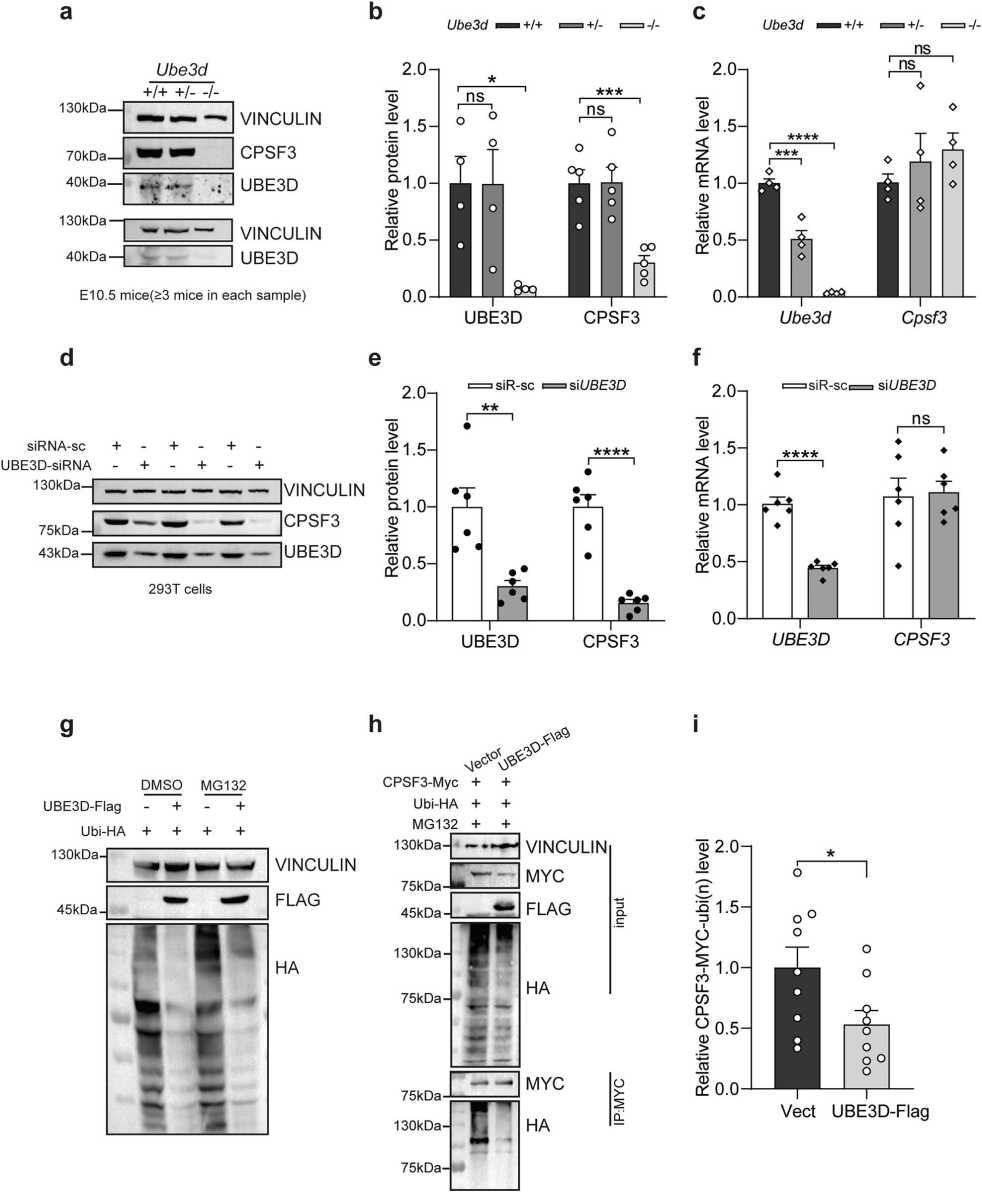
UBE3D deficiency results in the elimination of CPSF3 protein, and UBE3D decreases the level of polyubiquitination of CPSF3. Expression of UBE3D and CPSF3 was examined in mice at E10.5 using western blot analysis, with representative figures shown in (**a**) and quantification of UBE3D and CPSF3 protein levels presented in (**b**). **c** The mRNA levels of *Cpsf3* and *Ube3d* in E10.5 mice was assessed by qRT-PCR. Protein levels were normalized to VINCULIN. mRNA levels were normalized to *Gapdh*. At least three independent experiments were conducted, each including at least three embryos per lane. **d**–**f** UBE3D was knocked down using siRNA in HEK 293T cells. The expression of UBE3D and CPSF3 protein levels were evaluated by immunoblotting. Representative figure is shown in (**d**) and quantification is shown in (**e**). The mRNA levels of *CPSF3* and *UBE3D* in HEK 293T cells were also examined by qRT-PCR and normalized to *ACTB* (**f**). A total of *N* = 6 repeats were included for both (**e**) and (**f**). Data were presented as mean ± SEM. Statistical analysis was performed using a two-sided unpaired *t*-test. (*p < 0.05, **p < 0.01, ***p < 0.001. ns not significant). **g** HEK 293T cells were transfected with either vector or UBE3D-Flag plus Ubiquitin-HA plasmids, and polyubiquitination of total protein were detected using an anti-HA antibody. Cells treated with or without MG132 were both collected. **h** Immunoprecipitation was used to pull down CPSF3-Myc using anti-Myc antibody after transfection with UBE3D-Flag, Ubiquitin-HA, and CPSF3-Myc plasmids in HEK 293 T cells; subsequently, the polyubiquitination level of CPSF3 was detected using an anti-HA antibody. **i** The polyubiquitination level of CPSF3 with UBE3D overexpression was quantified (*N* = 9).

Previous studies have suggested that UBE3D is capable of stabilizing CPSF3 [10, 11]. Therefore, we first validated the capacity of UBE3D to stabilize CPSF3 in our experimental model. We collected WT, HET, and HOMO embryos at E10.5 for both immunoblotting and qRT-PCR experiments. Again, the levels of UBE3D protein were comparable between HET embryos and their WT littermates, while it was significantly diminished in HOMO embryos compared to the WT littermates (Fig. 6a, b). The reduced mRNA did not affect the protein expression level of UBE3D in HET embryos (Fig. 6a–c). The level of CPSF3 protein was comparable between HET embryos and their WT littermates, while it was significantly diminished in HOMO embryos compared to the WT littermates (Fig. 6a, b). In contrast, the mRNA expression of *Cpsf3* remained unaffected (Fig. 6c). Furthermore, we investigated whether RNAi knockdown of UBE3D could also reduce CPSF3 expression in vitro. UBE3D-specific siRNA was transfected into HEK 293T cells, followed by immunoblotting and qRT-PCR experiments. Unlike the HOMO embryos, UBE3D-siRNA can effectively knock down the mRNA levels of UBE3D in HEK 293T cells, and the protein levels of UBE3D are also significantly decreased (Fig. 6d–f). CPSF3 protein but not mRNA was significantly reduced when UBE3D was effectively knocked down (Fig. 6d–f). Both in vivo and in vitro data confirm that UBE3D plays a role in stabilizing the CPSF3 protein.

Next, we investigated the mechanism by which UBE3D stabilizes CPSF3. Firstly, we assessed the level of polyubiquitination in total protein from HEK 293T cells overexpressing UBE3D. Surprisingly, our findings revealed a decrease in the overall polyubiquitination levels with UBE3D overexpression, and this de-ubiquitination function remains unaffected by MG132 treatment (a proteasome inhibitor) (Fig. 6g). Furthermore, analysis of CPSF3 polyubiquitination level in UBE3D-overexpressing cells also showed a significant decrease (Fig. 6h, i). These results suggest that UBE3D may act as a de-ubiquitinating enzyme, particularly with regard to CPSF3.

**Fig. 7:**
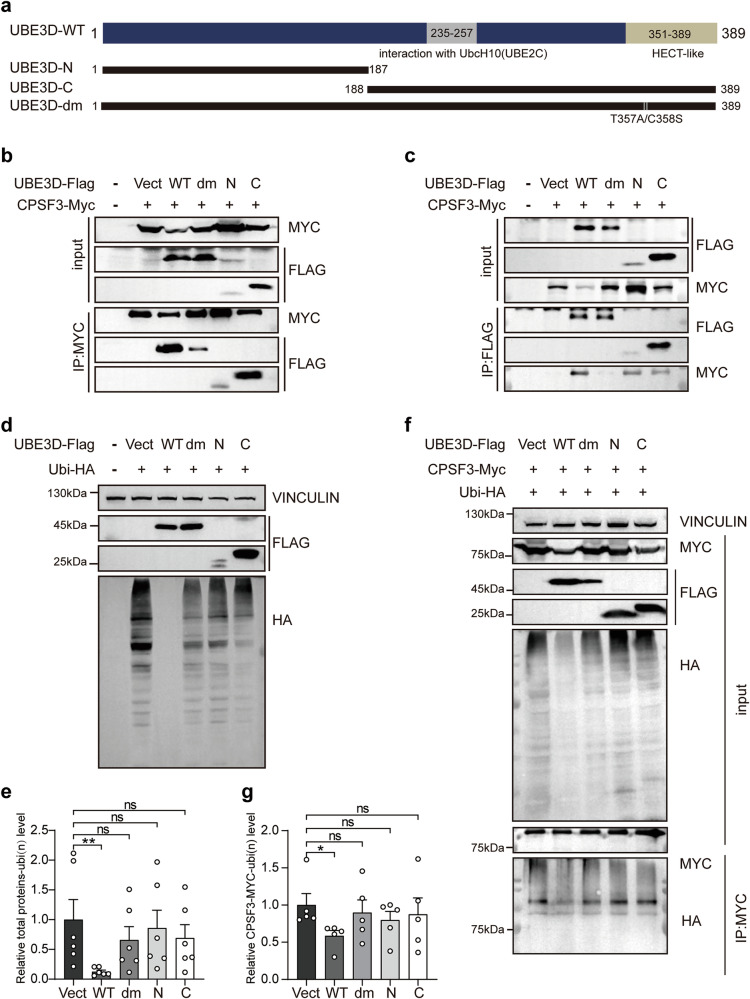
UBE3D mutants interact with CPSF3 without reducing its polyubiquitination. **a** The schematic diagram illustrates different UBE3D mutants, including UBE3D-N (1–187 a.a.); UBE3D-C (188–389 a.a.), and the dominant negative mutant UBE3D-dm with T357A/C358S double mutations in the HECT-like domain. Co-IP experiments were conducted to examine the interaction between CPSF3 and various UBE3D mutants. HEK 293T cells were co-transfected with CPSF3-Myc and either UBE3D or its mutants, followed by co-IP experiments using anti-Myc (**b**) or anti-Flag (**c**) antibodies. **d**–**g** HEK 293T cells were transfected with Ubiquitin-HA and UBE3D or its mutants and treated with MG132. Polyubiquitination of the total protein (**d**) and CPSF3 (**f**) were detected using an anti-HA antibody. **e** The level of polyubiquitination for the total protein was quantified using HA and normalized against an empty control. **g** The level of polyubiquitination for CPSF3 was quantified using HA and normalized by MYC in IP groups.

According to previous research [7], the presumptive HECT-like domain is located at the C-terminus of UBE3D. Consequently, we generated two truncated forms of UBE3D, UBE3D-N (1–187a.a) and UBE3D-C (188–389a.a). Additionally, a dominant negative mutant of UBE3D (UBE3D-dm) was constructed by introducing mutations in two amino acids (T357A and C358S) within the HECT-like domain (Fig. 7a). Various mutants of UBE3D were overexpressed with CPSF3 in HEK 293T cells, followed by co-immunoprecipitation (co-IP) experiments to assess their interaction with CPSF3. The results indicated that CPSF3 is capable of interacting with both wild-type and mutated UBE3D (Fig. 7b, c). The interaction between CPSF3 and UBE3D-N or -C was not detected in immunoprecipitation experiments when IgG was used (Fig. S4). However, it was observed that the dominant negative mutant UBE3D-dm exhibited a weaker interaction with CPSF3 compared to the wild-type UBE3D (Fig. 7b, c). Additionally, ubiquitination assays performed in those cells revealed that only overexpression of wild-type UBE3D resulted in a reduction of the polyubiquitination levels in total protein and CPSF3. Conversely, none of the UBE3D mutants displayed this effect on either total protein polyubiquitination levels (Fig. 7d, e) or CPSF3 polyubiquitination levels (Fig. 7f, g). Therefore, all mutations of UBE3D abolish its de-ubiquitination function on CPSF3, despite their ability to interact with CPSF3.

### Dominant negative mutants of CPSF3 partially reduce the mRNA of several Homeobox genes

**Fig. 8:**
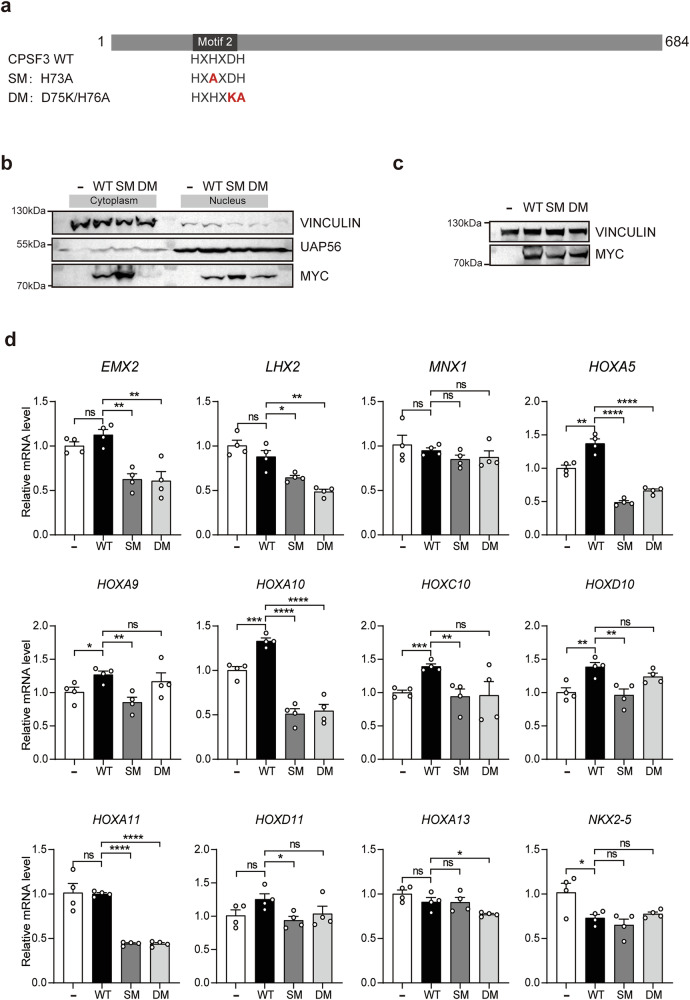
Overexpression of CPSF3 mutants resulted in a partial reduction in the mRNA levels of several Homeobox genes. **a** The schematic diagram illustrates CPSF3 WT and its mutants, including SM (single mutation, H73A) and DM (double mutations, D75K/H76A). **b** HEK 293T cells were transfected with either WT or mutant CPSF3-Myc plasmids. Subsequently, nuclear and cytoplasmic proteins were separated for immunoblotting experiments to detect the presence of different CPSF3 mutants in either nuclear or cytoplasmic components. **c**, **d** HEK 293T cells were transfected with either WT or mutated CPSF3. The expression levels of CPSF3 and its mutants are presented in (**c**). **d** Total mRNA was extracted for quantification of relative expression levels of 11 downregulated Homeobox genes plus the upregulated NKX2-5 identified in HOMO mice using qRT-PCR, which was normalized to *GAPDH*. Data were presented as mean ± SEM. Statistical analysis was performed using a two-sided unpaired *t*-test (*p < 0.05, ***p < 0.001, ****p < 0.0001. ns not significant).

Next, we aim to investigate whether CPSF3 deficiency also results in a decrease in mRNA levels of the Homeobox genes. However, the endogenous expression of CPSF3 is abundant in HEK 293T cells, and we were unable to achieve knockdown using RNAi. Therefore, we opted to utilize dominant negative CPSF3 for this experiment. It has been reported that CPSF3 functions as a metallo-β-lactamase, which is activated by Zn^2+^. Motif 2 of CPSF3 is critical for its Zn^2+^ binding, and mutation of motif 2 can lead to CPSF3 inactivation, resulting in impaired pre-mRNA cleavage and maturation [27, 28]. Based on those reports, we generated two dominant negative mutants of CPSF3, namely CPSF3-SM (single mutation, H73A) and CPSF3-DM (double mutations, D75K/H76A) (Fig. 8a). Both mutants were successfully expressed in HEK 293T cells. Nucleocytoplasmic separation experiments revealed that both wild-type and mutant forms of CPSF3 were effectively transported into the nucleus (Fig. 8b). Subsequently, wild-type or mutated forms of CPSF3 were overexpressed in HEK 293T cells. The protein levels of wild-type and mutants forms of CPSF3 were assessed by immunoblotting (Fig. 8c). Total RNA of these cells was extracted for qRT-PCR analysis of 11 downregulated Homeobox genes and the upregulated *NKX2-5* identified in HOMO embryos. The results revealed that, with the exception of *MNX1* and *NKX2-5*, the mRNA levels of the other Homeobox genes were significantly reduced by overexpression of at least one dominant negative mutant form of CPSF3 (Fig. 8d). Specifically, the mRNA levels of five Homeobox genes (*EMX2*, *LHX2*, *HOXA5*, *HOXA10*, and *HOXA11*) were significantly decreased by both dominant negative mutants of CPSF3 (Fig. 8d). These findings suggested that the mRNA levels for several Homeobox genes can be partially reduced by dominant negative mutants of CPSF3.

## Discussion

In this study, we generated *Ube3d* knockout mice and observed that HOMO mice exhibited severe growth retardation and malformation, particularly in the brain, neural tube, heart, and limb buds, leading to their death between E10.5 and E11.5. In contrast to WT and HET littermates, most HOMO embryos displayed a small head and unturned caudal neural tube from E9.5 onwards. The deficiency of UBE3D resulted in a dramatic reduction of multiple Homeobox genes in vivo.

In *Ube3d* null embryos, 11 out of the 14 downregulated genes in the top 30 pathways were Homeobox gene. This enrichment of Homeobox genes among the downregulated genes caught our attention. Homeobox genes play a crucial role in vertebrate embryogenesis, particularly in A-P somatogenesis and organogenesis. Among these downregulated Homeobox genes, there are eight *Hox* genes, with five corresponding to the lumbosacral region in mice: *Hoxa10*, *Hoxa11*, *Hoxc10*, *Hoxd10*, and *Hoxd11*. It has been reported that the paralogous genes *Hox10* and *Hox11* are essential for mammalian skeleton patterning. Specifically, *Hox10* genes are needed for lumbar vertebrae, while *Hox11* genes prevent sacral vertebrae to become lumbar vertebrae [29]. However, there is no report on what happens when both *Hox10* and *Hox11* are non-functional. Based on our study, it is highly likely that the absence of most of these paralogous genes would significantly impact caudal neural tube development.

In addition to the 8 *Hox* genes, there are three other Homeobox genes: *Emx2*, *Lhx2*, and *Mnx1*. *Emx2* is crucial for forebrain development. Suda et al. reported that *Emx2* is expressed in the caudal forebrain primordium at E8.5 and dorsal telencephalon at E9.5-E10.5 in mouse [30]. Kimura et al. found that *Emx2* was involved in caudal forebrain primordium specialization as the caudal forebrain remained unspecified in *Emx2*^*−*/*−*^*Pax6*^*−*/*−*^ double mutant mice at E9.5 [31]. *Lhx2* also plays an essential role in forebrain and telencephalon development [32, 33]. *Lhx2* has a time-specific function in cortical progenitors: before E10.5, it functions as a cortical selector and suppresses hem and anthem formation; between E10.5 and E11.5, it suppresses the formation of an ectopic piriform cortex; after E10.5, it regulates the balance between proliferation and differentiation in cortical progenitors [17]. Both *Emx2* and *Lhx2* are dramatically reduced in HOMO mouse, which may explain why very small and undeveloped forebrains are observed in those mice.

In addition to brain and neural tube abnormalities, we observed severe heart defects in HOMO mice. The *Hox* gene has been linked to cardiovascular development. Lineage tracing experiments demonstrated that anterior *Hox* genes (*Hoxa1*, *Hoxa2*, *Hoxb1*, and *Hoxb2*) are expressed in cardiovascular progenitor cells at E7.5 [34]. More posterior *Hox* genes, including *Hoxa7*, *Hoxa9*, *Hoxb6*, *Hoxc8*, and *Hoxd8* are expressed in the second heart field between E7.5-E9.5 in mice. *Hoxa10* can cooperate with *Nkx2-5* to regulate the timing of cardiac differentiation in vitro [35]. Notably, HOMO mice exhibited significant downregulation of both *Hoxa9* and *Hoxa10* and upregulation of *Nkx2-5*. Therefore, further exploration into the role of UBE3D in cardiovascular development and disease is warranted in the future. Furthermore, *Hox* genes are involved in limb bud development. Xu et al. demonstrated that *Hox5* is essential for forelimb anterior patterning [36]; while all four *Hox9* paralogs (*Hoxa9*, *Hoxb9*, *Hoxc9*, and *Hoxd9*) are necessary for the initial anteroposterior polarization of the early forelimb field [37]. The significant reduction of both *Hoxa5* and *Hoxa9* in HOMO embryos at E10.5 suggests a potential role of UBE3D in regulating early limb bud development by influencing the expression of multiple *Hox* genes. However, the observed defects in HOMO embryos may not solely be attributed to reduced HOX gene expression. For example, *Nkx2-5* is significantly upregulated in HOMO embryos at E10.5. Further investigation is required to elucidate the complete function of UBE3D during embryogenesis, particularly prior to the E8.5 stage.

In our efforts to comprehend the regulatory role of UBE3D in the expression of Homeobox genes, we have observed that UBE3D stabilizes the endonuclease CPSF3 in both mouse and human HEK 293T cells, consistent with previous findings [10, 11]. Interestingly, we have also discovered that UBE3D de-ubiquitinates CPSF3. This de-ubiquitination function of UBE3D on CPSF3 is in good agreement with its stabilization function on CPSF3. However, it is contradictory to its E3 ligase activity on Cyclin B [7] and 3βHSD1 [9]. Why UBE3D can have such opposite functions? One potential explanation for this discrepancy is that UBE3D may exert different functions within distinct protein complexes. A recent structural study has revealed that UBE3D interacts with the metallo-β-lactamase and β-CASP domains of CPSF3; and the conserved Cys144 of UBE3D is positioned in the active site of CPSF3 and can directly coordinate one or both metal ions to stabilize CPSF3 [38]. Unlike CPSF3, neither Cyclin B nor 3βHSD1 possess metal ion binding sites. Further sophisticated investigations will be necessary to elucidate how these metal ions influence the function of UBE3D and to understand the mechanisms underlying its dual/opposite activities.

Nevertheless, several limitations exist in this study. First, we only investigated the embryos between E8.5 and 11.5, and no earlier stages were observed. Whether and how UBE3D affects earlier embryos demands more in-depth research in the future. Second, we focused on Homeobox genes in this study because we noticed that a very high proportion (78.6%) of the downregulated genes within the top 30 pathways are Homeobox genes. However, we are aware that the functions of UBE3D are complex as UBE3D may have other targets apart from CPSF3. Moreover, the substrates of CPSF3 are also numerous. Therefore, the effect of UBE3D KO is very likely to extend beyond Homeobox genes. Last but not least, how CPSF3 recognizes and regulates these Homeobox genes also demands more research as a few Homeobox genes are upregulated in the UBE3D HOMO mice, such as *Nkx2-5* at E10.5. More sophisticated experiments are needed in the future to gain a better understanding of the function of UBE3D during embryonic development.

## Materials and methods

### Mouse maintenance and embryo dissection

*Ube3d*^*+/−*^ mice (C57BL/6 strain) were generated by CRISPR/Cas9 technology and provided by Prof. Jiayu Chen at Tongji University based on previously reported methods [39]. Four single guide RNAs (sgRNAs) were designed to target to the exon2 of *Ube3d* in mouse. The sequences for the sgRNAs are: #1: 5′-ATCAGAGAGAAGTCGTCACT-3′; #2: 5’-CGGATCTCGGTGCAGCCGTC-3′; #3: 5′-TGG TTTACATCTGCGCTTGC-3′; #4: 5′-TGGAATCTCTCGACCCCTCC-3′. The primers used for genotyping are: *Ube3d*-GT-F:5′-ATCTCACGTGGTGATGGCTG-3′, and *Ube3d*-GT-R:5’-TCCGTGAGCC GGACATTTAT-3′. Confirmation of the deletion in knockout mice was verified through Sanger sequencing of the PCR fragment obtained from genotyping. All subsequent analyses were conducted based on genotyping results, without the use of randomization or blinding.

All mice were housed in the animal facility at the Institute of Developmental Biology & Molecular Medicine, Fudan University. The protocol was strictly approved by the Committee on the Ethics of Animal Experiments of Fudan University. All methods were performed in accordance with the relevant guidelines and regulations.

To generate offspring, one male was paired with one or two females in the evening, and vaginal plugs were checked the following morning. Embryonic days were designated as E0.5 on the day the vaginal plug was detected. The embryos were dissected out between E8.5–E11.5 for analysis.

### Embryos protein extraction and immunoblotting

E10.5 embryos were lysed in pre-chilled RIPA lysis buffer (50 mM Tris-HCl pH 7.5, 1% Triton X-100, 0.1% SDS, 1 mM EDTA, and 150 mM NaCl), followed by homogenization at 60 Hz, 4 °C for 120 s using a tissue lyser (Wonbio-C, Shanghai, China). The lysate was then incubated on ice for 10 min and centrifuged at 15,000 rpm and 4 °C for 15 min. The supernatant containing total protein was mixed with 5X SDS-PAGE loading buffer (P0015L, Beyotime, China) and denatured at 95 °C for 5 min. Denatured proteins were separated by SDS-PAGE, electro-transferred to a PVDF membrane (IPVH00010, Millipore, Germany), and probed with primary and secondary antibodies. Blot images were captured using the Tanon™5200CE Chemi-Image System and analyzed using ImageJ. Full-length western blots are included in supplemental material.

### Immunofluorescence staining and microscopy

Embryos were rinsed with 1× PBS and then fixed in 4% paraformaldehyde (4% PFA) at 4 °C overnight. Subsequently, the embryos were equilibrated in a sucrose gradient (5, 15, and 30% sucrose in PBS sequentially) until they sank to the bottom, followed by embedding in OCT (4583, Sakura, USA). The embedded embryos were sectioned into 20–25 µm thickness per section using a frozen microtome (Leica, CM190, Germany). The sagittal sections were mounted on glass slides pre-coated with 0.1 mg/mL poly-lysine. These slides were washed with 1×PBS to remove OCT, followed by a wash in 1× PBST (1XPBS with 0.1% Tween 20) for 5 min. Subsequently, the slides were fixed in 4% PFA for 15–20 min at room temperature and permeabilized with 1× PBST containing 0.5% Triton X-100 at room temperature for another 15 min. Next, the slides were put into preheated Tris-Urea (95 °C) for 5 min and cooled at room temperature naturally for antigen repair. Following this, the slides were then incubated in blocking solution (10% Goat serum, 2% IgG-free BSA, 0.3 M glycine, 0.05% Tween 20, 0.05% Triton X-100) at 4 °C overnight. They were incubated with anti-UBE3D (1:25) and anti-IgG (1:250) at 4 °C overnight, followed by secondary antibody incubation with goat anti-rabbit IgG H&L (Alexa Fluor^®^ 488) (1:1000) and DAPI (1:1000) incubation at 4 °C overnight. All antibodies or dyes used were diluted with a blocking solution. Finally, the sections underwent three rounds of rinsing in 1×PBST for 3 min each before being sealed for further imaging process. Immunofluorescence images of embryonic sections were taken using a Zeiss LSM 700 confocal microscope.

### Whole embryo in situ hybridization

Single-stranded RNA probes targeting *Ube3d* were designed using NCBI-Primer BLAST, spanning from exon2 to exon6 (231-753 on cDNA of UBE3D). Transcriptional templates of the probes were obtained by reverse transcribing DNA from NIH3T3 cells, followed by inserting the products into the pGEM-T vector using T-A clonal technology. The primers used for probe cloning are: *Ube3d*-probe-F:5′-TTTGGTGAG AACCCCTGACG-3′ and *Ube3d*-probe-R: 5′-TCCCTCAGATGGCCGGATAA-3′. In vitro transcription of single-strand RNA was carried out using MEGAshortscript^TM^ Kit (AM1354, Invitrogen, USA) according to the manufacturer’s instruction. Both sense and antisense strands were transcribed, and the sense probe was used as the negative control. Whole-mount embryo in situ hybridization procedure was based on previous literature [40]. The RNA probes were labeled with digoxigenin (DIG-11-UTP, 3359247910, Roche) and detected using anti-digoxigenin antibodies conjugated with alkaline phosphatase (Anti-Digoxygenin-AP Fab fragments, 11093274910, Roche). Incubation with the chromogenic substrate (BM Purple AP Substrate, 11442074001, Roche) resulted in the development of a purple color indicating the expression of *Ube3d* in the tissue.

### RNA sequencing

The entire E10.5 mouse embryos were dissected and collected on ice, followed by extraction of total RNA and fragmentation for cDNA library construction. The libraries were subjected to quality control and sequencing on the Illumina NovaSeq6000 platform with a paired-end 150 base pair mode. The raw reads for each sample exceed 47 million, with an approximate data volume of 6G per sample. Differential expressive genes were determined based on a fold change >2 plus *q* value <0.05, and further analyzed for Gene Ontology (GO) enrichment.

### Antibodies

anti-UBE3D (1:500, ab121927, Abcam, UK), anti-CPSF73 (1:5000, ab72295, Abcam), anti-Flag (1:1000, AT0022, Engibody, USA), anti-Myc (1:1000, TA1501215, Origene, USA), anti-VINCULIN (1:10000, ab129002, Abcam), anti-UAP56 (1:1000, A9749, ABclonal, China), anti-HA (1:1000, AF2858, Beyotime, China), Horseradish Peroxidase Labeled Goat Anti-Rabbit IgG(H + L) (1:1000, A0208, Beyotime), Horseradish Peroxidase Labeled Goat Anti-Mouse IgG(H + L) (1:1000, A0216, Beyotime), Alexa Fluor® 488-labeled goat anti-Rabbit (1:1000, ab150077, Abcam).

### Cell culture and transfection

The human embryonic kidney cell line 293T (HEK 293T) was purchased from Procell Life Science & Technology Company in Wuhan, China. Regular mycoplasma testing produced consistently negative results. Cells were cultured in DMEM medium supplemented with 10% fetal bovine serum (Procell, Wuhan, China) and 1% penicillin/streptomycin at 37 °C in a 5% CO_2_ incubator. DNA transfection was carried out using Lipo3000 transfection reagent (GK20006, Glpbio, USA) following the manufacturer’s instructions. Small interfering RNA (siRNA) of *UBE3D* were synthesized by GenePharma, Shanghai. The sequence of UBE3D-siRNA is 5′-CCUGCGGUGAAGUCAUAAUTT-3′. siRNA transfection was performed using X-tremeGENE™ siRNA Transfection Reagent (04476093001, Roche).

### Plasmids

Human UBE3D and UBE3D-N (1–187) were constructed by inserting the cDNA of UBE3D and truncated UBE3D (1–187) into the pCMV-Flag vector at the EcoRI/NotI site. UBE3D-C (188–389) was generated by inserting the cDNA of truncated UBE3D (188–389) cDNA into the pCMV-Flag vector at the HindIII/NotI site. UBE3D-dm was constructed by inserting the cDNA of UBE3D-dm (T357A/C358S) into the pCMV-vector at the EcoRI/NotI site.

The human CPSF3 plasmid was purchased from BGI (The Beijing Genomic Institute) with CPSF3 cDNA inserted into pcDNA3.1-Myc-His Vector at the KpnI/EcoRI site. CPSF3-Myc was generated by fusing the Myc tag to CPSF3 and inserting it into a pCMV vector at the HindIII/NotI site. The pCMV vector was generated from the pCMV-Flag vector by Flag tag deletion using PCR and fused at the NotI site. CPSF3-SM-Myc was constructed by fusing the Myc tag to CPSF3-SM(H73A) cDNA and inserting it into the pCMV vector at the HindIII/NotI site. CPSF3-DM-Myc was generated by fusing the Myc tag to CPSF3-DM(D75K/H76A) cDNA and inserting it into the pCMV vector at the HindIII/NotI site. The primers used for cloning are listed in Supplementary Table S5.

### Co-immunoprecipitation (co-IP)

The CPSF3-Myc plasmid was co-transfected with one of the following plasmids: Flag-UBE3D/Flag-UBE3D-N/Flag-UBE3D-C/Flag-UBE3D-dm into HEK 293T cells cultured in 10 cm dishes. A total of 2 µg of each plasmid was used for transfection. After 42 h, 20 µM MG132 (HY-13259, MCE, USA) was added to the medium, and the cells were harvested 6 h later. The cells were lysed in 200 µL co-IP RIPA (50 mM Tris-HCl pH 7.5, 1% NP40, 0.03% SDS, 1 mM EDTA, and 150 mM NaCl). 10% of the lysate was saved for input samples, and the rest were used for a co-IP experiment. In brief, anti-Myc (1:300) or anti-Flag (1:250) antibody was added to the lysate and then filled with co-IP buffer (16.7 mM Tris-HCl pH8.0, 1.1% Triton X-100, 0.01% SDS, 1.2 mM EDTA, 167 mM NaCl) up to a volume of 500 µL. This mix was rotated at 4 °C overnight. Protein A + G Agarose beads (P2055, Beyotime, China) were added and rotated at 4 °C for 3 h. Subsequently, the agarose beads were washed three times in cold 1×PBS and collected by centrifugation at 2500 rpm. The proteins were eluted from the beads using 1× SDS-PAGE loading buffer and denatured at 95 °C for 5 min. The denatured immunoprecipitants were then collected by high-speed centrifugation and analyzed by electrophoresis as described in the immunoblotting section.

### Ubiquitination assays

HEK 293 T cells were transfected with HA-ubiquitin and CPSF3-Myc for the detection of substrate polyubiquitination levels. The control group was transfected with an empty vector, while the experimental group was transfected with Flag-UBE3D. After 48 h, samples were collected and treated with 20 µM MG132 (133407-82-6, MCE) for 6 h before harvest. The cells were washed with ice-cold PBS and then lysed in RIPA buffer (50 mM Tris-HCl pH 7.5, 1% Triton X-100, 2% SDS, 1 mM EDTA, and 150 mM NaCl) at a volume of 100 µL per well. Then the lysates were subsequently denatured at 95 °C for 5 min. Additional RIPA buffer containing protease inhibitor (GK10017, Glpbio, USA) was added to each sample to reach a final volume of 1 mL. The lysates were sonicated (30% amplitude, 3sON/7sOFF) until the solution became clear, followed by incubation on ice for 10 min and centrifugation at 4 °C and 15,000 rpm for another 5 min. A portion of the supernatant (100 µL) was saved for input analysis, while the remaining lysate was incubated with an anti-Myc (1:300) antibody overnight at 4 °C. The following day, protein A + G agarose beads (40–60 µL) were added to the lysate and rotated at 4 °C for an additional 3 h. Finally, the proteins were eluted with 1× SDS-PAGE loading buffer for subsequent immunoblotting experiments.

### RNA extraction and qRT-PCR

Total RNA was extracted from HEK 293T cells with TRIzol reagent (15596026CN, Invitrogen, USA) and chloroform, followed by isopropanol precipitation, 70% RNase-free ethanol wash, and reverse-transcription into cDNA. About 1 µg total RNA was reverse-transcribed into cDNA using the Vazyme reverse-transcription kit (Nanjing, China). Subsequently, the cDNAs were used for qRT-PCR experiments with the ChamQ Universal SYBR qPCR Master Mix kit (Q71102, Vazyme, China) on the LightCycler® 480 II Real-Time PCR System (Roche). Relative expression levels of each target gene were determined using human *ACTB* or *GAPDH* as internal control based on the delta Ct method. All qRT-PCR experiments were conducted at least in triplicate and sample comparisons were assessed using Student’s *t*-test. The primer sequences used in the qRT-PCR assays are listed in Supplementary Table S5.

### Statistical analysis

All experiments were conducted with a minimum of three repetitions, and statistical significance was assessed. Data were presented as mean ± standard error of the mean (SEM) unless otherwise specified. Statistical analyses were carried out using GraphPad Prism software (version 8.0). Differences in significance were determined by two-tailed unpaired Student’s *t*-test for two datasets and one-way ANOVA followed by Bonferroni post hoc test for multiple datasets. *p < 0.05, **p < 0.01, ***p < 0.001, and ****p < 0.0001.

## Supplementary information

Supplementary figures

Supplementary tables

Original Western blots

## Data Availability

The RNAseq datasets presented in this study are available in NCBI online repositories under Bio Project accession number PRJNA1145649. The website for our dataset is https://www.ncbi.nlm.nih.gov/bioproject/PRJNA1145649.
