# Supplementary material for: Deficiency of UBE3D in mice leads to severe embryonic abnormalities and disrupts the mRNA of Homeobox genes via CPSF3
Source: Cell Death Discov. 2025 Mar 12;11:99. doi: 10.1038/s41420-025-02387-y (PMC11904178; doi:10.1038/s41420-025-02387-y)

Fig. S1: The phenotypes observed in *Ube3d*$^{PB/PB}$ embryos at E10.5.

A phenotypic driven screening was previously conducted in our PiggyBac mouse mutagenesis library. Embryos were obtained by crossing *Ube3d*$^{+/PB}$ mice and dissected at E10.5. The embryos presented here are from two different litters.

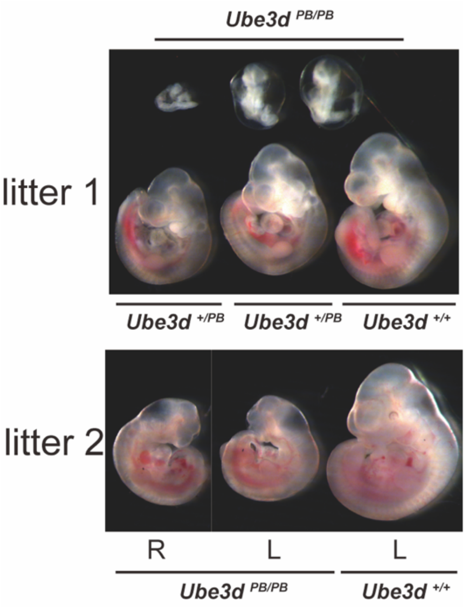

Fig. S2: PCR confirmation of the deletion of *Ube3d*.

(**a**) The schematic diagram of WT and KO *Ube3d*-CDS, along with the primers used in PCR confirmation, is provided. (**b**) Total mRNA was isolated from mice with different genotypes at E10.5, followed by reverse transcription to generate complementary DNA (cDNA), which was then used as a template in PCR.

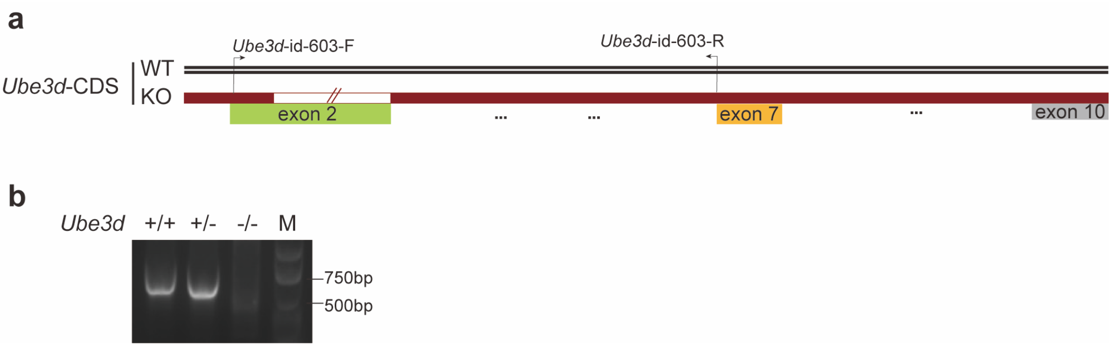

Fig. S3: The mRNA expression of Homeobox genes in E8.5 mice.

Total mRNA was extracted from fresh E8.5 embryos for qRT-PCR analysis. (**a**) The relative mRNA expression of *Ube3d* in different genotypes. (**b**) Only 5 out 11 Homeobox genes, which were downregulated in E10.5 embryos, plus *Nkx2-5* were detectable at E8.5. *Gapdh* was used as the internal control. In each experiment, at least 3 embryos per genotype were pooled together in each sample, and 3 independent experiments were conducted. The data are presented as mean ±SEM, and statistical analysis was performed using a two-side unpaired t-test (*p<0.05, ** p<0.01, *** p<0.001, **** p<0.0001. ns, not significant).

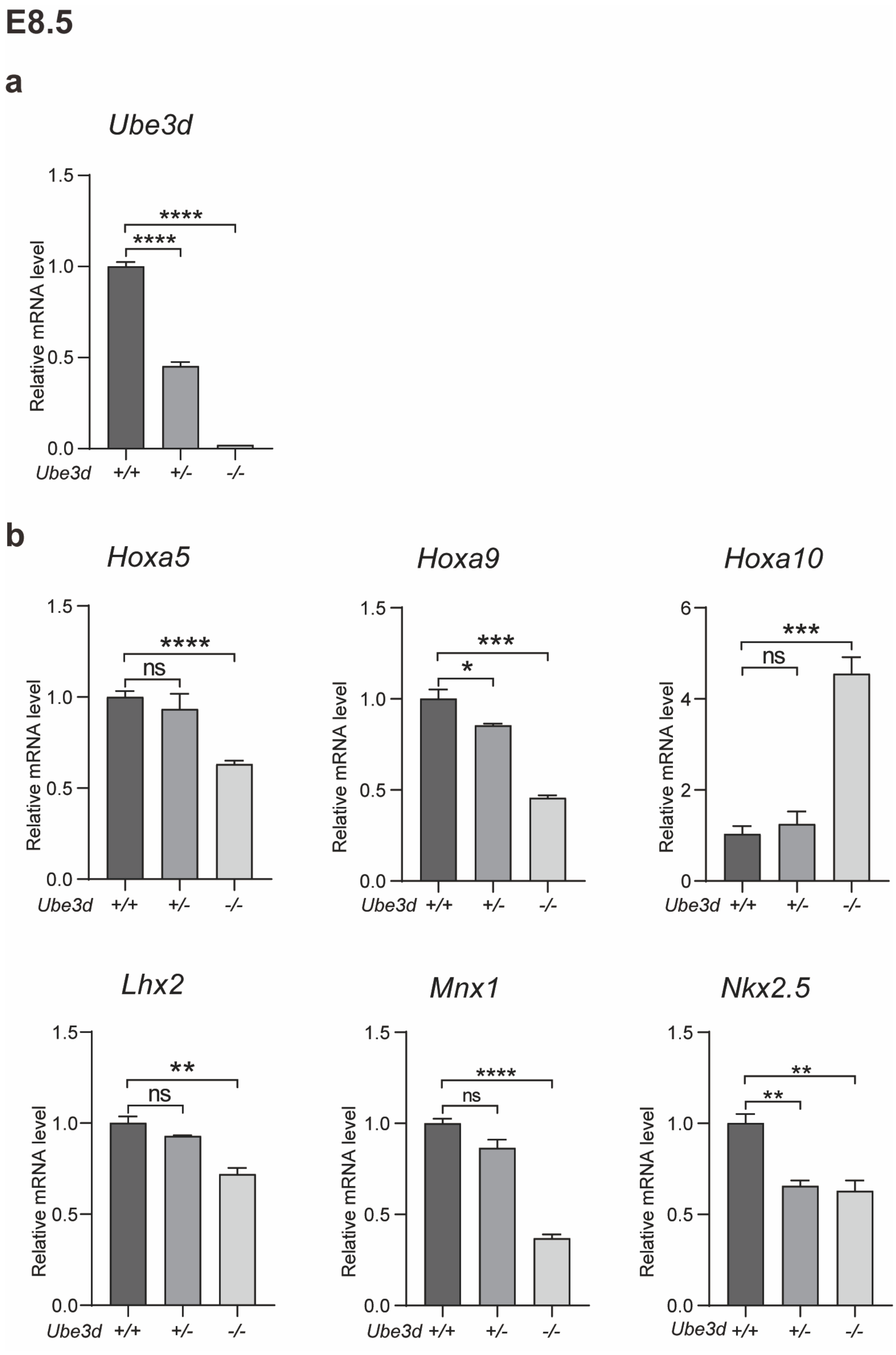
E8.5
a
Ube3d
Relative mRNA level
1.5
1.0
0.5
0.0
****
****
Ube3d
+/+
+/-
-/-
b
Hoxa5
Hoxa9
Hoxa10
Lhx2
Mnx1
Nkx2.5
ns
*
**
***
****
6
4
2
0

Fig. S4: Truncated UBE3D mutants interact with CPSF3 but not IgG.

Co-IP experiments were performed to examine the interaction between CPSF3 and two truncated UBE3D mutants. HEK 293T cells were co-transfected with CPSF3-Myc or Flag-CPSF3 with UBE3D or its mutants, followed by co-IP experiments using anti-Flag (**a**) or anti-Myc (**b**) antibodies, with IgG serving as a negative control.

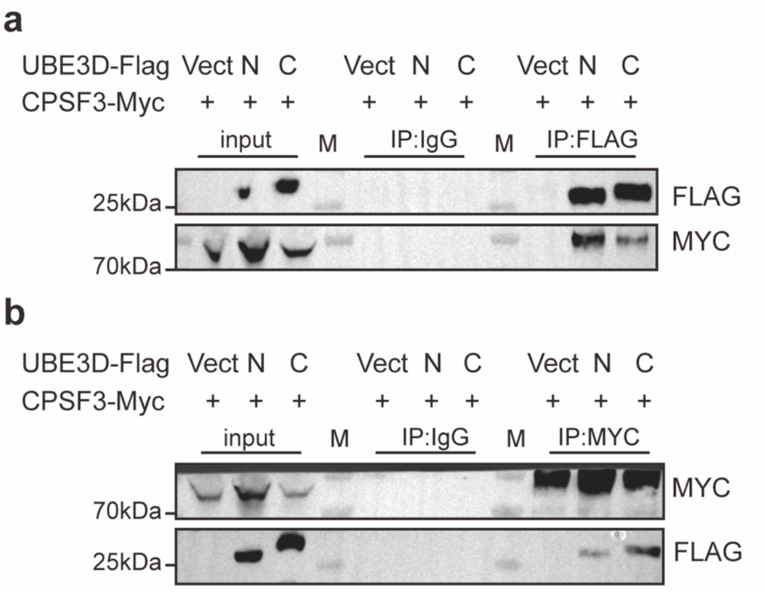

Supplement: Supplementary file 1 — Supplementary figures [file 41420_2025_2387_MOESM1_ESM.pdf]
