# Supplementary material for: Deficiency of UBE3D in mice leads to severe embryonic abnormalities and disrupts the mRNA of Homeobox genes via CPSF3
Source: Cell Death Discov. 2025 Mar 12;11:99. doi: 10.1038/s41420-025-02387-y (PMC11904178; doi:10.1038/s41420-025-02387-y)
Supplement: Supplementary file 2 — Supplementary tables [file 41420_2025_2387_MOESM2_ESM.pdf]

Table S1-710 DEGs of HOMO vs WT

gene_id	gene_name	HOMO	WT	log2FC	Pvalue	Qvalue	updown
ENSMUSG00000079502	1700101E01Rik	0.118093436	0.410355843	-1.796946723	0.001289255	0.028949803	DOWN
ENSMUSG00000052419	2610001J05Rik	3.179606753	6.659576785	-1.06658215	0.00000126	0.0000786	DOWN
ENSMUSG00000041789	2700046A07Rik	1.117056113	2.559927725	-1.196401421	0.00009	0.003322272	DOWN
ENSMUSG00000031125	3830403N18Rik	0	0.673759057	#NAME?	0.001268294	0.028559939	DOWN
ENSMUSG00000097004	4731419I09Rik	0.411691488	1.0433278	-1.34155698	0.001961088	0.040853884	DOWN
ENSMUSG00000097626	4921504A21Rik	0	0.222216335	#NAME?	0.002421289	0.047240738	DOWN
ENSMUSG00000059920	4930453N24Rik	2.134099826	6.760572205	-1.663517697	4.58E-13	6.73E-11	DOWN
ENSMUSG00000085488	4930557F10Rik	0	1.092986914	#NAME?	0.001268294	0.028559939	DOWN
ENSMUSG00000075585	6330403L08Rik	0.210258165	0.447830632	-1.090791386	0.001339387	0.029895483	DOWN
ENSMUSG00000102373	9530018H14Rik	0	0.856131782	#NAME?	0.0000154	0.000734425	DOWN
ENSMUSG00000048636	A730049H05Rik	0.084089065	0.498746407	-2.568316342	0.0000137	0.000668781	DOWN
ENSMUSG00000026842	Abl1	6.090815233	16.65972968	-1.451657746	2.24E-12	2.99E-10	DOWN
ENSMUSG00000037872	Ackr1	0.095093214	0.743412879	-2.966749385	0.0000253	0.001139147	DOWN
ENSMUSG00000059430	Actg2	0.352601506	1.212064451	-1.781355871	0.0000611	0.002390809	DOWN
ENSMUSG00000022449	Adamts20	0.984161765	2.09014237	-1.086633842	0.00000748	0.000392844	DOWN
ENSMUSG00000093738	AI606473	0.183330511	1.507549754	-3.039686804	7.41E-10	7.54E-08	DOWN
ENSMUSG00000038729	Akap2	0.885997319	5.942904477	-2.745793954	8.67E-31	5.25E-28	DOWN
ENSMUSG00000053279	Aldh1a1	2.035774345	8.84267794	-2.118905691	7.58E-15	1.29E-12	DOWN
ENSMUSG00000013584	Aldh1a2	8.999408189	32.98666807	-1.873981025	4.48E-17	9.64E-15	DOWN
ENSMUSG00000015134	Aldh1a3	0.393429651	4.96534714	-3.657716986	4.61E-35	3.88E-32	DOWN
ENSMUSG00000036602	Alx1	0.9780102	3.164148965	-1.693896105	3.59E-11	4.26E-09	DOWN
ENSMUSG00000014603	Alx3	5.327735628	15.83373766	-1.571407454	4.23E-11	4.99E-09	DOWN
ENSMUSG00000048534	Amica1	0.354368414	1.070082495	-1.5944001	0.000577021	0.015356044	DOWN
ENSMUSG00000079042	Apela	0.5070993	1.982417323	-1.966920511	5.7E-09	0.000000536	DOWN
ENSMUSG00000024411	Aqp4	0.171326878	0.480400957	-1.487487528	0.00149235	0.032576213	DOWN
ENSMUSG00000019987	Arg1	7.944174813	27.14363039	-1.772644415	3.01E-14	4.78E-12	DOWN
ENSMUSG00000017418	Arl5b	3.178391629	7.055417799	-1.150434618	3.09E-08	0.00000262	DOWN
ENSMUSG00000035277	Arx	0.405323237	1.040735028	-1.360458011	0.0000403	0.001684298	DOWN
ENSMUSG00000059995	Atxn7l3	7.048563836	22.11465732	-1.649601648	9.01E-15	1.51E-12	DOWN
ENSMUSG00000026432	Avpr1b	0	0.229051185	#NAME?	0.0000154	0.000734425	DOWN
ENSMUSG00000097428	AW047730	0.016297832	0.579794448	-5.152789545	2.01E-08	0.00000179	DOWN

ENSMUSG00000061132	Blnk	0.046466175	0.451006679	-3.278895993	9.2E-09	0.000000853	DOWN
ENSMUSG00000028487	Bnc2	0.22803094	0.525362511	-1.204083667	0.0000318	0.001385755	DOWN
ENSMUSG00000106775	C130093G08Rik	0.026086781	2.081043111	-6.317844084	1.73E-17	4.03E-15	DOWN
ENSMUSG00000058914	C1qtnf3	0.930771028	2.119070707	-1.186933517	0.0000629	0.002445635	DOWN
ENSMUSG00000029544	Cabp1	0.460577535	0.977105622	-1.085070475	0.000283396	0.008605813	DOWN
ENSMUSG00000028222	Calb1	0.149856738	0.496365671	-1.727819386	0.000218753	0.00695915	DOWN
ENSMUSG00000024617	Camk2a	0.482045257	1.207394932	-1.324657146	0.000000635	0.0000436	DOWN
ENSMUSG00000027861	Casq2	0.243780026	0.819608312	-1.749354693	0.0000625	0.002438018	DOWN
ENSMUSG00000049305	Ccdc71	2.305874921	8.257056391	-1.8403133	7.87E-16	1.51E-13	DOWN
ENSMUSG00000031962	Cdh15	0.877956164	1.981700819	-1.17451836	0.000426635	0.011809648	DOWN
ENSMUSG00000036510	Cdh8	0.151926813	0.3823013	-1.331333595	0.000378996	0.010782179	DOWN
ENSMUSG00000029646	Cdx2	0.308091027	3.267046147	-3.406558261	7.78E-17	1.64E-14	DOWN
ENSMUSG00000031326	Cdx4	0.059133611	3.922456977	-6.051635444	2.76E-26	1.24E-23	DOWN
ENSMUSG00000038192	Cer1	0.03339228	2.879811394	-6.430315901	1.28E-18	3.21E-16	DOWN
ENSMUSG00000031491	Chrna6	0.059968476	0.388006249	-2.693803681	0.001488068	0.032576213	DOWN
ENSMUSG00000027079	Clp1	3.685370784	8.691697058	-1.237828116	0.000000028	0.00000241	DOWN
ENSMUSG00000030092	Cntn6	0.049053122	0.196501174	-2.002121073	0.000819566	0.020382887	DOWN
ENSMUSG00000039714	Cplx3	0.08199775	0.508412501	-2.632343271	0.000421961	0.0117458	DOWN
ENSMUSG00000007888	Crlf1	1.89134025	10.01840306	-2.40517173	1.19E-18	3.03E-16	DOWN
ENSMUSG00000030905	Crym	4.619638088	10.45680889	-1.178590913	0.000000171	0.0000131	DOWN
ENSMUSG00000069045	Ddx3y	0.412928198	1.045806458	-1.340653038	0.00000286	0.000164238	DOWN
ENSMUSG00000048281	Dleu7	0.785587703	2.21216872	-1.493617169	0.000704966	0.017971073	DOWN
ENSMUSG00000090063	Dlx6os1	0.262861729	0.712151117	-1.437879301	0.0000117	0.000591535	DOWN
ENSMUSG00000028707	Dmbx1	0.789306123	1.788192998	-1.179845607	0.00000235	0.000138146	DOWN
ENSMUSG00000048138	Dmrt2	1.731276684	4.694341645	-1.439086534	0.000000323	0.0000233	DOWN
ENSMUSG00000042372	Dmrt3	0.631467711	1.492983331	-1.241417186	0.001088553	0.025625312	DOWN
ENSMUSG00000043753	Dmrta1	0.062040986	0.628940658	-3.34163039	3.7E-09	0.000000355	DOWN
ENSMUSG00000047143	Dmrta2	0.595970383	4.370422108	-2.874460085	4.76E-19	1.32E-16	DOWN
ENSMUSG00000085256	Dmrta2os	0	1.243727271	#NAME?	0.000000227	0.0000169	DOWN
ENSMUSG00000042523	Dnal1	0.335947722	1.542496733	-2.19895878	1.51E-15	2.82E-13	DOWN
ENSMUSG00000069049	Eif2s3y	0.718090973	1.644296275	-1.195231741	0.0000241	0.001092952	DOWN
ENSMUSG00000043969	Emx2	1.664229754	3.627699493	-1.124200335	0.00000548	0.000298875	DOWN

ENSMUSG00000032446	Eomes	0	0.298012596	#NAME?	7.43E-10	7.54E-08	DOWN
ENSMUSG00000026830	Ermn	0	0.172210018	#NAME?	0.002421289	0.047240738	DOWN
ENSMUSG00000000392	Fap	0.49065641	1.273526344	-1.376043791	0.00000388	0.000216062	DOWN
ENSMUSG00000029697	Fezf1	0.024105096	3.144286576	-7.027250746	1.43E-27	7.11E-25	DOWN
ENSMUSG00000021743	Fezf2	0	3.450597401	#NAME?	1.62E-28	8.48E-26	DOWN
ENSMUSG00000022101	Fgf17	1.686026248	6.382469753	-1.9204878	1.36E-10	1.52E-08	DOWN
ENSMUSG00000000183	Fgf6	0.149579947	0.537684348	-1.845842698	0.000148835	0.005094562	DOWN
ENSMUSG00000025219	Fgf8	0.737472449	1.621209685	-1.136409641	0.000247991	0.007724411	DOWN
ENSMUSG00000001334	Fndc5	1.281549074	4.372707797	-1.77063822	4.83E-10	5.06E-08	DOWN
ENSMUSG00000044518	Foxe3	1.071225338	3.104718606	-1.535200526	0.000764908	0.019270902	DOWN
ENSMUSG00000070880	Gad1	0.12515183	0.35582404	-1.507484596	0.000197693	0.006447996	DOWN
ENSMUSG00000081169	Gm12551	0.159888812	1.897005318	-3.568582825	0.00005	0.002005072	DOWN
ENSMUSG00000087333	Gm13652	0.119081773	0.587107056	-2.301670992	0.000064	0.002481592	DOWN
ENSMUSG00000090639	Gm20425	0	0.785600834	#NAME?	8.24E-11	9.45E-09	DOWN
ENSMUSG00000092595	Gm20427	1.410822678	2.840084555	-1.009397211	0.000186934	0.00620366	DOWN
ENSMUSG00000093593	Gm20683	0.416118278	1.884725943	-2.17928919	2.07E-08	0.00000183	DOWN
ENSMUSG00000095134	Gm21857	1.348843175	20.16443838	-3.902018698	3.21E-36	2.96E-33	DOWN
ENSMUSG00000097239	Gm27029	1.153248852	3.316551003	-1.523979855	0.00000124	0.0000776	DOWN
ENSMUSG00000098950	Gm28036	0.540345123	4.675993978	-3.113320003	9.5E-33	6.35E-30	DOWN
ENSMUSG00000098650	Gm28048	0.021146823	1.664167379	-6.298215809	2.68E-17	6.12E-15	DOWN
ENSMUSG00000099521	Gm28309	0.559064055	2.713029804	-2.278819401	0.000538084	0.014418863	DOWN
ENSMUSG00000099519	Gm29253	0.066583236	0.646219229	-3.278792792	7.95E-10	8.03E-08	DOWN
ENSMUSG00000101678	Gm29609	0.228214853	1.599711814	-2.809347436	8.66E-12	1.08E-09	DOWN
ENSMUSG00000103865	Gm37416	5.841209862	12.59880047	-1.108947258	0.002320767	0.046257752	DOWN
ENSMUSG00000097156	Gm3764	0.120432212	0.970243643	-3.010125757	2.34E-15	4.16E-13	DOWN
ENSMUSG00000103766	Gm38392	0	0.392220185	#NAME?	0.002421289	0.047240738	DOWN
ENSMUSG00000105204	Gm43738	2.731635821	6.53319937	-1.258024509	0.000000667	0.0000453	DOWN
ENSMUSG00000078706	Gm53	0.054708338	2.183035799	-5.318431261	1.17E-31	7.55E-29	DOWN
ENSMUSG00000074634	Gm7120	0.419243332	1.511389393	-1.850015658	2.68E-08	0.00000232	DOWN
ENSMUSG00000056380	Gpr50	1.880651577	4.888739224	-1.378229861	2.84E-08	0.00000243	DOWN
ENSMUSG00000050105	Grrp1	3.620024577	8.081828597	-1.158682262	0.00000643	0.00033962	DOWN
ENSMUSG00000052305	Hbb-bs	2.04205311	5.920632868	-1.535731008	0.000000154	0.0000119	DOWN

ENSMUSG00000023781	Hes7	0.160435451	2.59535672	-4.015867974	1.27E-14	2.08E-12	DOWN
ENSMUSG00000025877	Hk3	0.114102181	0.440915812	-1.950176851	0.0000509	0.002036557	DOWN
ENSMUSG00000003038	Hmgn2	12.60740816	25.66445368	-1.025499834	0.000000509	0.0000361	DOWN
ENSMUSG00000086903	Hotair	0	1.154636582	#NAME?	1.42E-10	1.58E-08	DOWN
ENSMUSG00000055408	Hottip	0	0.187620805	#NAME?	0.0000996	0.003593996	DOWN
ENSMUSG00000000938	Hoxa10	1.340352901	4.892361926	-1.867918238	9.97E-15	1.65E-12	DOWN
ENSMUSG00000038210	Hoxa11	1.747387833	5.224998052	-1.58023065	1.81E-10	0.00000002	DOWN
ENSMUSG00000086427	Hoxa11os	0.437659056	3.20452138	-2.872229567	4.24E-25	1.68E-22	DOWN
ENSMUSG00000038203	Hoxa13	0.168658613	0.67404208	-1.998732667	0.00000191	0.000113586	DOWN
ENSMUSG00000038253	Hoxa5	4.020328873	14.24090444	-1.824655347	1.6E-13	2.44E-11	DOWN
ENSMUSG00000043219	Hoxa6	1.394714072	5.228608265	-1.906457598	0.0000015	0.0000919	DOWN
ENSMUSG00000038236	Hoxa7	0.982787271	2.39034987	-1.28227072	0.00000139	0.0000853	DOWN
ENSMUSG00000038227	Hoxa9	1.038403178	3.539041267	-1.768991881	7.88E-13	1.09E-10	DOWN
ENSMUSG00000085696	Hoxaas3	0.345026917	2.197107667	-2.670824746	1.26E-13	1.93E-11	DOWN
ENSMUSG00000049604	Hoxb13	0	1.305342727	#NAME?	6.97E-13	9.75E-11	DOWN
ENSMUSG00000038700	Hoxb5	1.624487603	4.988208684	-1.618537088	6.14E-11	7.13E-09	DOWN
ENSMUSG00000085645	Hoxb5os	1.07781577	24.65809841	-4.515879041	8.01E-37	7.76E-34	DOWN
ENSMUSG00000000690	Hoxb6	1.325276746	7.012728506	-2.40368222	3.31E-19	9.57E-17	DOWN
ENSMUSG00000038721	Hoxb7	1.397881349	5.690908462	-2.025417063	8.08E-11	9.32E-09	DOWN
ENSMUSG00000056648	Hoxb8	0.797504525	5.188471429	-2.70174496	1.07E-22	3.82E-20	DOWN
ENSMUSG00000020875	Hoxb9	1.83989744	33.84021058	-4.201041292	1.09E-58	3.02E-55	DOWN
ENSMUSG00000022484	Hoxc10	0.152031289	21.05248567	-7.113478502	5.12E-69	2.48E-65	DOWN
ENSMUSG00000001656	Hoxc11	0	5.141798157	#NAME?	2.43E-34	1.96E-31	DOWN
ENSMUSG00000050328	Hoxc12	0	5.16099294	#NAME?	4.85E-21	1.65E-18	DOWN
ENSMUSG00000001655	Hoxc13	0.094359053	2.365611724	-4.647908553	7.44E-19	2.03E-16	DOWN
ENSMUSG00000001661	Hoxc6	1.617240288	6.914032784	-2.095993395	2.07E-15	3.72E-13	DOWN
ENSMUSG00000001657	Hoxc8	0.510809671	1.239450099	-1.278842444	0.000207922	0.00671381	DOWN
ENSMUSG00000036139	Hoxc9	0.135075468	2.268419636	-4.069849962	8.67E-19	2.3E-16	DOWN
ENSMUSG00000042448	Hoxd1	0.841716974	3.597319861	-2.095515329	2.04E-09	0.000000197	DOWN
ENSMUSG00000050368	Hoxd10	1.85394914	10.30693991	-2.474942494	8.48E-19	2.28E-16	DOWN
ENSMUSG00000042499	Hoxd11	1.348135209	6.178922534	-2.19639009	5.28E-18	1.28E-15	DOWN
ENSMUSG00000001823	Hoxd12	1.351531863	8.127630417	-2.588239276	1.27E-20	4.18E-18	DOWN

ENSMUSG00000001819	Hoxd13	1.589700806	9.328235935	-2.552849014	5.58E-21	1.86E-18	DOWN
ENSMUSG00000052371	Hoxd3os1	0.033471533	1.155253331	-5.109130796	2.06E-14	3.3E-12	DOWN
ENSMUSG00000027102	Hoxd8	0.492628667	1.125758151	-1.192324435	0.000368005	0.010562574	DOWN
ENSMUSG00000025491	Ifitm1	7.784157103	24.60829276	-1.660531839	3.11E-12	4.1E-10	DOWN
ENSMUSG00000003477	Inmt	0.116341497	0.846802389	-2.863659562	0.0000648	0.002494734	DOWN
ENSMUSG00000054667	Irs4	0.866926427	2.556118557	-1.559973285	3.1E-09	0.000000299	DOWN
ENSMUSG00000074766	Ism1	0.762307281	1.644191547	-1.108933821	0.000122294	0.00430785	DOWN
ENSMUSG00000056673	Kdm5d	0.181461715	1.054223122	-2.538443136	3.8E-20	1.19E-17	DOWN
ENSMUSG00000000247	Lhx2	2.311814867	7.048240717	-1.608237327	1.42E-12	1.95E-10	DOWN
ENSMUSG00000026890	Lhx6	0.300023323	1.522169714	-2.342982659	5.97E-15	1.03E-12	DOWN
ENSMUSG00000096225	Lhx8	0.690870347	3.963034136	-2.520118497	9.04E-18	2.16E-15	DOWN
ENSMUSG00000090077	Lime1	0.616564971	1.274435077	-1.047533047	0.001054355	0.024880727	DOWN
ENSMUSG00000001493	Meox1	7.399237978	31.6652609	-2.097452361	2.63E-20	8.36E-18	DOWN
ENSMUSG00000036144	Meox2	2.312048699	5.358125728	-1.212556648	0.00000569	0.000306997	DOWN
ENSMUSG00000030544	Mesp1	0	0.638257599	#NAME?	0.000352487	0.010177462	DOWN
ENSMUSG00000001566	Mnx1	0.231485232	0.851707366	-1.879437671	0.0000129	0.000643992	DOWN
ENSMUSG00000047002	Msgn1	0	10.59899087	#NAME?	3.27E-23	1.22E-20	DOWN
ENSMUSG00000020908	Myh3	1.343041744	3.084858344	-1.199700096	0.000000787	0.0000514	DOWN
ENSMUSG00000030672	Mylpf	3.863227179	8.439238349	-1.127306281	0.0000108	0.000551518	DOWN
ENSMUSG00000031461	Myom2	0	0.073550711	#NAME?	0.001268294	0.028559939	DOWN
ENSMUSG00000029413	Naaa	0.82427838	1.777849985	-1.108930034	0.0000408	0.001698699	DOWN
ENSMUSG00000026393	Nek7	0.980350261	2.95312651	-1.590873967	4.54E-12	5.86E-10	DOWN
ENSMUSG00000017837	Nkiras2	6.526916233	14.21480285	-1.122920661	0.000000118	0.00000927	DOWN
ENSMUSG00000048528	Nkx1-2	0.213417222	1.76445224	-3.047471873	6.02E-13	8.7E-11	DOWN
ENSMUSG00000001496	Nkx2-1	0.341666921	3.161022011	-3.209728598	1.82E-19	5.5E-17	DOWN
ENSMUSG00000054160	Nkx2-4	0.067292335	1.016125165	-3.916492132	0.00000215	0.000126467	DOWN
ENSMUSG00000022061	Nkx3-1	0.781229851	4.09496947	-2.390033715	2.06E-15	3.72E-13	DOWN
ENSMUSG00000068302	Noto	0	1.302980116	#NAME?	6.97E-13	9.75E-11	DOWN
ENSMUSG00000019803	Nr2e1	0	6.979152994	#NAME?	1.18E-63	4.58E-60	DOWN
ENSMUSG00000020902	Ntn1	4.917738669	25.21797273	-2.358385326	8.48E-27	3.91E-24	DOWN
ENSMUSG00000009941	Nxf2	0.018792613	0.263358718	-3.808791601	0.002503814	0.048171686	DOWN
ENSMUSG00000046160	Olig1	0.161099402	1.650430231	-3.356819112	2.18E-10	2.36E-08	DOWN

ENSMUSG00000039830	Olig2	0.928958071	8.065809605	-3.118133965	1.21E-25	5.08E-23	DOWN
ENSMUSG00000005917	Otx1	0.876889359	3.149730495	-1.844761662	9.71E-14	1.5E-11	DOWN
ENSMUSG00000021848	Otx2	2.919010908	7.003405322	-1.262576984	1.76E-08	0.00000157	DOWN
ENSMUSG00000037034	Pax1	1.787499669	5.032037413	-1.493199673	7.3E-10	7.48E-08	DOWN
ENSMUSG00000046387	Pcdhb17	0	0.241796169	#NAME?	0.00000835	0.000433769	DOWN
ENSMUSG00000102440	Pcdhga9	0.613310165	17.96292325	-4.872261478	1.38E-74	8.93E-71	DOWN
ENSMUSG00000103037	Pcdhgb1	0.51388013	3.736976064	-2.862367549	3.86E-27	1.87E-24	DOWN
ENSMUSG00000005615	Pcyt1a	2.757830703	7.174411804	-1.379326661	9.71E-11	0.000000011	DOWN
ENSMUSG00000024901	Peli3	0.45668681	1.80234792	-1.980600505	4.65E-11	5.43E-09	DOWN
ENSMUSG00000067825	Pex26	0.546451593	1.097828042	-1.006486488	0.0000781	0.002948762	DOWN
ENSMUSG00000027088	Phospho2	1.080790068	2.533460003	-1.229022731	0.000000623	0.000043	DOWN
ENSMUSG00000021506	Pitx1	3.132132095	13.8346972	-2.143074104	2.83E-19	8.31E-17	DOWN
ENSMUSG00000034403	Pja1	4.777330891	10.20371331	-1.09481756	0.000000139	0.0000108	DOWN
ENSMUSG00000035456	Prdm8	0.879704871	2.69483564	-1.61510578	7.94E-09	0.000000739	DOWN
ENSMUSG00000024518	Rax	0.069550196	4.088320111	-5.877309765	7.06E-24	2.74E-21	DOWN
ENSMUSG00000046667	Rbm12b1	0.073284522	0.553208053	-2.916241719	8.26E-10	8.29E-08	DOWN
ENSMUSG00000028896	Rcc1	13.82513696	28.82052724	-1.059802956	0.000000257	0.0000189	DOWN
ENSMUSG00000091957	Rps2-ps10	0.661052114	8.792519999	-3.733440794	1.12E-28	6E-26	DOWN
ENSMUSG00000060735	Rxfp3	0.271571929	0.702873155	-1.371929992	0.001489598	0.032576213	DOWN
ENSMUSG00000021963	Sap18	0.475056069	2.55065053	-2.424695543	1.7E-16	3.5E-14	DOWN
ENSMUSG00000046480	Scn4b	0.505269104	1.381256329	-1.450857205	0.00000775	0.000405916	DOWN
ENSMUSG00000007279	Scube2	1.155169377	2.461407494	-1.091379118	0.000017	0.000801728	DOWN
ENSMUSG00000040364	Sec1	0.400008733	1.497132892	-1.904098885	0.00000941	0.000485052	DOWN
ENSMUSG00000019913	Sim1	0.157847722	0.451091019	-1.514885121	0.000366334	0.010545839	DOWN
ENSMUSG00000062713	Sim2	0.72756889	1.466916093	-1.011630591	0.002294377	0.04582605	DOWN
ENSMUSG00000038805	Six3	0.045463467	1.316862475	-4.856253156	4.75E-30	2.71E-27	DOWN
ENSMUSG00000093460	Six3os1	0.026608089	0.935485696	-5.135778789	6.36E-42	6.85E-39	DOWN
ENSMUSG00000021099	Six6	0.028954671	1.311645143	-5.501437527	2.33E-19	6.95E-17	DOWN
ENSMUSG00000020261	Slc36a1	1.162531177	3.061774941	-1.397098833	1.01E-08	0.000000927	DOWN
ENSMUSG00000034224	Slc38a8	0.025378067	0.84853998	-5.063328554	8E-14	1.25E-11	DOWN
ENSMUSG00000036790	Slitrk2	0.280501323	0.575915143	-1.037848682	0.000841501	0.020821497	DOWN
ENSMUSG00000097769	Snhg4	1.382342107	2.900612912	-1.069243077	0.0000197	0.000910114	DOWN

ENSMUSG00000075304	Sp5	1.713776283	4.680090002	-1.449357482	0.000000732	0.000049	DOWN
ENSMUSG00000028327	Stra6l	0.039901717	0.279702363	-2.809369706	2.89E-08	0.00000246	DOWN
ENSMUSG00000017548	Suz12	5.842067993	11.71815594	-1.0041945	0.00000159	0.0000961	DOWN
ENSMUSG00000053025	Sv2b	0.06700955	0.234861191	-1.809369706	0.0000443	0.00182552	DOWN
ENSMUSG00000062327	T	0.254589715	8.110116939	-4.993476674	4.97E-45	6.42E-42	DOWN
ENSMUSG00000000094	Tbx4	0.969370592	4.125375256	-2.089405136	9.21E-16	1.73E-13	DOWN
ENSMUSG00000030699	Tbx6	0.280054665	4.118421322	-3.87831106	6.8E-22	2.35E-19	DOWN
ENSMUSG00000068079	Tcf15	4.26473952	15.77778243	-1.887364916	6.59E-12	8.4E-10	DOWN
ENSMUSG00000055320	Tead1	0.982725572	2.486551094	-1.339285573	9.84E-10	9.78E-08	DOWN
ENSMUSG00000025013	Tll2	0.067446305	0.490914193	-2.863659562	0.0000648	0.002494734	DOWN
ENSMUSG00000009214	Tmem8c	0.052500804	0.566272808	-3.431085832	0.000143701	0.004927539	DOWN
ENSMUSG00000017300	Tnnc2	1.886509787	6.259990017	-1.730440772	0.0000026	0.000150831	DOWN
ENSMUSG00000054517	Trim65	0.230621833	0.638352137	-1.468823387	0.000048	0.001936674	DOWN
ENSMUSG00000030523	Trpm1	0.057100348	0.408656707	-2.839317978	5.99E-16	1.17E-13	DOWN
ENSMUSG00000046269	Usp27x	0.270418367	0.649912862	-1.265053157	0.000335547	0.009940204	DOWN
ENSMUSG00000056423	Uts2b	0.208561022	1.874276509	-3.167792346	0.0000468	0.001911253	DOWN
ENSMUSG00000068457	Uty	0.06100906	0.472159796	-2.952179798	1.02E-14	1.68E-12	DOWN
ENSMUSG00000006270	Vax1	0	1.499699331	#NAME?	3.99E-09	0.000000381	DOWN
ENSMUSG00000085794	Vax2os	0.017343385	0.140292114	-3.015976487	0.002009464	0.041593325	DOWN
ENSMUSG00000073643	Wdfy1	1.794178634	3.915862508	-1.126006573	0.000000743	0.0000496	DOWN
ENSMUSG00000029671	Wnt16	0.22960253	0.866630167	-1.916277915	0.000000916	0.0000587	DOWN
ENSMUSG00000036961	Wnt8b	0.154752772	4.72838078	-4.933309061	6.59E-36	5.8E-33	DOWN
ENSMUSG00000035067	Xkr6	0.199847845	1.151505174	-2.526546974	1.6E-15	2.92E-13	DOWN
ENSMUSG00000091736	Yy2	0.161972858	0.938887698	-2.535200526	0.00000119	0.0000745	DOWN
ENSMUSG00000029587	Zfp12	1.147976864	4.142241833	-1.851318217	2.67E-16	5.33E-14	DOWN
ENSMUSG00000098022	Zfp82	0.25469684	0.712116454	-1.483332127	0.000000872	0.0000565	DOWN
ENSMUSG00000026621	01-Mar	0.292297843	0.042983526	2.765583448	0.000556462	0.014870211	UP
ENSMUSG00000056329	1810010K12Rik	0.389739946	0.049445767	2.978592873	0.002009464	0.041593325	UP
ENSMUSG00000085562	2610028E06Rik	0.381348133	0.120955188	1.656635991	0.000400542	0.011262838	UP
ENSMUSG00000026227	2810459M11Rik	0.528250959	0.130209759	2.020385906	0.000608709	0.016088842	UP
ENSMUSG00000085582	3110099E03Rik	0.307418478	0.043240853	2.829736997	0.000339552	0.010012907	UP
ENSMUSG00000021098	4930447C04Rik	0.435596722	0.13918475	1.645991945	0.000289093	0.008724113	UP

ENSMUSG00000102344	9430053O09Rik	1.264837855	0.511528218	1.30606672	0.002063944	0.042403874	UP
ENSMUSG00000079144	A130010J15Rik	1.548662617	0.722534031	1.099885437	0.0000387	0.0016351	UP
ENSMUSG00000052595	A1cf	0.288114112	0	Inf	0.0000154	0.000734425	UP
ENSMUSG00000026782	Abi2	4.353485218	1.176973193	1.887089363	1.83E-16	3.73E-14	UP
ENSMUSG00000036545	Adamts2	1.3711987	0.661543501	1.051529715	0.000150537	0.005134682	UP
ENSMUSG00000031994	Adamts8	0.972968796	0.293800856	1.727554936	0.00000261	0.000151062	UP
ENSMUSG00000024256	Adcyap1	2.001649775	0.839471221	1.253636798	0.001493118	0.032576213	UP
ENSMUSG00000025475	Adgra1	0.817314827	0.225254099	1.859338546	0.0000021	0.000124302	UP
ENSMUSG00000028782	Adgrb2	2.212181273	1.020006627	1.116891084	0.00000801	0.000418495	UP
ENSMUSG00000037605	Adgrl3	0.71450909	0.342464715	1.060997013	0.00002	0.000916856	UP
ENSMUSG00000030790	Adm	1.634282591	0.500460272	1.707330015	0.0000173	0.000814358	UP
ENSMUSG00000033717	Adra2a	2.107480526	0.938760895	1.166689649	0.000191087	0.006306859	UP
ENSMUSG00000039313	AF529169	0.319266043	0.102014001	1.645991945	0.000289093	0.008724113	UP
ENSMUSG00000029369	Afm	0.580483261	0.073645108	2.978592873	0.00000616	0.000327417	UP
ENSMUSG00000054932	Afp	75.39375057	1.114191721	6.080375537	9.02E-106	8.74E-102	UP
ENSMUSG00000097023	AI854517	1.212911488	0.572749996	1.082496826	0.000269813	0.008271137	UP
ENSMUSG00000041323	Ak7	0.506717428	0.040477427	3.645991945	0.0000103	0.000527009	UP
ENSMUSG00000021057	Akap5	0.886108748	0.391642967	1.177944711	0.0000199	0.000916703	UP
ENSMUSG00000021211	Akr1c12	0.64242932	0.049484855	3.698478785	0.002503814	0.048171686	UP
ENSMUSG00000071551	Akr1c19	0.963436066	0.155846493	2.62806324	0.0000216	0.000986132	UP
ENSMUSG00000029368	Alb	13.02275124	0.219814699	5.888602599	6.83E-56	1.65E-52	UP
ENSMUSG00000022636	Alcam	7.672638826	3.331157246	1.203699387	0.000000115	0.00000904	UP
ENSMUSG00000075296	Aldh3b2	1.464060724	0.380092685	1.945552227	0.0000426	0.001758592	UP
ENSMUSG00000017390	Aldoc	1.38158955	0.665962287	1.05281669	0.000810289	0.020282341	UP
ENSMUSG00000039754	Alkbh4	3.321252397	1.33166966	1.318491119	0.000000559	0.0000389	UP
ENSMUSG00000032845	Alpk2	1.066772129	0.50560813	1.07716047	0.000373156	0.010672422	UP
ENSMUSG00000028356	Ambp	20.03221866	0.342900227	5.868389545	2.08E-55	4.48E-52	UP
ENSMUSG00000029994	Anxa4	1.452150643	0.629728478	1.205389308	0.0000303	0.001331869	UP
ENSMUSG00000021950	Anxa8	1.375407639	0.412011901	1.739101349	0.000159196	0.005382659	UP
ENSMUSG00000019326	Aoc3	0.270952932	0.044953926	2.591523246	0.000264517	0.00814747	UP
ENSMUSG00000032083	Apoa1	7.280411097	0.126631844	5.84530777	9.05E-30	5.01E-27	UP
ENSMUSG00000005681	Apoa2	5.657652561	0.610120287	3.213037976	7.28E-17	1.55E-14	UP

ENSMUSG00000020609	Apob	0.736193596	0.085065256	3.113443304	5.85E-20	1.8E-17	UP
ENSMUSG00000040564	Apoc1	2.272640378	0.204236024	3.476060146	0.00005	0.002005072	UP
ENSMUSG00000024391	Apom	8.079646527	0.556155057	3.860733108	4.12E-19	1.17E-16	UP
ENSMUSG00000037999	Arap2	0.416142127	0.140793455	1.563496076	0.0000115	0.000585541	UP
ENSMUSG00000019852	Arfgef3	0.416842958	0.167627296	1.314246873	0.001920569	0.040096023	UP
ENSMUSG00000037509	Arhgef4	0.77407543	0.223203268	1.79411601	4.8E-09	0.000000454	UP
ENSMUSG00000038204	Asb10	0.289828473	0.017364311	4.061002255	0.000252789	0.007861218	UP
ENSMUSG00000021200	Asb2	5.001858377	1.905466391	1.392320048	4.93E-08	0.00000407	UP
ENSMUSG00000040963	Asgr2	0.320834998	0	Inf	0.000352487	0.010177462	UP
ENSMUSG00000073043	Atoh1	8.83984628	1.4461957	2.61175849	4.64E-19	1.3E-16	UP
ENSMUSG00000046876	Atxn1	0.547659768	0.272970041	1.00453728	0.001833946	0.038704649	UP
ENSMUSG00000054753	AU018091	0.758295431	0.258966	1.549997336	0.00035419	0.01021143	UP
ENSMUSG00000069920	B3gnt9	1.039271406	0.502012501	1.049777268	0.002146184	0.043539448	UP
ENSMUSG00000026805	Barhl1	1.0391461	0.241151819	2.107384906	2.07E-10	2.25E-08	UP
ENSMUSG00000041674	BC006965	0.251692362	0.005322083	5.563526586	8.6E-12	1.08E-09	UP
ENSMUSG00000025105	Bnc1	5.539025353	2.690732868	1.041632973	0.000026	0.001162584	UP
ENSMUSG00000049630	C1ql3	1.648696146	0.493897112	1.739043098	0.0000212	0.000967053	UP
ENSMUSG00000024164	C3	1.13437614	0.066125009	4.100559278	4.15E-17	9.03E-15	UP
ENSMUSG00000035031	C8a	0.086364449	0	Inf	0.002421289	0.047240738	UP
ENSMUSG00000003657	Calb2	2.476167897	0.656625959	1.914965448	0.0000243	0.001102164	UP
ENSMUSG00000000805	Car4	6.606229834	2.075265266	1.670531405	5.08E-10	5.29E-08	UP
ENSMUSG00000007122	Casq1	7.73168301	3.782224456	1.031547507	0.0000409	0.001698699	UP
ENSMUSG00000028977	Casz1	2.087980334	0.624505682	1.74132152	4.28E-12	5.56E-10	UP
ENSMUSG00000034031	Ccdc182	5.833696851	1.871762991	1.640012651	0.00000136	0.0000838	UP
ENSMUSG00000026676	Ccdc3	2.608877091	0.984716363	1.405648841	0.0000304	0.001331869	UP
ENSMUSG00000039977	Ccdc67	0.366689975	0.098860299	1.891097646	0.000610301	0.016108959	UP
ENSMUSG00000042417	Ccno	2.011235953	0.100103958	4.328511428	1.13E-11	1.39E-09	UP
ENSMUSG00000037944	Ccr7	0.440183616	0.031647291	3.797951536	0.001410364	0.031227883	UP
ENSMUSG00000026012	Cd28	0.111118232	0.009217839	3.591523246	0.002503814	0.048171686	UP
ENSMUSG00000034652	Cd300a	0.455841811	0.143876671	1.663700578	0.001570614	0.034036988	UP
ENSMUSG00000026399	Cd55	1.24001056	0.53289021	1.218442174	0.000135707	0.004694962	UP
ENSMUSG00000078926	Cdc20b	0.729611728	0	Inf	0.000000411	0.0000294	UP

ENSMUSG00000031841	Cdh13	0.777609035	0.381180507	1.028570637	0.000497711	0.013505129	UP
ENSMUSG00000047216	Cdh19	0.330042694	0.071183013	2.213047763	0.001177692	0.027194998	UP
ENSMUSG00000023067	Cdkn1a	12.68962096	5.620552597	1.174865092	0.000000671	0.0000455	UP
ENSMUSG00000094626	Cecr6	1.35908912	0.578555702	1.232112291	0.000258465	0.008011996	UP
ENSMUSG00000075256	Cerkl	0.179573157	0.024206629	2.891097646	0.000206996	0.006695046	UP
ENSMUSG00000071550	Cfap44	0.281705528	0.040894857	2.784196559	0.000000467	0.0000333	UP
ENSMUSG00000090231	Cfb	0.616751158	0	Inf	4.16E-13	6.15E-11	UP
ENSMUSG00000058952	Cfi	0.8292462	0	Inf	1.3E-09	0.000000128	UP
ENSMUSG00000027536	Chmp4c	0.330864819	0.127004881	1.381357958	0.000949785	0.022915474	UP
ENSMUSG00000029205	Chrna9	0.126268319	0	Inf	0.002421289	0.047240738	UP
ENSMUSG00000022512	Cldn1	2.806724453	1.253161962	1.163314551	0.000140106	0.004821351	UP
ENSMUSG00000043850	Clrn1	0.268949718	0.050006664	2.427144217	0.000200837	0.006539513	UP
ENSMUSG00000031789	Cngb1	0.085125603	0.010200153	3.061002255	0.001187534	0.027292141	UP
ENSMUSG00000030075	Cntn3	0.277341018	0.095931831	1.531579498	0.000674911	0.017434309	UP
ENSMUSG00000020953	Coch	1.450662756	0.357577102	2.020385906	0.00000289	0.000164975	UP
ENSMUSG00000040690	Col16a1	2.783685667	1.194204421	1.220946497	0.000000918	0.0000587	UP
ENSMUSG00000001506	Col1a1	140.2548931	62.42848688	1.1677747	4.51E-08	0.00000375	UP
ENSMUSG00000031273	Col4a6	5.703106642	2.489083025	1.196133657	0.000000293	0.0000214	UP
ENSMUSG00000001119	Col6a1	10.48186834	4.753728609	1.140764445	0.000000943	0.0000601	UP
ENSMUSG00000020241	Col6a2	5.825381498	2.892651262	1.009960131	0.0000124	0.000624821	UP
ENSMUSG00000048126	Col6a3	3.60540633	1.566258974	1.202839085	0.000000222	0.0000167	UP
ENSMUSG00000028626	Col9a2	6.094343442	2.247980298	1.438841413	2.09E-08	0.00000184	UP
ENSMUSG00000027570	Col9a3	1.362757501	0.572721292	1.250623716	0.0000825	0.003085785	UP
ENSMUSG00000038591	Colec10	0.936456073	0.068234512	3.778637797	6.97E-12	8.82E-10	UP
ENSMUSG00000036655	Colec11	2.812117083	0.633678077	2.149834653	0.00000171	0.000102544	UP
ENSMUSG00000005220	Corin	3.086761957	1.208308699	1.353105148	8.49E-08	0.00000674	UP
ENSMUSG00000009876	Cox4i2	4.691689536	2.206873902	1.08810335	0.001711227	0.036593065	UP
ENSMUSG00000030785	Cox6a2	9.560773014	3.958516222	1.272167505	0.000194734	0.006405402	UP
ENSMUSG00000078937	Cpt1b	3.315759299	1.592720847	1.057845851	0.000135371	0.004692684	UP
ENSMUSG00000026616	Cr2	0.105976266	0.005714134	4.213062445	0.0000808	0.003033921	UP
ENSMUSG00000063681	Crb1	0.138934559	0.033295219	2.061018569	0.000104191	0.003731229	UP
ENSMUSG00000027230	Creb3l1	10.01279781	4.280759325	1.225906522	0.000000911	0.0000586	UP

ENSMUSG00000023968	Crip3	0.217466709	0	Inf	0.000352487	0.010177462	UP
ENSMUSG00000017776	Crk	21.1364736	8.536674068	1.307988695	1.82E-09	0.000000178	UP
ENSMUSG00000030470	Csrp3	34.78076837	14.79752582	1.23293383	0.000000305	0.0000221	UP
ENSMUSG00000028015	Ctso	0.982835618	0.45424891	1.113467091	0.001118277	0.026156108	UP
ENSMUSG00000024987	Cyp26a1	15.81328584	6.002272866	1.397556365	1.74E-08	0.00000157	UP
ENSMUSG00000063415	Cyp26b1	12.05940728	2.24679296	2.424219908	1.25E-25	5.15E-23	UP
ENSMUSG00000062432	Cyp26c1	21.23200968	2.149985796	3.3038419	7.79E-33	5.39E-30	UP
ENSMUSG00000026832	Cytip	0.077727495	0	Inf	0.000667207	0.017281373	UP
ENSMUSG00000097466	D430036J16Rik	0.258983832	0.050779429	2.350545948	0.001695242	0.036291295	UP
ENSMUSG00000025592	Dach2	0.774797165	0.331344757	1.225485582	0.00004	0.001675427	UP
ENSMUSG00000060534	Dcc	2.838803242	1.289692212	1.138256054	0.00000334	0.000188052	UP
ENSMUSG00000102692	Dchs2	0.651374479	0.24382757	1.417625932	0.0000251	0.001132015	UP
ENSMUSG00000042388	Dlgap3	1.724847871	0.831339976	1.052958632	0.000281389	0.008558291	UP
ENSMUSG00000028610	Dmrtb1	1.47046654	0.67686262	1.119339007	0.002579339	0.049330812	UP
ENSMUSG00000018581	Dnah11	0.155496981	0.05030673	1.62806324	0.00216503	0.043875823	UP
ENSMUSG00000029168	Dpysl5	21.28424089	8.211660016	1.374039835	2.21E-10	2.38E-08	UP
ENSMUSG00000034467	Dynlrb2	2.574696118	0.504825334	2.350545948	0.001695242	0.036291295	UP
ENSMUSG00000048747	E130114P18Rik	1.322163922	0.275966237	2.260337375	0.00000185	0.000110483	UP
ENSMUSG00000010476	Ebf3	11.91729018	5.532288494	1.107107929	0.000000731	0.000049	UP
ENSMUSG00000001036	Epn2	4.837779049	2.020169774	1.259868338	6.45E-08	0.00000525	UP
ENSMUSG00000006154	Eps8l1	0.557591883	0.244286338	1.190636377	0.000627583	0.01645303	UP
ENSMUSG00000030199	Etv6	7.097533768	2.551393865	1.476032179	8.45E-11	9.63E-09	UP
ENSMUSG00000031444	F10	0.603667663	0.194156505	1.636534478	0.000210713	0.006758877	UP
ENSMUSG00000021492	F12	1.196852455	0	Inf	5.65E-12	7.25E-10	UP
ENSMUSG00000027249	F2	0.73637303	0.01134439	6.020385906	1.55E-15	2.86E-13	UP
ENSMUSG00000019874	Fabp7	54.82048909	24.23628546	1.177546596	0.000000155	0.0000119	UP
ENSMUSG00000030630	Fah	3.306942195	1.188761373	1.476038685	0.00000451	0.000249715	UP
ENSMUSG00000050821	Fam131a	1.008009565	0.449820886	1.164086773	0.001339387	0.029895483	UP
ENSMUSG00000051000	Fam160a1	0.683966294	0.261953455	1.384614741	0.0000605	0.002374207	UP
ENSMUSG00000015484	Fam163a	1.62987563	0.806280119	1.015408828	0.002487406	0.048171686	UP
ENSMUSG00000049154	Fam183b	3.839147427	1.490436701	1.365050857	0.002576311	0.049321581	UP
ENSMUSG00000020607	Fam84a	4.15729001	2.019356693	1.041747627	0.000138933	0.00478949	UP

ENSMUSG00000022358	Fbxo32	3.012846814	1.254529824	1.263980554	0.000000946	0.0000601	UP
ENSMUSG00000044966	Fbxo48	1.173585893	0.447845786	1.389849498	0.000658114	0.017137513	UP
ENSMUSG00000015852	Fcrls	1.128401769	0.374419986	1.591551481	0.00052461	0.014136008	UP
ENSMUSG00000028001	Fga	1.088993526	0	Inf	9.41E-19	2.46E-16	UP
ENSMUSG00000033831	Fgb	5.530512849	0.032860022	7.394936023	1.18E-48	1.63E-45	UP
ENSMUSG00000042826	Fgf11	0.985880095	0.455654659	1.113471373	0.001619906	0.034940807	UP
ENSMUSG00000033860	Fgg	2.84761148	0.183726788	3.954118423	3.65E-17	8.06E-15	UP
ENSMUSG00000021250	Fos	0.949084967	0.148572013	2.675374875	8.08E-08	0.00000644	UP
ENSMUSG00000040891	Foxa3	0.541433884	0	Inf	0.0000154	0.000734425	UP
ENSMUSG00000048377	Foxi2	3.674180001	1.522122813	1.271337539	0.000029	0.001280368	UP
ENSMUSG00000034227	Foxj1	6.218587023	2.24339731	1.470901664	6.41E-08	0.00000524	UP
ENSMUSG00000035615	Frmpd1	1.322163922	0.643944125	1.037893636	0.001099462	0.025850702	UP
ENSMUSG00000055978	Fut2	0.587558912	0.131096003	2.164109812	0.000538084	0.014418863	UP
ENSMUSG00000106896	G630022F23Rik	0.688180141	0.241625801	1.510011746	0.002593418	0.049551166	UP
ENSMUSG00000026994	Galnt3	0.934812015	0.288041132	1.698401435	0.0000175	0.000822898	UP
ENSMUSG00000033316	Galnt9	1.415793056	0.69979088	1.016614637	0.001575596	0.034106804	UP
ENSMUSG00000044576	Gareml	4.445546431	2.154160106	1.04523528	0.0000663	0.002548884	UP
ENSMUSG00000031451	Gas6	7.096442779	3.404016791	1.059857875	0.0000475	0.001929172	UP
ENSMUSG00000033066	Gas7	1.039174455	0.244889726	2.085233719	6.99E-13	9.75E-11	UP
ENSMUSG00000021944	Gata4	6.666257116	1.587553291	2.070071942	1.94E-17	4.47E-15	UP
ENSMUSG00000005836	Gata6	11.92208593	5.076207538	1.231813717	0.00000023	0.0000171	UP
ENSMUSG00000067724	Gbx1	3.307448747	0.86468568	1.935471098	3.05E-10	3.25E-08	UP
ENSMUSG00000059434	Gckr	0.14858243	0	Inf	0.0000286	0.001268627	UP
ENSMUSG00000072625	Gdf2	1.301592344	0	Inf	2.46E-16	4.96E-14	UP
ENSMUSG00000037660	Gdf7	2.310329274	0.934347917	1.30606672	0.002063944	0.042403874	UP
ENSMUSG00000022144	Gdnf	2.448289545	1.141755073	1.100520988	0.000397953	0.01120631	UP
ENSMUSG00000025089	Gfra1	3.425533122	1.454976599	1.235332585	0.000000166	0.0000128	UP
ENSMUSG00000030406	Gipr	0.349510347	0.083757607	2.061043039	0.00041851	0.011700156	UP
ENSMUSG00000047797	Gjb1	0.816126861	0.036671553	4.476060146	0.00000843	0.000436485	UP
ENSMUSG00000042357	Gjb5	3.575263651	1.600458671	1.159564212	0.00078831	0.019808985	UP
ENSMUSG00000000263	Glra1	3.686351392	0.971371491	1.924098549	1.02E-10	1.15E-08	UP
ENSMUSG00000087501	Gm12116	0.461028619	0.090394717	2.350545948	0.001695242	0.036291295	UP

ENSMUSG00000090015	Gm15446	2.392640023	0.993656392	1.2677844	0.0000343	0.001472236	UP
ENSMUSG00000085723	Gm15915	0.83106158	0.068940905	3.591523246	0.002503814	0.048171686	UP
ENSMUSG00000086465	Gm16049	0.942297827	0	Inf	0.002421289	0.047240738	UP
ENSMUSG00000092544	Gm20422	0.626688507	0	Inf	0.0000996	0.003593996	UP
ENSMUSG00000092470	Gm20518	1.386078602	0.427084552	1.698415452	0.00000847	0.000437549	UP
ENSMUSG00000096870	Gm21816	0.251206922	0.018060687	3.797951536	0.001410364	0.031227883	UP
ENSMUSG00000094439	Gm21969	0.448351451	0.028440864	3.978592873	0.000000271	0.0000199	UP
ENSMUSG00000095538	Gm21983	1.236522109	0.037040439	5.061043039	1.18E-08	0.00000108	UP
ENSMUSG00000096056	Gm21986	1.858358538	0.236443798	2.974459664	8.47E-17	1.76E-14	UP
ENSMUSG00000097971	Gm26917	13.88271895	5.696329186	1.285185723	2.28E-08	0.000002	UP
ENSMUSG00000098552	Gm27217	0.658112768	0	Inf	0.002421289	0.047240738	UP
ENSMUSG00000098530	Gm28051	13.04434121	0	Inf	1.68E-22	5.92E-20	UP
ENSMUSG00000100725	Gm28062	14.42820125	0.541389356	4.736081118	2.39E-52	4.2E-49	UP
ENSMUSG00000099664	Gm28511	2.450890714	0.417322276	2.554072318	0.002426099	0.04728696	UP
ENSMUSG00000101645	Gm28635	24.71984443	0	Inf	1.75E-113	3.39E-109	UP
ENSMUSG00000000594	Gm2a	6.906065416	2.699979773	1.354915401	4.38E-08	0.00000366	UP
ENSMUSG00000104184	Gm37818	1.2822046	0.434593203	1.560888973	0.0001104	0.003917382	UP
ENSMUSG00000104453	Gm37829	0.472133595	0.056573286	3.061002255	0.001187534	0.027292141	UP
ENSMUSG00000102780	Gm38253	0.38467179	0.026762865	3.845323557	0.00000128	0.0000793	UP
ENSMUSG00000103509	Gm38372	0.734956692	0.248652796	1.563526586	0.000704966	0.017971073	UP
ENSMUSG00000106099	Gm42664	2.419384077	0.985667725	1.295466523	0.0000524	0.002087513	UP
ENSMUSG00000106995	Gm42712	0.746743506	0.094738315	2.978592873	0.00000616	0.000327417	UP
ENSMUSG00000105053	Gm43064	1.08930717	0.258835381	2.073304087	0.00000807	0.000420282	UP
ENSMUSG00000106754	Gm43101	0.689551444	0	Inf	0.001268294	0.028559939	UP
ENSMUSG00000104621	Gm43185	0.209173139	0	Inf	0.002421289	0.047240738	UP
ENSMUSG00000087235	Gm4750	0.312636638	0.037461605	3.061002255	0.001187534	0.027292141	UP
ENSMUSG00000096606	Gm4980	0.499406959	0.082856838	2.591523246	0.000264517	0.00814747	UP
ENSMUSG00000073535	Gm5532	0.658467639	0.109246669	2.591523246	0.000264517	0.00814747	UP
ENSMUSG00000091721	Gm5549	0.280820252	0	Inf	0.002421289	0.047240738	UP
ENSMUSG00000073077	Gm7173	0.081099403	0.005830695	3.797951536	0.001410364	0.031227883	UP
ENSMUSG00000090555	Gm8893	0.794542306	0	Inf	0.0000533	0.002115395	UP
ENSMUSG00000068428	Gmnc	1.222644807	0.137983119	3.147441667	1.69E-11	2.08E-09	UP

ENSMUSG00000028777	Gnat3	1.279519073	0.229971864	2.476072381	0.000128569	0.004496217	UP
ENSMUSG00000046519	Golph3l	3.49194064	0.714122794	2.289784962	2.19E-20	7.06E-18	UP
ENSMUSG00000058396	Gpr182	1.908486923	0.823261534	1.213006578	0.00016586	0.005569097	UP
ENSMUSG00000072966	Gprasp2	2.184861048	0.283731013	2.944945771	9.18E-24	3.49E-21	UP
ENSMUSG00000050069	Grem2	3.704495191	0.936083263	1.984568195	1.93E-11	2.33E-09	UP
ENSMUSG00000001985	Grik3	3.059895381	0.837176866	1.869877976	3.13E-13	4.73E-11	UP
ENSMUSG00000059003	Grin2a	0.088675443	0.025165162	1.817106723	0.00122165	0.027943612	UP
ENSMUSG00000003974	Grm3	0.647308326	0.143715444	2.171237951	0.00040416	0.011348107	UP
ENSMUSG00000046182	Gsg1l	2.885352012	0.781896783	1.883697261	7.09E-10	7.31E-08	UP
ENSMUSG00000026879	Gsn	6.40577774	2.94237756	1.12239136	0.0000046	0.00025292	UP
ENSMUSG00000053129	Gsx1	3.535875928	1.16514812	1.601554282	0.00000594	0.000319452	UP
ENSMUSG00000033910	Gucy1a3	1.808782899	0.833509992	1.117747858	0.0000151	0.000732454	UP
ENSMUSG00000034450	Gulo	3.921093044	0.414558553	3.241608087	3.1E-17	6.99E-15	UP
ENSMUSG00000025075	Habp2	1.243527515	0	Inf	4.91E-23	1.79E-20	UP
ENSMUSG00000037335	Hand1	17.62025073	7.783637355	1.178718053	0.000000187	0.0000143	UP
ENSMUSG00000032338	Hcn4	5.242307519	2.587727489	1.018516289	0.0000695	0.00264675	UP
ENSMUSG00000028940	Hes2	0.938133705	0.189691944	2.306135145	0.0000154	0.000734425	UP
ENSMUSG00000036181	Hist1h1c	8.967572929	4.052961239	1.145741195	0.000034	0.001463468	UP
ENSMUSG00000018102	Hist1h2bc	17.8243622	8.165255198	1.126280572	0.0000476	0.001929172	UP
ENSMUSG00000003949	Hlf	0.400640231	0.172824543	1.212999189	0.001150645	0.026665791	UP
ENSMUSG00000027875	Hmgcs2	0.918771067	0.295778214	1.635189635	0.0000861	0.003184527	UP
ENSMUSG00000050100	Hmx2	0.789003	0.182325266	2.113516285	0.000819566	0.020382887	UP
ENSMUSG00000029556	Hnf1a	0.240583178	0.041510837	2.534975858	0.000421961	0.0117458	UP
ENSMUSG00000017950	Hnf4a	1.097893331	0.015179035	6.176513982	4.53E-27	2.14E-24	UP
ENSMUSG00000059005	Hnrnpa3	7.694175755	2.165330611	1.829179467	7.79E-15	1.31E-12	UP
ENSMUSG00000014704	Hoxa2	18.64061054	7.794145101	1.257986419	0.000000199	0.0000151	UP
ENSMUSG00000056445	Hoxaas2	3.235668892	1.42447929	1.183629337	0.00000595	0.000319481	UP
ENSMUSG00000084844	Hoxb3os	3.243033894	0.778820292	2.057981722	3.4E-12	4.46E-10	UP
ENSMUSG00000030895	Hpx	1.492423303	0.127060233	3.554072318	0.000000207	0.0000156	UP
ENSMUSG00000038239	Hrc	4.187273297	1.986890762	1.075498527	0.00024256	0.007591855	UP
ENSMUSG00000046607	Hrk	0.529632247	0.197317657	1.424470903	0.001065918	0.02512298	UP
ENSMUSG00000059970	Hspa2	3.547335654	1.589848631	1.157846433	0.000167466	0.0056133	UP

ENSMUSG00000049511	Htr1b	0.658200284	0.219214194	1.586185435	0.000112166	0.003972763	UP
ENSMUSG00000032269	Htr3a	1.477686796	0.240531571	2.619042339	0.000000898	0.000058	UP
ENSMUSG00000003541	Ier3	4.927120496	1.328401895	1.891053068	0.000000777	0.0000512	UP
ENSMUSG00000045777	Ifitm10	0.237717083	0.059617468	1.995438573	0.000212314	0.006789435	UP
ENSMUSG00000031111	Igsf1	0.954760314	0.356413513	1.421586565	0.0000417	0.001724465	UP
ENSMUSG00000034275	Igsf9b	1.552014711	0.63528027	1.288677113	0.0000511	0.002042994	UP
ENSMUSG00000018654	Ikzf1	1.300410447	0.565577995	1.201169157	0.0000118	0.000594504	UP
ENSMUSG00000026072	Il1r1	0.916694348	0.267874956	1.774881072	0.000000046	0.00000381	UP
ENSMUSG00000046192	Iqub	0.190795347	0	Inf	0.000667207	0.017281373	UP
ENSMUSG00000006529	Itih1	0.280265999	0	Inf	0.0000286	0.001268627	UP
ENSMUSG00000037254	Itih2	17.37306658	0.621561503	4.804811469	7.4E-59	2.39E-55	UP
ENSMUSG00000006522	Itih3	0.278125085	0.062485867	2.154132051	0.001822828	0.038520607	UP
ENSMUSG00000092083	Kcnb2	0.12036744	0.026626262	2.176523393	0.000196752	0.006428106	UP
ENSMUSG00000039639	Kcne1	0.494543327	0.099997351	2.306135145	0.000382556	0.010851606	UP
ENSMUSG00000059742	Kcnh7	0.136851218	0.030889033	2.147441667	0.0000987	0.003587073	UP
ENSMUSG00000033854	Kcnk10	2.291643046	1.062688919	1.108663005	0.001054355	0.024880727	UP
ENSMUSG00000056258	Kcnq3	0.451391878	0.106754494	2.080083653	1.73E-08	0.00000157	UP
ENSMUSG00000028631	Kcnq4	2.913917004	1.322312058	1.139897102	0.0000683	0.002610847	UP
ENSMUSG00000036915	Kirrel2	1.32932919	0.324263812	2.03545848	4.28E-10	0.000000045	UP
ENSMUSG00000026308	Klhl30	2.048293039	0.891676715	1.19982948	0.000462519	0.012638704	UP
ENSMUSG00000044938	Klhl31	0.303917938	0.086754708	1.808667872	0.00179023	0.038030606	UP
ENSMUSG00000035298	Klhl35	1.808627126	0.533820671	1.760467929	0.00000311	0.000177167	UP
ENSMUSG00000066129	Kndc1	0.262600695	0.069070221	1.926734984	0.000430003	0.011884267	UP
ENSMUSG00000022905	Kpna1	9.859433333	4.249901916	1.214075185	0.00000004	0.00000335	UP
ENSMUSG00000023043	Krt18	84.04443508	41.85257594	1.005835875	0.000005	0.00027379	UP
ENSMUSG00000020911	Krt19	16.63828036	8.15355381	1.029005418	0.0000134	0.0006583	UP
ENSMUSG00000049382	Krt8	120.6615753	41.51368968	1.539307255	1.53E-12	2.08E-10	UP
ENSMUSG00000061578	Ksr2	0.477315106	0.235186393	1.021137403	0.001769569	0.037674308	UP
ENSMUSG00000026639	Lamb3	0.841788966	0.209496476	2.006532624	2.85E-08	0.00000243	UP
ENSMUSG00000027270	Lamp5	1.100061588	0.49130826	1.162883898	0.000681198	0.017526595	UP
ENSMUSG00000034839	Larp6	0.785071275	0.179585406	2.128153527	0.00059626	0.015802938	UP
ENSMUSG00000021798	Ldb3	5.018432577	2.160733064	1.21571598	0.000000551	0.0000385	UP

ENSMUSG00000082330	Ldha-ps	11.84282626	0.062912906	7.556441699	2.95E-34	2.29E-31	UP
ENSMUSG00000022025	Lect1	1.583403651	0.422503527	1.905993792	0.000000598	0.0000415	UP
ENSMUSG00000021539	Lect2	0.926651186	0	Inf	0.000352487	0.010177462	UP
ENSMUSG00000044505	Lingo4	0.22295558	0.017173756	3.698478785	0.002503814	0.048171686	UP
ENSMUSG00000036832	Lpar3	2.544280853	0.88920629	1.516667873	0.0000145	0.000706831	UP
ENSMUSG00000045201	Lrrc3b	0.980979764	0.366191143	1.42162648	0.001489598	0.032576213	UP
ENSMUSG00000047085	Lrrc4b	8.105512455	1.785659576	2.182446334	3.66E-17	8.06E-15	UP
ENSMUSG00000019892	Lrriq1	0.108781009	0	Inf	0.000187	0.00620366	UP
ENSMUSG00000043110	Lrrn4	6.109432318	2.207437988	1.468665423	1.07E-08	0.000000986	UP
ENSMUSG00000108715	Ltbp4	0.471935593	0	Inf	7.04E-08	0.00000571	UP
ENSMUSG00000036446	Lum	61.48837377	27.87586806	1.14129692	0.000000206	0.0000156	UP
ENSMUSG00000001089	Luzp1	7.258747076	3.58658626	1.017109219	0.00000332	0.000187462	UP
ENSMUSG00000026344	Lypd1	0.61776774	0.275042737	1.167408728	0.000626988	0.01645303	UP
ENSMUSG00000030787	Lyve1	1.00195959	0.360174577	1.476056068	0.002418425	0.047240738	UP
ENSMUSG00000074651	Mcidas	0.739772922	0.053184657	3.798000476	0.00000215	0.000126467	UP
ENSMUSG00000005583	Mef2c	0.565177989	0.199691174	1.50093471	4.18E-09	0.000000397	UP
ENSMUSG00000034258	Mfsd7c	0.726646106	0.218684162	1.732404168	0.000820646	0.020383575	UP
ENSMUSG00000038244	Mical2	3.29240899	1.072394685	1.618307587	9.66E-12	1.2E-09	UP
ENSMUSG00000012519	Mlkl	0.16927835	0	Inf	0.002421289	0.047240738	UP
ENSMUSG00000025355	Mmp19	0.663891735	0.146858279	2.176523393	0.000196752	0.006428106	UP
ENSMUSG00000029061	Mmp23	1.404235934	0.651533328	1.107874467	0.00223791	0.045069919	UP
ENSMUSG00000046805	Mpeg1	0.562403523	0.187722823	1.583001568	0.002039309	0.042047643	UP
ENSMUSG00000032584	Mst1r	0.774762508	0.232984375	1.733520937	0.00000167	0.000100714	UP
ENSMUSG00000025469	Msx3	9.875672354	2.319561092	2.090027129	1.41E-17	3.33E-15	UP
ENSMUSG00000031762	Mt2	156.8116783	76.33596724	1.038598128	0.00000326	0.000184957	UP
ENSMUSG00000024905	Mtl5	0.101959505	0	Inf	0.002421289	0.047240738	UP
ENSMUSG00000028158	Mttp	3.862209342	1.888054212	1.032526174	0.0000283	0.001265373	UP
ENSMUSG00000002100	Mybpc3	17.35107576	6.437638386	1.430421667	2.39E-10	2.56E-08	UP
ENSMUSG00000068745	Mybphl	3.957780503	1.079330612	1.874554756	0.0000026	0.000150831	UP
ENSMUSG00000028654	Mycl	19.95896638	8.507423705	1.230242796	2.53E-08	0.0000022	UP
ENSMUSG00000108439	Myh14	0.512501068	0.062567058	3.034079839	9.93E-08	0.00000785	UP
ENSMUSG00000040752	Myh6	40.37773333	19.27942606	1.066497821	0.000000769	0.0000508	UP

ENSMUSG00000053093	Myh7	21.00076777	8.041142875	1.384969603	1.92E-10	2.11E-08	UP
ENSMUSG00000061086	Myl4	25.7328186	11.80007295	1.124813711	0.000000279	0.0000204	UP
ENSMUSG00000024300	Myo1f	0.566760661	0.236601975	1.260277504	0.002123855	0.043358877	UP
ENSMUSG00000033590	Myo5c	0.836266654	0.399790737	1.064717992	0.001799486	0.038185375	UP
ENSMUSG00000024049	Myom1	5.941515662	2.643448391	1.168409844	0.000000783	0.0000514	UP
ENSMUSG00000043943	Naalad2	0.600184995	0.218669957	1.456652225	0.00027273	0.008339195	UP
ENSMUSG00000002881	Nab1	2.492716562	1.220940628	1.029725801	0.000135363	0.004692684	UP
ENSMUSG00000005125	Ndrg1	4.96883064	1.391440049	1.836327618	3.28E-14	5.17E-12	UP
ENSMUSG00000040280	Ndufa4l2	9.009435538	4.108146051	1.132949248	0.000268755	0.008251749	UP
ENSMUSG00000092274	Neat1	0.411865959	0.146374864	1.492507062	0.000000761	0.0000507	UP
ENSMUSG00000053702	Nebl	1.616978751	0.78912308	1.034978479	0.0000131	0.000647925	UP
ENSMUSG00000038624	Nepn	2.610889571	0.121361655	4.427156874	1.76E-18	4.32E-16	UP
ENSMUSG00000039103	Nexn	1.687648166	0.810734044	1.057713538	0.000138896	0.00478949	UP
ENSMUSG00000026442	Nfasc	3.845999175	1.810502034	1.086968656	0.00000153	0.0000931	UP
ENSMUSG00000069670	Nkain2	0.212431923	0	Inf	0.0000996	0.003593996	UP
ENSMUSG00000055761	Nkain3	0.336371312	0.095003002	1.824009667	0.000602715	0.015952177	UP
ENSMUSG00000015579	Nkx2-5	7.003745118	3.324977282	1.074782098	0.000186934	0.00620366	UP
ENSMUSG00000044186	Nkx2-6	5.83545408	1.656095761	1.817058826	0.000000332	0.0000239	UP
ENSMUSG00000029361	Nos1	0.25759079	0.071550582	1.848045608	0.0000194	0.000898438	UP
ENSMUSG00000006649	Nphs1	0.370276549	0.081908199	2.176523393	0.000196752	0.006428106	UP
ENSMUSG00000026241	Nppc	2.729679731	0.360451102	2.920856225	0.000000519	0.0000367	UP
ENSMUSG00000048938	Nr1h5	1.16094468	0.125196683	3.213030984	5.59E-10	5.79E-08	UP
ENSMUSG00000026398	Nr5a2	0.387155973	0.127376232	1.603818804	0.000206754	0.006695046	UP
ENSMUSG00000052854	Nrk	2.498574496	1.167896392	1.097192939	0.00000328	0.000185909	UP
ENSMUSG00000031767	Nudt7	1.01650272	0.498287643	1.028563373	0.000885396	0.021686033	UP
ENSMUSG00000090061	Nwd2	0.384261474	0.136709839	1.49097126	0.0000355	0.001513145	UP
ENSMUSG00000040258	Nxph4	9.045005233	3.550465364	1.349113205	0.000000811	0.0000527	UP
ENSMUSG00000061462	Obscn	0.48120487	0.225871071	1.091151738	0.0000477	0.001929172	UP
ENSMUSG00000015001	Oc90	6.225841207	0	Inf	1.79E-43	2.17E-40	UP
ENSMUSG00000009654	Oit3	2.826359826	0.357344461	2.983557817	2.72E-15	4.74E-13	UP
ENSMUSG00000045591	Olig3	23.76344625	6.238580069	1.929454469	9.07E-16	1.72E-13	UP
ENSMUSG00000043013	Onecut1	2.976509558	1.162853681	1.355951949	0.000000645	0.0000442	UP

ENSMUSG00000045518	Onecut3	1.409858502	0.602928404	1.225491776	0.000247991	0.007724411	UP
ENSMUSG00000074063	Osgin1	0.135136804	0.011210305	3.591523246	0.002503814	0.048171686	UP
ENSMUSG00000009487	Otog	0.138716375	0.012466064	3.476060146	0.00005	0.002005072	UP
ENSMUSG00000091455	Otogl	0.213436265	0.044264136	2.269595151	0.000758987	0.019146643	UP
ENSMUSG00000027788	Otol1	0.483494514	0	Inf	0.0000286	0.001268627	UP
ENSMUSG00000021685	Otp	2.916255663	1.104493673	1.400732049	0.00000336	0.000188684	UP
ENSMUSG00000033510	Otud7a	0.844741469	0.359901056	1.230909542	0.000932621	0.022557549	UP
ENSMUSG00000027071	P2rx3	2.015436305	0.789007003	1.35298218	0.0000153	0.000734425	UP
ENSMUSG00000018906	P4ha2	8.645659657	4.057955456	1.091223016	0.00000129	0.0000797	UP
ENSMUSG00000027188	Pamr1	0.334991243	0.101891352	1.717091775	0.001648005	0.035476057	UP
ENSMUSG00000097063	Pantr2	3.256115662	1.300562737	1.324015953	0.000438362	0.012080842	UP
ENSMUSG00000058441	Panx2	0.565145502	0.165276149	1.773743806	0.0000636	0.00247082	UP
ENSMUSG00000064225	Paqr9	1.2071538	0.239417865	2.334006785	1.6E-12	2.17E-10	UP
ENSMUSG00000102206	Pcdha11	0.049543826	0	Inf	0.000187	0.00620366	UP
ENSMUSG00000073591	Pcdhb22	0.22238232	0.056426674	1.978592873	0.000883481	0.021666533	UP
ENSMUSG00000103144	Pcdhga1	1.535455121	0.189245817	3.020333026	1.12E-18	2.88E-16	UP
ENSMUSG00000103472	Pcdhga7	5.241744312	0.781094182	2.746478563	4.1E-26	1.8E-23	UP
ENSMUSG00000023036	Pcdhga8	4.565532768	0.446227721	3.354931179	1.07E-33	7.7E-31	UP
ENSMUSG00000103897	Pcdhga8	0.98178032	0.026468643	5.213044093	2.53E-15	4.45E-13	UP
ENSMUSG00000103585	Pcdhgb4	2.510496658	0.682469602	1.879136111	2.18E-12	2.93E-10	UP
ENSMUSG00000102543	Pcdhgc5	5.217516038	0.240983978	4.436353996	1.58E-53	3.06E-50	UP
ENSMUSG00000021196	Pfkp	3.96000147	1.679797448	1.237213684	0.00000012	0.00000937	UP
ENSMUSG00000026873	Phf19	0.961417844	0.398196645	1.271682515	0.0000591	0.002321632	UP
ENSMUSG00000007946	Phox2a	4.545679764	2.21766946	1.035451702	0.001212124	0.027791355	UP
ENSMUSG00000012520	Phox2b	8.644782272	1.254984577	2.784159994	6.9E-26	2.97E-23	UP
ENSMUSG00000009646	Pla2g12b	0.738605711	0	Inf	0.00000248	0.000144877	UP
ENSMUSG00000029322	Plac8	5.453012732	2.138424311	1.35050538	0.00000158	0.0000961	UP
ENSMUSG00000051413	Plagl2	12.60058855	3.211160381	1.972324494	1.59E-18	3.95E-16	UP
ENSMUSG00000030701	Plekhb1	0.608994395	0.272313966	1.161157975	0.001864264	0.039120213	UP
ENSMUSG00000032068	Plet1	1.928677771	0.722206223	1.417129372	0.000442871	0.012153239	UP
ENSMUSG00000059481	Plg	3.371173413	0	Inf	3.61E-30	2.12E-27	UP
ENSMUSG00000049493	Pls1	2.055021742	0.98666753	1.058517721	0.000582763	0.015487579	UP

ENSMUSG00000046204	Pnma2	0.515832591	0.161959978	1.671265579	0.0000292	0.001285788	UP
ENSMUSG00000045731	Pnoc	1.022624776	0.232168165	2.139034818	0.000287197	0.008693986	UP
ENSMUSG00000027750	Postn	23.83269584	8.871097531	1.425757641	4.25E-11	4.99E-09	UP
ENSMUSG00000031688	Pou4f2	0.507286573	0.117225359	2.113516285	0.000819566	0.020382887	UP
ENSMUSG00000040653	Ppp1r14c	6.715482726	3.224235909	1.058533802	0.0000586	0.002309088	UP
ENSMUSG00000067279	Ppp1r3c	6.361962231	2.601618106	1.290062604	0.00000113	0.0000716	UP
ENSMUSG00000040478	Prdm13	1.220564815	0.3927013	1.63604463	0.000000074	0.00000597	UP
ENSMUSG00000036892	Prodh2	0.124427752	0	Inf	0.002421289	0.047240738	UP
ENSMUSG00000042320	Prox2	0.553633959	0.191501135	1.531579498	0.000674911	0.017434309	UP
ENSMUSG00000031445	Proz	0.314680532	0	Inf	0.000352487	0.010177462	UP
ENSMUSG00000036480	Prss56	0.505917808	0	Inf	0.0000154	0.000734425	UP
ENSMUSG00000048776	Pthlh	0.421538464	0.026740001	3.978592873	0.000447975	0.012275918	UP
ENSMUSG00000059456	Ptk2b	0.661494795	0.252093599	1.391770316	0.0000195	0.000902356	UP
ENSMUSG00000030854	Ptpn5	2.433197752	0.726649352	1.743522318	7.74E-09	0.000000724	UP
ENSMUSG00000019832	Rab32	4.047902675	1.989664884	1.024649143	0.000922793	0.022403758	UP
ENSMUSG00000009281	Rarres2	1.463659061	0.589749504	1.311405333	0.00029373	0.008836522	UP
ENSMUSG00000042129	Rassf4	7.446474518	3.214627229	1.211906102	7.65E-08	0.00000615	UP
ENSMUSG00000070780	Rbm47	0.201555105	0.064137645	1.651931042	0.000155023	0.005269133	UP
ENSMUSG00000024990	Rbp4	19.19995582	1.592758378	3.59150366	2E-31	1.25E-28	UP
ENSMUSG00000025350	Rdh5	0.8884874	0.359314959	1.306102317	0.00101524	0.024163712	UP
ENSMUSG00000042453	Reln	2.247054884	0.706014898	1.67026483	6.54E-13	9.39E-11	UP
ENSMUSG00000070645	Ren1	0.513974747	0	Inf	0.0000996	0.003593996	UP
ENSMUSG00000040855	Reps2	0.226696406	0.062863766	1.850460905	0.001255385	0.02847989	UP
ENSMUSG00000023070	Rgn	0.361204394	0	Inf	0.002421289	0.047240738	UP
ENSMUSG00000021876	Rnase4	5.147497143	2.446290246	1.073275538	0.000234322	0.007381706	UP
ENSMUSG00000001313	Rnd2	30.92063488	10.18820657	1.601669826	2.56E-12	3.4E-10	UP
ENSMUSG00000032128	Robo3	2.909181442	0.825617965	1.81706701	6.82E-13	9.71E-11	UP
ENSMUSG00000032238	Rora	0.487793584	0.126252947	1.949953715	2.42E-08	0.00000211	UP
ENSMUSG00000107482	RP23-74K24.2	2.3702719	0.052165285	5.505818726	2.35E-11	2.82E-09	UP
ENSMUSG00000108634	RP24-402O2.2	3.405618465	0.983767728	1.791527184	0.000000533	0.0000374	UP
ENSMUSG00000024033	Rsph1	2.593191351	0.914236946	1.50408864	0.000899245	0.021997448	UP
ENSMUSG00000006586	Runx1t1	1.529217168	0.583546374	1.389874088	6.27E-08	0.00000515	UP

ENSMUSG00000021313	Ryr2	1.901726689	0.668764509	1.507739731	8.38E-10	8.36E-08	UP
ENSMUSG00000009614	Sardh	0.855418983	0.313304969	1.449063572	0.0000337	0.001459371	UP
ENSMUSG00000085272	Sbk3	0.194285251	0.016116978	3.591523246	0.002503814	0.048171686	UP
ENSMUSG00000034810	Scn7a	0.181945823	0.025592127	2.829736997	0.000339552	0.010012907	UP
ENSMUSG00000060257	Scrt2	14.3451675	6.527374481	1.135990098	0.000000952	0.0000603	UP
ENSMUSG00000029173	Sepsecs	1.912060305	0.949849932	1.009356522	0.0000798	0.003002526	UP
ENSMUSG00000061947	Serpina10	0.449648834	0	Inf	1.25E-08	0.00000113	UP
ENSMUSG00000066366	Serpina1a	1.200975885	0	Inf	4.16E-13	6.15E-11	UP
ENSMUSG00000071178	Serpina1b	7.059274137	0.156272607	5.497383031	1.63E-41	1.66E-38	UP
ENSMUSG00000071177	Serpina1d	1.207572656	0.044905553	4.749072312	0.000000523	0.0000369	UP
ENSMUSG00000072849	Serpina1e	1.821972722	0	Inf	1.99E-14	3.21E-12	UP
ENSMUSG00000060807	Serpina6	8.939486926	0.095764928	6.544550823	3.88E-42	4.42E-39	UP
ENSMUSG00000026715	Serpinc1	0.52367777	0.03493211	3.906053663	6.67E-15	1.14E-12	UP
ENSMUSG00000022766	Serpind1	2.171268954	0.032748282	6.050975399	3.44E-25	1.39E-22	UP
ENSMUSG00000038224	Serpinf2	7.691248169	0.191774342	5.325736128	2.9E-49	4.33E-46	UP
ENSMUSG00000070436	Serpinh1	516.7402866	248.0762184	1.058655925	0.000000698	0.0000471	UP
ENSMUSG00000000632	Sez6	0.71007737	0.341054117	1.057975554	0.001107662	0.026011923	UP
ENSMUSG00000018822	Sfrp5	4.2771957	1.64735759	1.376511465	0.0000158	0.000749471	UP
ENSMUSG00000057719	Sh3rf2	0.249968863	0.065570888	1.93062105	0.000612799	0.016130943	UP
ENSMUSG00000005202	Shbg	0.726792842	0	Inf	0.00000136	0.0000836	UP
ENSMUSG00000096883	Shisa8	0.997502569	0.173498729	2.523395462	0.0000822	0.003081214	UP
ENSMUSG00000037762	Slc16a9	1.141698902	0.41535922	1.458750737	0.000152401	0.005189138	UP
ENSMUSG00000000154	Slc22a18	0.370985543	0.047066422	2.978592873	0.002009464	0.041593325	UP
ENSMUSG00000041771	Slc24a4	0.148809296	0.010698742	3.797951536	0.001410364	0.031227883	UP
ENSMUSG00000002346	Slc25a42	0.824148557	0.398907788	1.046849125	0.001042672	0.024695272	UP
ENSMUSG00000042268	Slc26a9	0.336980128	0	Inf	1.3E-09	0.000000128	UP
ENSMUSG00000027690	Slc2a2	0.114011376	0	Inf	0.000352487	0.010177462	UP
ENSMUSG00000023169	Slc38a1	35.81358171	16.5545776	1.11327661	0.000000244	0.000018	UP
ENSMUSG00000000792	Slc5a5	0.515439833	0.12827529	2.006560746	0.001887669	0.039494279	UP
ENSMUSG00000030310	Slc6a1	0.339956166	0.14053921	1.274376046	0.00228741	0.045734028	UP
ENSMUSG00000039728	Slc6a5	0.437168974	0.143830756	1.603818804	0.000206754	0.006695046	UP
ENSMUSG00000032548	Slco2a1	0.243879044	0.086301288	1.498711798	0.000329839	0.009801087	UP

ENSMUSG00000023886	Smoc2	12.42116773	5.857696655	1.08439542	0.00000566	0.000306953	UP
ENSMUSG00000045667	Smtnl2	8.946420433	4.347521804	1.041117296	0.00000625	0.00033097	UP
ENSMUSG00000023045	Soat2	1.226654071	0.212598141	2.528527566	0.000000768	0.0000508	UP
ENSMUSG00000072941	Sod3	1.382077902	0.586143472	1.237513191	0.000638974	0.016683946	UP
ENSMUSG00000043531	Sorcs1	0.736142931	0.342073868	1.105678013	0.000587082	0.015580984	UP
ENSMUSG00000026163	Sphkap	0.796341866	0.309006795	1.365749344	0.00000174	0.00010387	UP
ENSMUSG00000008193	Spib	0.247313783	0	Inf	0.000352487	0.010177462	UP
ENSMUSG00000054162	Spock3	0.465674542	0.191053741	1.285343509	0.000345775	0.010165478	UP
ENSMUSG00000026295	Spp2	0.55485582	0	Inf	0.000352487	0.010177462	UP
ENSMUSG00000039860	Srrm3	1.207416377	0.520847583	1.212990114	0.0000841	0.003120514	UP
ENSMUSG00000034616	Ssh3	1.713580825	0.841480697	1.026012159	0.001995261	0.041432145	UP
ENSMUSG00000047904	Sstr2	1.90581551	0.636471667	1.58224027	0.000000604	0.0000418	UP
ENSMUSG00000056812	St8sia3	1.296832359	0.623262348	1.057080529	0.000286598	0.00868943	UP
ENSMUSG00000035459	Stab2	0.434084747	0.061594353	2.817106723	1.96E-09	0.000000191	UP
ENSMUSG00000028801	Stpg1	0.40734753	0.054910826	2.891097646	0.000206996	0.006695046	UP
ENSMUSG00000038651	Sycp2l	0.268747613	0	Inf	0.000667207	0.017281373	UP
ENSMUSG00000009394	Syn2	0.489355644	0.133606785	1.872890069	0.00000289	0.000164975	UP
ENSMUSG00000025400	Tac2	14.91935679	6.692681264	1.156529126	0.0000257	0.001153491	UP
ENSMUSG00000051397	Tacstd2	8.765678929	3.319407557	1.400940062	0.000000337	0.0000242	UP
ENSMUSG00000024339	Tap2	0.281568625	0.07007277	2.006560746	0.001887669	0.039494279	UP
ENSMUSG00000009097	Tbx1	13.55640331	5.49554905	1.302638933	0.000000227	0.0000169	UP
ENSMUSG00000037477	Tbx10	0.68597642	0	Inf	0.00000454	0.000250474	UP
ENSMUSG00000031965	Tbx20	7.057780997	2.373417135	1.572248983	2.61E-11	3.12E-09	UP
ENSMUSG00000028011	Tdo2	0.151162643	0.012539732	3.591523246	0.002503814	0.048171686	UP
ENSMUSG00000049336	Tenm2	1.068110923	0.472012055	1.178165867	0.0000129	0.000643992	UP
ENSMUSG00000023990	Tfeb	0.428162176	0.156180085	1.454946852	0.000306105	0.009137867	UP
ENSMUSG00000029999	Tgfa	1.058300994	0.111552905	3.245950022	7.67E-16	1.49E-13	UP
ENSMUSG00000021702	Thbs4	3.617962101	1.553771492	1.219402949	0.0000129	0.000643992	UP
ENSMUSG00000032625	Thsd7a	1.678058585	0.714237326	1.232317648	0.000000786	0.0000514	UP
ENSMUSG00000042581	Thsd7b	1.003463281	0.356476536	1.493108803	8.06E-08	0.00000644	UP
ENSMUSG00000034850	Tmem127	13.57812376	1.716989078	2.983331373	6.56E-34	4.89E-31	UP
ENSMUSG00000029569	Tmem168	5.626351349	2.681238765	1.06929995	0.00000549	0.000298875	UP

ENSMUSG00000026188	Tmem169	2.490837525	1.206847596	1.045387419	0.000700521	0.017928534	UP
ENSMUSG00000057716	Tmem178b	1.225062797	0.543272066	1.173108932	0.0000349	0.001488192	UP
ENSMUSG00000027341	Tmem230	4.133243558	2.032396207	1.024092702	0.0000472	0.001919232	UP
ENSMUSG00000106715	Tmem265	1.066174833	0.127751717	3.061029444	0.0000468	0.001911253	UP
ENSMUSG00000022754	Tmem45a	3.178634572	0.628154902	2.339214893	3.65E-13	5.49E-11	UP
ENSMUSG00000028364	Tnc	11.4352559	4.863367167	1.233461232	3.33E-08	0.00000281	UP
ENSMUSG00000049734	Trex1	1.438982183	0.409904949	1.811687417	0.00011585	0.004088309	UP
ENSMUSG00000032554	Trf	13.70982595	2.10326669	2.704506558	1.69E-28	8.59E-26	UP
ENSMUSG00000050663	Trhde	0.119801484	0.012304342	3.283406329	0.000243751	0.007616814	UP
ENSMUSG00000032715	Trib3	3.058993006	0.888920074	1.782931196	1.75E-08	0.00000157	UP
ENSMUSG00000039853	Trim14	0.247799458	0.055672662	2.154132051	0.001822828	0.038520607	UP
ENSMUSG00000024457	Trim26	5.398566143	1.692041842	1.673811035	5.65E-13	8.23E-11	UP
ENSMUSG00000068735	Trp53i11	15.94151632	6.167070405	1.37013164	3.6E-10	3.81E-08	UP
ENSMUSG00000029026	Trp73	0.280426696	0.071154693	1.978592873	0.000883481	0.021666533	UP
ENSMUSG00000052387	Trpm3	0.580418315	0.25922577	1.162883898	0.000681198	0.017526595	UP
ENSMUSG00000029074	Ttll10	0.22259394	0.031309607	2.829736997	0.000339552	0.010012907	UP
ENSMUSG00000061808	Ttr	19.28565558	0.205408832	6.552886166	1.34E-49	2.17E-46	UP
ENSMUSG00000039891	Txlnb	2.115581084	0.934325245	1.179057227	0.000032	0.001391587	UP
ENSMUSG00000029260	Ugt2b34	0.195130478	0.010020544	4.283406329	0.0000458	0.00188356	UP
ENSMUSG00000035811	Ugt2b35	0.224651359	0	Inf	0.000352487	0.010177462	UP
ENSMUSG00000018845	Unc45b	6.713214336	2.926421266	1.197866241	0.000000664	0.0000453	UP
ENSMUSG00000020407	Upp1	3.15977782	1.474066358	1.100021648	0.000164653	0.005538178	UP
ENSMUSG00000030107	Usp18	0.185240684	0	Inf	0.001268294	0.028559939	UP
ENSMUSG00000034993	Vat1	63.62976588	31.27737339	1.02458245	0.00000256	0.00014941	UP
ENSMUSG00000037428	Vgf	0.178329347	0.021368268	3.061002255	0.001187534	0.027292141	UP
ENSMUSG00000054459	Vsnl1	2.761300751	0.938683687	1.556637037	0.000029	0.001280368	UP
ENSMUSG00000050666	Vstm4	1.882827788	0.864042791	1.123726384	0.001261976	0.028559939	UP
ENSMUSG00000010797	Wnt2	10.11648754	4.94138205	1.033721962	0.000037	0.001571596	UP
ENSMUSG00000057836	Xlr3a	0.608670332	0.231676568	1.39354891	0.000756638	0.019112256	UP
ENSMUSG00000073125	Xlr3b	0.999682751	0.43657573	1.195238398	0.000260791	0.008071189	UP
ENSMUSG00000037638	Zbtb42	1.629864207	0.246078325	2.727562277	3.63E-16	7.18E-14	UP
ENSMUSG00000031351	Zfp185	3.982782859	1.794982409	1.149807121	0.0000188	0.000873579	UP

ENSMUSG00000037855	Zfp365	0.901033707	0.448480619	1.006535436	0.001385032	0.030843221	UP
ENSMUSG00000047003	Zfp41	16.70051453	5.935211944	1.492521098	1.87E-11	2.28E-09	UP
ENSMUSG00000096795	Zfp433	0.349680861	0.094274611	1.891097646	0.001847602	0.038888056	UP
ENSMUSG00000023882	Zfp54	0.347275909	0.102136525	1.765583448	0.002545854	0.048786718	UP
ENSMUSG00000046311	Zfp62	2.331224473	0.820377569	1.506727976	2.01E-10	0.000000022	UP
ENSMUSG00000022306	Zfpm2	1.820551709	0.7299615	1.318483438	0.000017	0.00080252	UP
ENSMUSG00000010044	Zmynd10	0.50445257	0.116570468	2.113516285	0.000819566	0.020382887	UP

Table S2-653 DEGs of HET vs WT

gene_id	gene_name	HET	WT	log2FC	Pvalue	Qvalue	updown
ENSMUSG00000032656	03-Mar	0.291493214	0.644190665	-1.144025459	0.002156652	0.046774686	DOWN
ENSMUSG00000025702	08-Mar	1.550225378	4.380197653	-1.498517997	2.65E-11	3.99E-09	DOWN
ENSMUSG00000079502	1700101E01Rik	0.081642422	0.410355843	-2.329484616	0.000352577	0.010769073	DOWN
ENSMUSG00000043391	2510009E07Rik	5.863132339	12.68001761	-1.112813223	0.000000638	0.0000404	DOWN
ENSMUSG00000052419	2610001J05Rik	3.242647853	6.659576785	-1.038258139	0.00000644	0.000330738	DOWN
ENSMUSG00000041789	2700046A07Rik	0.475762004	2.559927725	-2.427791115	1.76E-11	2.7E-09	DOWN
ENSMUSG00000090031	4732440D04Rik	0.009232977	0.604694911	-6.033267776	6.21E-15	1.47E-12	DOWN
ENSMUSG00000097074	4833428L15Rik	0	4.325798573	#NAME?	4.85E-21	2.11E-18	DOWN
ENSMUSG00000059920	4930453N24Rik	2.295979334	6.760572205	-1.558035703	3.33E-11	4.87E-09	DOWN
ENSMUSG00000085488	4930557F10Rik	0	1.092986914	#NAME?	0.001268294	0.030629386	DOWN
ENSMUSG00000097908	4933404O12Rik	0.297216595	1.964988668	-2.724934418	1.75E-19	6.2E-17	DOWN
ENSMUSG00000086126	5730457N03Rik	0.532031125	2.537588786	-2.253875748	4.64E-11	6.53E-09	DOWN
ENSMUSG00000086141	9030622O22Rik	0.136431822	0.737814699	-2.435078348	0.000488104	0.014146821	DOWN
ENSMUSG00000087022	9130024F11Rik	0.023290307	0.193760968	-3.056476445	0.000126105	0.00448892	DOWN
ENSMUSG00000030111	A2m	0.395127477	0.912249304	-1.207109972	0.000173858	0.005935034	DOWN
ENSMUSG00000097868	A330069E16Rik	0.203599816	1.340959229	-2.719457213	0.001488068	0.034831857	DOWN
ENSMUSG00000048636	A730049H05Rik	0.029062183	0.498746407	-4.101091462	0.000000271	0.0000186	DOWN
ENSMUSG00000026842	Abl1	6.786185709	16.65972968	-1.295692176	1.07E-09	0.000000119	DOWN
ENSMUSG00000037872	Ackr1	0.098587684	0.743412879	-2.914684344	0.0000405	0.001674045	DOWN
ENSMUSG00000036040	Adamtsl2	0.552749644	1.215026949	-1.136290218	0.0000572	0.002217005	DOWN
ENSMUSG00000093738	AI606473	0.023773184	1.507549754	-5.986726686	1.58E-14	3.57E-12	DOWN
ENSMUSG00000053279	Aldh1a1	1.671227546	8.84267794	-2.403575168	5.79E-17	1.73E-14	DOWN
ENSMUSG00000015134	Aldh1a3	1.819363323	4.96534714	-1.448460908	4.35E-09	0.000000433	DOWN
ENSMUSG00000028766	Alpl	2.539851559	5.214383818	-1.037752599	0.0000233	0.001036254	DOWN
ENSMUSG00000050947	Amigo1	0.44165806	1.406369607	-1.670974051	3.48E-10	4.39E-08	DOWN
ENSMUSG00000022602	Arc	0.150544485	0.481722972	-1.678013862	0.000820646	0.021888836	DOWN
ENSMUSG00000035277	Arx	0.390279521	1.040735028	-1.415023136	0.0000337	0.001426651	DOWN
ENSMUSG00000059995	Atxn7l3	9.339906167	22.11465732	-1.243522926	7.76E-09	0.000000747	DOWN
ENSMUSG00000026432	Avpr1b	0.025909505	0.229051185	-3.144116641	0.001187534	0.029383016	DOWN
ENSMUSG00000097428	AW047730	0	0.579794448	#NAME?	2.27E-09	0.000000238	DOWN
ENSMUSG00000097623	B230323A14Rik	0.104691615	0.471773689	-2.171949057	0.001245444	0.030578852	DOWN

ENSMUSG00000034384	Barhl2	0.026023109	2.083058336	-6.3227661	1.11E-17	3.62E-15	DOWN
ENSMUSG00000045493	Bhlhe23	0.692696079	1.813414136	-1.388414023	0.000206368	0.006873302	DOWN
ENSMUSG00000061132	Blnk	0.086730663	0.451006679	-2.378534747	0.00000622	0.000320365	DOWN
ENSMUSG00000106775	C130093G08Rik	0.811236116	2.081043111	-1.359113065	0.0002523	0.008135182	DOWN
ENSMUSG00000004110	Cacna1e	0.056180216	0.131932577	-1.231666769	0.000955102	0.024453421	DOWN
ENSMUSG00000062961	Ccdc177	0.727537405	1.824809348	-1.326652415	0.00000735	0.000367744	DOWN
ENSMUSG00000049305	Ccdc71	3.425109762	8.257056391	-1.269477336	2.41E-08	0.00000208	DOWN
ENSMUSG00000053166	Cdh22	0.353166626	1.395688684	-1.982556254	7.62E-08	0.00000598	DOWN
ENSMUSG00000036510	Cdh8	0.140037371	0.3823013	-1.448898221	0.000395595	0.01187467	DOWN
ENSMUSG00000090071	Cdk5r2	1.741927715	3.913905501	-1.167924166	0.0000518	0.002075222	DOWN
ENSMUSG00000029646	Cdx2	1.623963413	3.267046147	-1.008467703	0.001212124	0.029914164	DOWN
ENSMUSG00000031326	Cdx4	0	3.922456977	#NAME?	2.07E-27	1.52E-24	DOWN
ENSMUSG00000038192	Cer1	0	2.879811394	#NAME?	1.01E-19	3.73E-17	DOWN
ENSMUSG00000053119	Chmp3	4.47031457	12.41999882	-1.474216775	1.21E-10	1.64E-08	DOWN
ENSMUSG00000037625	Cldn11	1.834775482	3.682355404	-1.005025342	0.001514896	0.035380216	DOWN
ENSMUSG00000044681	Cnpy1	0.091479817	1.57293061	-4.103857732	2.96E-29	2.7E-26	DOWN
ENSMUSG00000055022	Cntn1	0.123637311	0.326403479	-1.40054225	0.000464327	0.01353473	DOWN
ENSMUSG00000039419	Cntnap2	0.802345889	1.618280736	-1.012165687	0.0000235	0.001037633	DOWN
ENSMUSG00000039714	Cplx3	0.021263232	0.508412501	-4.579566667	0.00000843	0.000410602	DOWN
ENSMUSG00000034361	Cpne2	2.064671344	4.239399287	-1.037947702	0.0000476	0.001921796	DOWN
ENSMUSG00000030905	Crym	4.468785012	10.45680889	-1.226488105	0.00000018	0.0000129	DOWN
ENSMUSG00000028804	Csmd2	0.050739297	0.154784455	-1.60908514	0.000764908	0.020661142	DOWN
ENSMUSG00000024987	Cyp26a1	2.715983192	6.002272866	-1.144034355	0.0000792	0.002974946	DOWN
ENSMUSG00000062432	Cyp26c1	0	2.149985796	#NAME?	1.04E-15	2.73E-13	DOWN
ENSMUSG00000097131	D230017M19Rik	0.026675485	0.18493784	-2.793454013	0.000910749	0.023635451	DOWN
ENSMUSG00000030507	Dbx1	7.830014892	17.87288734	-1.190685762	0.000000388	0.0000261	DOWN
ENSMUSG00000022129	Dct	0.053239966	1.079786672	-4.342092863	1.27E-08	0.00000116	DOWN
ENSMUSG00000069045	Ddx3y	0	1.045806458	#NAME?	3.01E-32	3.2E-29	DOWN
ENSMUSG00000087050	Dhrs13os	0.049557663	0.438111076	-3.144116641	0.001187534	0.029383016	DOWN
ENSMUSG00000034480	Diap2	0.198948473	0.482776643	-1.278961055	0.0000231	0.001029993	DOWN
ENSMUSG00000024868	Dkk1	2.455358264	5.107038876	-1.056553496	0.000116564	0.004180377	DOWN
ENSMUSG00000041911	Dlx1	0.272545141	1.245979072	-2.19271273	3.51E-13	6.79E-11	DOWN

ENSMUSG00000023391	Dlx2	1.981183658	7.292231285	-1.879997693	6.98E-13	1.27E-10	DOWN
ENSMUSG00000001510	Dlx3	2.701416937	5.52282034	-1.031688874	0.000113371	0.004073502	DOWN
ENSMUSG00000029755	Dlx5	0.784226228	3.769782604	-2.265139531	1.06E-13	2.18E-11	DOWN
ENSMUSG00000090063	Dlx6os1	0.139610391	0.712151117	-2.350777083	9.22E-10	0.000000104	DOWN
ENSMUSG00000028707	Dmbx1	0.069796002	1.788192998	-4.679214241	2.37E-34	3.24E-31	DOWN
ENSMUSG00000042372	Dmrt3	0.251856568	1.492983331	-2.567523797	0.000000171	0.0000124	DOWN
ENSMUSG00000047143	Dmrta2	1.112401477	4.370422108	-1.974095061	3.08E-11	4.53E-09	DOWN
ENSMUSG00000085256	Dmrta2os	0.049848903	1.243727271	-4.640964609	0.00000481	0.000255904	DOWN
ENSMUSG00000036766	Dner	0.579917416	1.295556584	-1.159652657	0.000250346	0.008126054	DOWN
ENSMUSG00000078190	Dnm3os	0.157716315	1.407556607	-3.157789132	1.06E-26	6.55E-24	DOWN
ENSMUSG00000039716	Dock3	0.195421636	0.445185288	-1.187815717	0.0000956	0.003526488	DOWN
ENSMUSG00000027560	Dok5	1.736337149	4.957023302	-1.513426934	0.00000124	0.0000742	DOWN
ENSMUSG00000022838	Eaf2	0.019682074	0.163762829	-3.0566538	0.002009464	0.044132158	DOWN
ENSMUSG00000095105	Edaradd	0.304567059	0.911611914	-1.581659865	0.00000145	0.0000847	DOWN
ENSMUSG00000069049	Eif2s3y	0	1.644296275	#NAME?	3.01E-32	3.2E-29	DOWN
ENSMUSG00000039095	En2	0.034014005	4.121443247	-6.920876947	4.3E-39	9.15E-36	DOWN
ENSMUSG00000032446	Eomes	0.042140661	0.298012596	-2.822088468	0.0000137	0.000624794	DOWN
ENSMUSG00000075302	Erich2	0.029751746	0.293976974	-3.30465694	0.00041343	0.012218522	DOWN
ENSMUSG00000005503	Evx1	0.576568197	2.158614688	-1.904542578	1.17E-08	0.0000011	DOWN
ENSMUSG00000001815	Evx2	0.022550686	0.340092591	-3.914684344	0.00000215	0.000122772	DOWN
ENSMUSG00000096753	Fam181a	2.823272146	5.872328066	-1.056564366	0.000104953	0.003821215	DOWN
ENSMUSG00000029697	Fezf1	1.299319864	3.144286576	-1.274976079	0.0000563	0.002195005	DOWN
ENSMUSG00000021743	Fezf2	0.417996478	3.450597401	-3.045283465	5.66E-15	1.36E-12	DOWN
ENSMUSG00000022101	Fgf17	1.819706034	6.382469753	-1.810409388	3.09E-09	0.000000318	DOWN
ENSMUSG00000057967	Fgf18	1.369181599	4.008086477	-1.549599828	0.0000378	0.001578292	DOWN
ENSMUSG00000037225	Fgf2	0.027749092	0.266966457	-3.266146001	0.000000944	0.0000578	DOWN
ENSMUSG00000031074	Fgf3	0.172355632	1.299509127	-2.91450642	0.000000013	0.00000118	DOWN
ENSMUSG00000035455	Fignl1	2.824976407	11.2206946	-1.989851263	1.17E-18	3.94E-16	DOWN
ENSMUSG00000040181	Fmo1	0.163765698	0.701688564	-2.099197649	8.65E-09	0.000000828	DOWN
ENSMUSG00000035451	Foxa1	1.529377564	3.750500302	-1.294138443	0.00000519	0.00027244	DOWN
ENSMUSG00000037025	Foxa2	0.961199287	2.146762337	-1.159254999	0.000163244	0.005612732	DOWN
ENSMUSG00000059246	Foxb1	1.512891957	4.111791779	-1.442458247	0.000000447	0.0000295	DOWN

ENSMUSG00000070990	Foxe1	0.064405505	1.049281169	-4.026073497	1.06E-09	0.000000118	DOWN
ENSMUSG00000044518	Foxe3	0.277682958	3.104718606	-3.482951972	3.65E-08	0.00000308	DOWN
ENSMUSG00000038402	Foxf2	3.693622508	8.291908449	-1.166667759	0.00000522	0.000273003	DOWN
ENSMUSG00000048377	Foxi2	0.511096086	1.522122813	-1.57441832	0.000147133	0.005113801	DOWN
ENSMUSG00000055874	Foxi3	0	0.277206826	#NAME?	0.001268294	0.030629386	DOWN
ENSMUSG00000034227	Foxj1	0.894979534	2.24339731	-1.32575855	0.000118334	0.004228005	DOWN
ENSMUSG00000050397	Foxl2	0.533565409	2.824844334	-2.404434324	0.000000652	0.0000412	DOWN
ENSMUSG00000097072	Foxl2os	0.043558378	1.147515352	-4.7194194	2.53E-15	6.29E-13	DOWN
ENSMUSG00000070880	Gad1	0.070787217	0.35582404	-2.329603225	0.000000787	0.0000489	DOWN
ENSMUSG00000026787	Gad2	0.10049035	0.365777012	-1.863907436	0.000878105	0.022991586	DOWN
ENSMUSG00000044576	Gareml	0.763122514	2.154160106	-1.497138885	0.00000209	0.000119966	DOWN
ENSMUSG00000029638	Glcci1	1.23103957	2.658552151	-1.110763631	0.00000403	0.000218388	DOWN
ENSMUSG00000052942	Glis3	0.28712149	0.754095098	-1.393085155	0.00000463	0.000247587	DOWN
ENSMUSG00000078444	Gm10941	0.170021981	1.414651784	-3.0566538	0.002009464	0.044132158	DOWN
ENSMUSG00000080772	Gm12543	2.660452157	10.37564536	-1.963457709	0.000000297	0.0000203	DOWN
ENSMUSG00000090093	Gm14399	0.237857165	0.725603101	-1.60908514	0.000764908	0.020661142	DOWN
ENSMUSG00000082214	Gm15009	0.18701079	2.916071807	-3.962832832	0.000794618	0.021343233	DOWN
ENSMUSG00000097796	Gm16702	0	0.315092323	#NAME?	0.000667207	0.018491577	DOWN
ENSMUSG00000090639	Gm20425	0	0.785600834	#NAME?	1.42E-10	1.89E-08	DOWN
ENSMUSG00000092367	Gm20479	0	1.351043341	#NAME?	0.000667207	0.018491577	DOWN
ENSMUSG00000092541	Gm20537	0	1.533071504	#NAME?	1.25E-08	0.00000115	DOWN
ENSMUSG00000093593	Gm20683	0.776680388	1.884725943	-1.278961816	0.000306909	0.009651245	DOWN
ENSMUSG00000093485	Gm20708	0.473420406	1.617744975	-1.772790398	0.000219377	0.007250981	DOWN
ENSMUSG00000095134	Gm21857	6.318382743	20.16443838	-1.674185987	4.15E-11	5.93E-09	DOWN
ENSMUSG00000033852	Gm28042	0.871935023	2.709957579	-1.635977735	2.3E-09	0.00000024	DOWN
ENSMUSG00000099876	Gm29650	0	0.928007757	#NAME?	0.001268294	0.030629386	DOWN
ENSMUSG00000103436	Gm36995	0.294296496	0.776079438	-1.398933967	0.000213665	0.007091685	DOWN
ENSMUSG00000097156	Gm3764	0.293842909	0.970243643	-1.723301994	0.000000133	0.00000987	DOWN
ENSMUSG00000104155	Gm38103	0.278647135	2.028722117	-2.864060041	0.0000648	0.002475863	DOWN
ENSMUSG00000051537	Gm5124	0.772282643	1.575430021	-1.02854482	0.00173677	0.039167399	DOWN
ENSMUSG00000073010	Gm5127	0.225452731	0.916510395	-2.023326255	0.0000728	0.002762596	DOWN
ENSMUSG00000068284	Gm608	0.682250448	2.343892448	-1.780533029	1.33E-14	3.06E-12	DOWN

ENSMUSG00000074634	Gm7120	0.558959429	1.511389393	-1.435059927	0.00000953	0.000455157	DOWN
ENSMUSG00000048603	Gm9828	0.350143509	1.044920393	-1.577374789	0.0001104	0.004004302	DOWN
ENSMUSG00000031517	Gpm6a	2.309576927	5.779612697	-1.323344218	0.000000315	0.0000215	DOWN
ENSMUSG00000044197	Gpr146	0.045811171	0.264191471	-2.527812533	0.000000105	0.00000792	DOWN
ENSMUSG00000063856	Gpx1	32.83109757	98.59685381	-1.58647863	1.19E-13	2.43E-11	DOWN
ENSMUSG00000022286	Grhl2	0.640631946	1.586634954	-1.30840259	0.00000144	0.0000844	DOWN
ENSMUSG00000047171	Helt	0	1.227459211	#NAME?	1.25E-08	0.00000115	DOWN
ENSMUSG00000028946	Hes3	0.592399581	3.696450477	-2.64149806	1.63E-11	2.52E-09	DOWN
ENSMUSG00000023781	Hes7	0.133085199	2.59535672	-4.285510808	2.92E-14	6.42E-12	DOWN
ENSMUSG00000028572	Hook1	0.79922238	1.774428856	-1.150685844	0.00000328	0.000179864	DOWN
ENSMUSG00000086903	Hotair	0.120015516	1.154636582	-3.266146001	0.000000944	0.0000578	DOWN
ENSMUSG00000055408	Hottip	0.012891774	0.187620805	-3.863297158	0.001410364	0.033386757	DOWN
ENSMUSG00000086427	Hoxa11os	1.361512046	3.20452138	-1.234899148	0.000000574	0.0000368	DOWN
ENSMUSG00000085696	Hoxaas3	0.834833998	2.197107667	-1.396044309	0.0000114	0.000531829	DOWN
ENSMUSG00000049604	Hoxb13	0	1.305342727	#NAME?	1.17E-12	2.02E-10	DOWN
ENSMUSG00000001656	Hoxc11	1.102006806	5.141798157	-2.222139843	4.84E-12	8.07E-10	DOWN
ENSMUSG00000050328	Hoxc12	0	5.16099294	#NAME?	1.14E-20	4.76E-18	DOWN
ENSMUSG00000001655	Hoxc13	0.122295245	2.365611724	-4.273773077	1.48E-16	4.17E-14	DOWN
ENSMUSG00000001823	Hoxd12	2.755499553	8.127630417	-1.560520908	1.78E-09	0.000000192	DOWN
ENSMUSG00000001819	Hoxd13	3.393903096	9.328235935	-1.458658905	8.88E-09	0.000000846	DOWN
ENSMUSG00000029096	Htra3	0.311880939	0.715483131	-1.197922372	0.000990729	0.025264258	DOWN
ENSMUSG00000025491	Ifitm1	10.0091693	24.60829276	-1.297822328	6.73E-08	0.0000054	DOWN
ENSMUSG00000040612	Ildr2	4.010222433	9.040838624	-1.172774342	0.000000116	0.00000862	DOWN
ENSMUSG00000054667	Irs4	1.128505152	2.556118557	-1.179541747	0.00000754	0.000374987	DOWN
ENSMUSG00000060969	Irx1	3.551226881	10.89498561	-1.617274852	1.37E-10	1.84E-08	DOWN
ENSMUSG00000001504	Irx2	1.624919872	3.41678365	-1.072270322	0.0000351	0.001481267	DOWN
ENSMUSG00000051243	Islr2	3.014338668	6.269723811	-1.056560376	0.00000793	0.000389263	DOWN
ENSMUSG00000032243	Itga11	0.3927629	1.213723818	-1.62770961	0.00000235	0.000132719	DOWN
ENSMUSG00000047976	Kcna1	0.04556978	0.205351908	-2.171949057	0.001245444	0.030578852	DOWN
ENSMUSG00000018470	Kcnab3	0.254564162	1.018210612	-1.999934772	0.0000289	0.001244394	DOWN
ENSMUSG00000035355	Kcnh4	0.035334188	0.281710859	-2.995078503	0.000206996	0.006882244	DOWN
ENSMUSG00000056673	Kdm5d	0	1.054223122	#NAME?	1.61E-52	1.54E-48	DOWN

ENSMUSG00000010021	Kif19a	0.363898065	1.090829393	-1.583819195	0.00000575	0.000297726	DOWN
ENSMUSG00000014602	Kif1a	2.362845726	5.12541922	-1.117144575	0.000000637	0.0000404	DOWN
ENSMUSG00000022629	Kif21a	5.150125016	10.54773724	-1.034254179	0.00000344	0.000187663	DOWN
ENSMUSG00000032036	Kirrel3	0.2706139	0.557353626	-1.042357026	0.001564149	0.036309102	DOWN
ENSMUSG00000043929	Klhl15	0.204710861	0.421620401	-1.042357026	0.001564149	0.036309102	DOWN
ENSMUSG00000029595	Lhx5	1.902717292	5.214727623	-1.454530679	0.000000179	0.0000129	DOWN
ENSMUSG00000026890	Lhx6	0.4737422	1.522169714	-1.683955125	5.24E-09	0.000000515	DOWN
ENSMUSG00000096225	Lhx8	0.137178963	3.963034136	-4.852474237	9.39E-34	1.2E-30	DOWN
ENSMUSG00000030226	Lmo3	0.337216154	0.920588794	-1.448883232	0.00000229	0.000130039	DOWN
ENSMUSG00000045776	Lrtm1	0.049355845	2.070133243	-5.390358874	1.41E-35	2.45E-32	DOWN
ENSMUSG00000061080	Lsamp	0.14736787	0.396166952	-1.426686526	0.00000455	0.000244934	DOWN
ENSMUSG00000056947	Mab21l1	2.68116664	10.14864197	-1.920353893	9.71E-14	2.02E-11	DOWN
ENSMUSG00000059401	Mamld1	0.533632268	1.098833839	-1.042055433	0.001662604	0.037995868	DOWN
ENSMUSG00000046709	Mapk10	0.150914406	0.392367233	-1.37847404	0.0000137	0.000624794	DOWN
ENSMUSG00000053436	Mapk14	12.80178739	31.44865113	-1.296652886	1.89E-09	0.000000203	DOWN
ENSMUSG00000043557	Mdga1	0.416844454	0.979275139	-1.232205119	0.00000329	0.000179864	DOWN
ENSMUSG00000030544	Mesp1	0	0.638257599	#NAME?	0.000352487	0.010769073	DOWN
ENSMUSG00000030543	Mesp2	0.030976067	0.676248724	-4.448328084	0.000026	0.001131438	DOWN
ENSMUSG00000028114	Mettl14	1.70374974	3.470107075	-1.026266743	0.00000685	0.000349049	DOWN
ENSMUSG00000035158	Mitf	0.160230559	0.573969284	-1.840824213	0.00000046	0.0000303	DOWN
ENSMUSG00000061013	Mkx	0.617371485	1.271266226	-1.042055433	0.001662604	0.037995868	DOWN
ENSMUSG00000035805	Mlc1	0.341093787	1.085058701	-1.669532715	0.000214124	0.007094631	DOWN
ENSMUSG00000047002	Msgn1	0.212302579	10.59899087	-5.641661204	6.41E-20	2.45E-17	DOWN
ENSMUSG00000017837	Nkiras2	5.885387719	14.21480285	-1.272184724	9.07E-09	0.00000086	DOWN
ENSMUSG00000048528	Nkx1-2	0.479473371	1.76445224	-1.879697781	0.000000325	0.0000221	DOWN
ENSMUSG00000001496	Nkx2-1	1.062896577	3.161022011	-1.572389855	0.000000202	0.0000143	DOWN
ENSMUSG00000054160	Nkx2-4	0	1.016125165	#NAME?	3.94E-08	0.00000331	DOWN
ENSMUSG00000044186	Nkx2-6	0.212302579	1.656095761	-2.963592293	0.0000253	0.001110539	DOWN
ENSMUSG00000058669	Nkx2-9	0.6666838	3.367730947	-2.336702308	0.000000143	0.0000104	DOWN
ENSMUSG00000022061	Nkx3-1	1.695579154	4.09496947	-1.272074561	0.00000501	0.000264969	DOWN
ENSMUSG00000041309	Nkx6-2	0.317528482	2.559308073	-3.01079591	1.38E-14	3.14E-12	DOWN
ENSMUSG00000031302	Nlgn3	0.504952161	1.101649644	-1.125446861	0.0000473	0.001921796	DOWN

ENSMUSG00000026643	Nmt2	3.819639884	8.01537372	-1.069333161	0.00000171	0.000099	DOWN
ENSMUSG00000068302	Noto	0.05446858	1.302980116	-4.580246994	2.41E-10	3.12E-08	DOWN
ENSMUSG00000035528	Npffr2	0	0.495300328	#NAME?	0.000352487	0.010769073	DOWN
ENSMUSG00000019803	Nr2e1	1.779826116	6.979152994	-1.971315658	8.59E-15	2.01E-12	DOWN
ENSMUSG00000059857	Ntng1	0.119554898	0.305181484	-1.351994196	0.000179322	0.006078214	DOWN
ENSMUSG00000040258	Nxph4	1.689010719	3.550465364	-1.071829649	0.00141697	0.033501712	DOWN
ENSMUSG00000005917	Otx1	0.307872302	3.149730495	-3.354824405	2.7E-30	2.58E-27	DOWN
ENSMUSG00000021848	Otx2	1.232457918	7.003405322	-2.506518199	2E-23	1.04E-20	DOWN
ENSMUSG00000014030	Pax5	0.036788152	1.077554639	-4.872376021	6.1E-27	3.89E-24	DOWN
ENSMUSG00000031860	Pbx4	0.692533807	1.680537222	-1.278966092	0.0000748	0.002823934	DOWN
ENSMUSG00000036422	Pcdh8	1.213614994	3.473362859	-1.517022321	1.02E-10	1.39E-08	DOWN
ENSMUSG00000055421	Pcdh9	0.018441184	0.109593663	-2.571161222	0.00000945	0.000454781	DOWN
ENSMUSG00000104148	Pcdha2	0.024421904	0.152406426	-2.641676172	0.000421961	0.01239414	DOWN
ENSMUSG00000102697	Pcdhac2	0.112458334	0.393376526	-1.806520285	0.000864433	0.022771324	DOWN
ENSMUSG00000103037	Pcdhgb1	0.590486081	3.736976064	-2.661896366	1.15E-23	6.1E-21	DOWN
ENSMUSG00000038370	Pcp4l1	1.297756956	4.68407821	-1.851744945	4.29E-08	0.00000357	DOWN
ENSMUSG00000039278	Pcsk1n	0.494071516	1.998179936	-2.015894715	0.0000017	0.000099	DOWN
ENSMUSG00000027419	Pcsk2	0.157480076	0.454111491	-1.52787723	0.000252326	0.008135182	DOWN
ENSMUSG00000086171	Pcsk2os1	0.039199704	0.25817904	-2.719457213	0.001488068	0.034831857	DOWN
ENSMUSG00000005615	Pcyt1a	2.650729809	7.174411804	-1.436470934	7.17E-11	9.95E-09	DOWN
ENSMUSG00000036218	Pdzrn4	0.230453191	0.659102506	-1.516029111	0.001339119	0.032007731	DOWN
ENSMUSG00000024901	Peli3	0.826225837	1.80234792	-1.125269452	0.0000978	0.003594256	DOWN
ENSMUSG00000025359	Pmel	0.282599624	3.085921549	-3.448869937	5.39E-15	1.31E-12	DOWN
ENSMUSG00000090125	Pou3f1	0.766294756	2.87872647	-1.909459375	4.58E-10	5.66E-08	DOWN
ENSMUSG00000056854	Pou3f4	4.255474296	10.47609216	-1.299708814	0.000000073	0.00000578	DOWN
ENSMUSG00000068748	Ptprz1	0.435033842	1.262515913	-1.53710203	8.75E-10	9.98E-08	DOWN
ENSMUSG00000043587	Pxylp1	0.787689268	2.426439816	-1.623142552	4.63E-11	6.53E-09	DOWN
ENSMUSG00000020684	Rasl10b	0.976952887	2.15395301	-1.140625881	0.00000721	0.000363537	DOWN
ENSMUSG00000024518	Rax	0.108204336	4.088320111	-5.239677946	5.17E-21	2.2E-18	DOWN
ENSMUSG00000033565	Rbfox2	9.947739106	24.85871649	-1.321311232	5.34E-10	6.44E-08	DOWN
ENSMUSG00000046667	Rbm12b1	0.202640696	0.553208053	-1.448898221	0.000395595	0.01187467	DOWN
ENSMUSG00000027510	Rbm38	2.425233334	6.617528039	-1.448168846	1.67E-10	0.000000022	DOWN

ENSMUSG00000049353	Rd3	0	0.106166362	#NAME?	0.0000533	0.00210093	DOWN
ENSMUSG00000097348	Rmst	0.174532042	0.459114964	-1.395363535	0.0000886	0.003289973	DOWN
ENSMUSG00000108584	RP23-190H11.1	0	1.14763626	#NAME?	0.001268294	0.030629386	DOWN
ENSMUSG00000108434	RP23-291B1.2	0.187987882	0.645161654	-1.779021027	0.00179023	0.040146009	DOWN
ENSMUSG00000032215	Rsl24d1	12.11128095	28.38992201	-1.229027426	2.91E-08	0.0000025	DOWN
ENSMUSG00000051920	Rspo2	1.919524268	5.76281326	-1.586024471	1.73E-09	0.000000189	DOWN
ENSMUSG00000019880	Rspo3	3.670947143	7.870760053	-1.100350617	0.000018	0.000815049	DOWN
ENSMUSG00000060487	Samd5	0.940894316	2.2831959	-1.27895006	0.0002196	0.007250981	DOWN
ENSMUSG00000050132	Sarm1	0.401213846	0.965981688	-1.267624447	0.0000148	0.000675153	DOWN
ENSMUSG00000038331	Satb2	0.57241281	1.267137926	-1.146445705	0.00000851	0.000413477	DOWN
ENSMUSG00000034115	Scn11a	0	0.171590451	#NAME?	0.0000533	0.00210093	DOWN
ENSMUSG00000037541	Shank2	0.211205362	0.630606481	-1.578093534	0.000000104	0.00000788	DOWN
ENSMUSG00000002633	Shh	6.08910587	12.66511412	-1.056557775	0.0000068	0.000347461	DOWN
ENSMUSG00000062713	Sim2	0.557646517	1.466916093	-1.395363535	0.0000886	0.003289973	DOWN
ENSMUSG00000038805	Six3	0.343498274	1.316862475	-1.93872993	1.29E-10	1.74E-08	DOWN
ENSMUSG00000093460	Six3os1	0.383536278	0.935485696	-1.286352546	0.000000469	0.0000308	DOWN
ENSMUSG00000021099	Six6	0.195193374	1.311645143	-2.748401479	2.47E-10	3.18E-08	DOWN
ENSMUSG00000030500	Slc17a6	0.209054364	0.782674875	-1.904534976	0.0000321	0.001371229	DOWN
ENSMUSG00000020261	Slc36a1	0.828273851	3.061774941	-1.886188491	6.29E-13	1.16E-10	DOWN
ENSMUSG00000034224	Slc38a8	0.026317351	0.84853998	-5.010896463	1.26E-13	2.55E-11	DOWN
ENSMUSG00000024131	Slc3a1	0.052178397	0.461279499	-3.144116641	0.001187534	0.029383016	DOWN
ENSMUSG00000028360	Slc44a5	0.214712647	0.614065182	-1.515984631	0.0000229	0.001023741	DOWN
ENSMUSG00000036814	Slc6a20a	0.018280958	0.266052453	-3.863297158	0.001410364	0.033386757	DOWN
ENSMUSG00000097769	Snhg4	1.344545775	2.900612912	-1.109238907	0.0000252	0.001108979	DOWN
ENSMUSG00000056153	Socs6	1.470775556	3.932612192	-1.41891082	4.41E-10	5.49E-08	DOWN
ENSMUSG00000036169	Sostdc1	2.165709217	5.398400317	-1.317692413	0.0000122	0.000563309	DOWN
ENSMUSG00000096014	Sox1	2.690759201	6.956991358	-1.37045024	1.08E-08	0.00000102	DOWN
ENSMUSG00000075304	Sp5	1.576502186	4.680090002	-1.569809104	0.000000268	0.0000185	DOWN
ENSMUSG00000038453	Srcin1	0.598153138	1.401299076	-1.228178107	0.00000713	0.000360273	DOWN
ENSMUSG00000054679	Srsf12	0.315404557	0.845940728	-1.42335308	0.001489598	0.034831857	DOWN
ENSMUSG00000022044	Stmn4	1.945231538	4.191677063	-1.107585685	0.0000238	0.001048084	DOWN
ENSMUSG00000017548	Suz12	5.313797634	11.71815594	-1.140930363	0.000000199	0.0000141	DOWN

ENSMUSG00000053025	Sv2b	0.082514929	0.234861191	-1.509081262	0.000645307	0.018041272	DOWN
ENSMUSG00000062327	T	0.792014615	8.110116939	-3.356123759	3.26E-29	2.84E-26	DOWN
ENSMUSG00000028417	Tal2	0.36469163	5.478291895	-3.908977142	3.49E-29	2.86E-26	DOWN
ENSMUSG00000009097	Tbx1	2.337277947	5.49554905	-1.233434317	0.000033	0.001402479	DOWN
ENSMUSG00000030699	Tbx6	0.132003772	4.118421322	-4.963440373	2.54E-25	1.52E-22	DOWN
ENSMUSG00000055320	Tead1	0.700753183	2.486551094	-1.827167779	3.46E-15	8.5E-13	DOWN
ENSMUSG00000025215	Tlx1	0.045101372	1.360370364	-4.914684344	7.96E-13	1.43E-10	DOWN
ENSMUSG00000009470	Tnpo1	25.51242983	55.56900859	-1.123080192	0.000000104	0.00000788	DOWN
ENSMUSG00000074607	Tox2	1.345886115	3.852516923	-1.517244956	9.62E-09	0.000000907	DOWN
ENSMUSG00000021573	Tppp	0.040225752	0.271949745	-2.757148784	0.000165623	0.005674154	DOWN
ENSMUSG00000005892	Trh	0.130486298	6.92037257	-5.728879482	9E-28	6.89E-25	DOWN
ENSMUSG00000030523	Trpm1	0.034989056	0.408656707	-3.54591377	5.53E-19	1.93E-16	DOWN
ENSMUSG00000052387	Trpm3	0.060795596	0.25922577	-2.092170416	0.0000682	0.002596549	DOWN
ENSMUSG00000030276	Ttll3	0.229494655	0.496176751	-1.112393581	0.001741559	0.039167399	DOWN
ENSMUSG00000054134	Umodl1	0	0.134084575	#NAME?	0.000187	0.006282871	DOWN
ENSMUSG00000029640	Usp12	3.031095726	7.965798151	-1.393979508	7.99E-10	9.27E-08	DOWN
ENSMUSG00000046269	Usp27x	0.304455375	0.649912862	-1.094015518	0.002120812	0.046154172	DOWN
ENSMUSG00000068457	Uty	0	0.472159796	#NAME?	2.99E-27	2.12E-24	DOWN
ENSMUSG00000006270	Vax1	0.372149641	1.499699331	-2.010718539	0.000877812	0.022991586	DOWN
ENSMUSG00000027831	Veph1	0.200347455	0.501604646	-1.324046526	0.000907958	0.023632476	DOWN
ENSMUSG00000021239	Vsx2	0.078570996	0.541323943	-2.784423443	9.45E-08	0.00000727	DOWN
ENSMUSG00000023336	Wfdc1	0.992476831	4.954351052	-2.319590769	3.16E-09	0.000000323	DOWN
ENSMUSG00000036961	Wnt8b	0.588389997	4.72838078	-3.006501595	1.43E-21	6.52E-19	DOWN
ENSMUSG00000063659	Zbtb18	1.416980808	3.431243736	-1.275911392	1.26E-08	0.00000115	DOWN
ENSMUSG00000040433	Zbtb38	0.146100756	0.415840175	-1.509065502	0.000000685	0.0000432	DOWN
ENSMUSG00000079605	Zbtb9	1.871637508	11.81300828	-2.658003455	5.75E-24	3.24E-21	DOWN
ENSMUSG00000102976	Zc3h11a	3.071437706	6.544600067	-1.09139091	0.00000098	0.0000598	DOWN
ENSMUSG00000043456	Zfp536	0.315757718	0.664312321	-1.073043676	0.00018107	0.006126632	DOWN
ENSMUSG00000046311	Zfp62	0.362611515	0.820377569	-1.177863308	0.00000866	0.000418826	DOWN
ENSMUSG00000074165	Zfp788	0.303423392	0.755557671	-1.316209566	0.00000724	0.000363743	DOWN
ENSMUSG00000098022	Zfp82	0.17607364	0.712116454	-2.015934252	8.55E-10	0.000000098	DOWN
ENSMUSG00000079509	Zfx	0.733861055	3.63284033	-2.307519116	1.5E-21	6.66E-19	DOWN

ENSMUSG00000067860	Zic3	1.22506912	3.31613955	-1.43664157	1.47E-08	0.00000132	DOWN
ENSMUSG00000026621	01-Mar	0.371988683	0.042983526	3.113402999	0.0000285	0.001233709	UP
ENSMUSG00000097222	1010001N08Rik	1.585795353	0.371641359	2.093223632	0.000000531	0.0000346	UP
ENSMUSG00000026227	2810459M11Rik	0.516485863	0.130209759	1.987891287	0.00040416	0.012056178	UP
ENSMUSG00000097910	5033428I22Rik	2.390027291	0.981965331	1.283283097	0.000417566	0.012302771	UP
ENSMUSG00000052595	A1cf	0.204422736	0	Inf	0.000352487	0.010769073	UP
ENSMUSG00000097924	A730020E08Rik	0.411183735	0.106904188	1.943464818	0.000287197	0.009151591	UP
ENSMUSG00000057230	Aak1	3.256751407	0.896854995	1.860486948	5.2E-17	1.58E-14	UP
ENSMUSG00000026782	Abi2	4.308650492	1.176973193	1.872154613	7.11E-17	2.09E-14	UP
ENSMUSG00000032735	Ablim3	0.976307473	0.260351253	1.906876221	0.00000112	0.0000678	UP
ENSMUSG00000031972	Acta1	64.09974009	17.86699755	1.843021289	1.15E-16	3.28E-14	UP
ENSMUSG00000035783	Acta2	204.7450276	83.37296814	1.296176813	4.66E-10	5.71E-08	UP
ENSMUSG00000068614	Actc1	513.1682011	200.799789	1.353674022	6.21E-11	8.69E-09	UP
ENSMUSG00000059430	Actg2	3.03937632	1.212064451	1.326308898	0.000019	0.000858381	UP
ENSMUSG00000052374	Actn2	11.93788732	4.877403184	1.291362401	7.11E-09	0.000000691	UP
ENSMUSG00000049538	Adamts16	0.546312874	0.191669318	1.511108013	0.0000475	0.001921796	UP
ENSMUSG00000036545	Adamts2	1.565326218	0.661543501	1.242555417	0.00000265	0.000147809	UP
ENSMUSG00000066113	Adamtsl1	0.569872194	0.270123886	1.077017183	0.000223869	0.00736652	UP
ENSMUSG00000031486	Adgra2	17.96316568	7.961961574	1.173845807	2.12E-08	0.00000184	UP
ENSMUSG00000074207	Adh1	2.286385356	1.120299883	1.029183615	0.001323297	0.031725449	UP
ENSMUSG00000031448	Adprhl1	0.818283966	0.243145985	1.750778812	0.00000231	0.000131165	UP
ENSMUSG00000031489	Adrb3	0.599191111	0.095054003	2.656196939	5.99E-08	0.00000486	UP
ENSMUSG00000039313	AF529169	0.300414394	0.102014001	1.558186766	0.000543102	0.015523808	UP
ENSMUSG00000029369	Afm	0.37177426	0.073645108	2.335765296	0.001695242	0.038511961	UP
ENSMUSG00000017718	Afmid	0.926402116	0.433551874	1.095433928	0.002120812	0.046154172	UP
ENSMUSG00000054932	Afp	56.38715622	1.114191721	5.661297179	7.36E-95	1.41E-90	UP
ENSMUSG00000071551	Akr1c19	0.699351051	0.155846493	2.165891129	0.000538084	0.015427684	UP
ENSMUSG00000029368	Alb	8.971443985	0.219814699	5.350980444	7.14E-45	2.73E-41	UP
ENSMUSG00000075296	Aldh3b2	1.124590748	0.380092685	1.564976918	0.001570614	0.036371004	UP
ENSMUSG00000025701	Alox5	0.438833146	0.152122336	1.528440498	0.000378252	0.011461884	UP
ENSMUSG00000032845	Alpk2	1.388047705	0.50560813	1.456965583	0.000000262	0.0000182	UP
ENSMUSG00000038763	Alpk3	2.757223227	1.148883113	1.262984048	0.000000691	0.0000434	UP

ENSMUSG00000028356	Ambp	13.70195294	0.342900227	5.320448866	3.11E-44	8.5E-41	UP
ENSMUSG00000024803	Ankrd1	13.50513237	6.526009389	1.049234813	0.0000126	0.000581297	UP
ENSMUSG00000032083	Apoa1	7.143627558	0.126631844	5.817944719	2.42E-30	2.43E-27	UP
ENSMUSG00000005681	Apoa2	5.24098461	0.610120287	3.102672265	7.51E-16	2.06E-13	UP
ENSMUSG00000032080	Apoa4	0.364823511	0	Inf	0.001268294	0.030629386	UP
ENSMUSG00000020609	Apob	0.412377107	0.085065256	2.277322348	3.18E-10	4.03E-08	UP
ENSMUSG00000040694	Apobec2	6.078637133	2.56976964	1.24210886	0.0000801	0.003003734	UP
ENSMUSG00000040564	Apoc1	1.767534802	0.204236024	3.113429357	0.000700954	0.019315074	UP
ENSMUSG00000024391	Apom	6.417447414	0.556155057	3.528440498	9.33E-16	2.52E-13	UP
ENSMUSG00000053199	Arhgap20	0.433238397	0.215310369	1.008743315	0.001371342	0.032664898	UP
ENSMUSG00000037509	Arhgef4	0.463586553	0.223203268	1.054480571	0.000894278	0.023332867	UP
ENSMUSG00000074794	Arrdc3	8.791736721	3.909035904	1.169335348	4.86E-08	0.00000401	UP
ENSMUSG00000030996	Art1	1.061992234	0.22088335	2.265416636	0.000758987	0.020559219	UP
ENSMUSG00000028539	Artn	0.871510605	0.32260798	1.433736093	0.000448896	0.013124911	UP
ENSMUSG00000021200	Asb2	5.361892167	1.905466391	1.492598044	1.77E-09	0.000000192	UP
ENSMUSG00000085779	Atcayos	0.828147551	0.328641247	1.333374273	0.000384864	0.01160714	UP
ENSMUSG00000030730	Atp2a1	3.384676723	1.470796485	1.202420411	0.00000826	0.00040459	UP
ENSMUSG00000033632	AW554918	1.725065256	0.723820383	1.252947297	0.000000445	0.0000295	UP
ENSMUSG00000069920	B3gnt9	1.055237954	0.502012501	1.071773164	0.00134041	0.032007731	UP
ENSMUSG00000102478	BC085271	0.792680409	0.212738629	1.897657303	0.000877812	0.022991586	UP
ENSMUSG00000030046	Bmp10	2.734840913	0.641333197	2.092310921	4.09E-12	6.86E-10	UP
ENSMUSG00000031963	Bmper	2.888264103	1.36983576	1.07619974	0.000102395	0.003742307	UP
ENSMUSG00000025105	Bnc1	8.93909031	2.690732868	1.732128853	1.8E-13	3.55E-11	UP
ENSMUSG00000050334	C130071C03Rik	2.736863357	0.211179848	3.695979333	3.58E-29	2.86E-26	UP
ENSMUSG00000058914	C1qtnf3	7.292440021	2.119070707	1.782969888	5.68E-13	1.08E-10	UP
ENSMUSG00000061535	C1qtnf7	0.411028433	0.123734368	1.731991927	0.000610301	0.017213371	UP
ENSMUSG00000047990	C2cd4a	0.756864911	0	Inf	0.0000286	0.001237041	UP
ENSMUSG00000024164	C3	0.816004322	0.066125009	3.625308884	9.38E-13	1.65E-10	UP
ENSMUSG00000032246	Calml4	1.817655149	0.290816917	2.643895412	0.000165623	0.005674154	UP
ENSMUSG00000031883	Car7	1.633425493	0.415653638	1.974446905	0.000000089	0.0000069	UP
ENSMUSG00000026928	Card9	0.345060511	0.044857285	2.943435165	0.002009464	0.044132158	UP
ENSMUSG00000007655	Cav1	1.571064134	0.594138334	1.402871297	0.00000029	0.0000199	UP

ENSMUSG00000062694	Cav3	2.445197022	0.865664186	1.498071336	0.000993175	0.025292936	UP
ENSMUSG00000108789	Ccdc106	0.762700034	0	Inf	0.000352487	0.010769073	UP
ENSMUSG00000034031	Ccdc182	4.899449363	1.871762991	1.38822185	0.0000524	0.002095566	UP
ENSMUSG00000022665	Ccdc80	9.956647376	3.050612664	1.706561024	4.25E-14	9.26E-12	UP
ENSMUSG00000031962	Cdh15	4.996701674	1.981700819	1.334236912	0.000000754	0.000047	UP
ENSMUSG00000040420	Cdh18	0.15769836	0	Inf	0.0000533	0.00210093	UP
ENSMUSG00000090231	Cfb	0.3385872	0	Inf	7.04E-08	0.00000562	UP
ENSMUSG00000058952	Cfi	0.319400096	0	Inf	0.000352487	0.010769073	UP
ENSMUSG00000026251	Chrnd	0.236796695	0.024625626	3.265416636	0.000243751	0.007952416	UP
ENSMUSG00000026253	Chrng	0.781422084	0.147753756	2.40290722	9.38E-08	0.00000724	UP
ENSMUSG00000056643	Chst13	0.436970817	0	Inf	0.000667207	0.018491577	UP
ENSMUSG00000056025	Clca3a1	0.242291065	0.011998915	4.335765296	0.000026	0.001131438	UP
ENSMUSG00000001739	Cldn15	0.458161186	0.115010076	1.994094982	0.000857787	0.022658584	UP
ENSMUSG00000032452	Clstn2	3.34463336	1.52964138	1.128654614	0.00000508	0.000268094	UP
ENSMUSG00000001349	Cnn1	9.07475816	3.965371881	1.194403025	0.00000115	0.000069	UP
ENSMUSG00000022371	Col14a1	2.471389825	0.901593632	1.454773362	4.7E-09	0.000000466	UP
ENSMUSG00000031273	Col4a6	5.284479195	2.489083025	1.086146943	0.00000132	0.0000785	UP
ENSMUSG00000038591	Colec10	1.312254762	0.068234512	4.265402402	2.22E-17	6.98E-15	UP
ENSMUSG00000030785	Cox6a2	8.23230652	3.958516222	1.056336937	0.001605081	0.036990252	UP
ENSMUSG00000021999	Cpb2	0.653811192	0	Inf	0.0000533	0.00210093	UP
ENSMUSG00000031825	Crispld2	5.940725926	2.528332109	1.232453251	7.15E-08	0.00000568	UP
ENSMUSG00000042401	Crtac1	5.20018565	2.416903491	1.105403263	0.0000327	0.001390417	UP
ENSMUSG00000032060	Cryab	24.0265011	11.78042552	1.028234918	0.0000119	0.000552522	UP
ENSMUSG00000030470	Csrp3	35.75718847	14.79752582	1.27287733	4.53E-08	0.00000376	UP
ENSMUSG00000063415	Cyp26b1	5.102808316	2.24679296	1.183424262	0.00000014	0.0000102	UP
ENSMUSG00000052821	Cysltr1	0.417397588	0.13123342	1.669287097	0.000591283	0.016750983	UP
ENSMUSG00000026832	Cytip	0.073884699	0	Inf	0.001268294	0.030629386	UP
ENSMUSG00000019929	Dcn	16.37696243	7.509607392	1.124858404	0.00000109	0.0000658	UP
ENSMUSG00000054763	Defb42	0.359151073	0.018674923	4.265416636	0.0000458	0.001873815	UP
ENSMUSG00000026208	Des	22.02529719	9.191098522	1.260852278	4.9E-09	0.000000484	UP
ENSMUSG00000048138	Dmrt2	12.00274189	4.694341645	1.354369263	1.74E-08	0.00000153	UP
ENSMUSG00000087648	E130018N17Rik	0.505847725	0	Inf	0.001268294	0.030629386	UP

ENSMUSG00000022505	Emp2	6.474272724	3.093613311	1.065425262	0.0000114	0.000531829	UP
ENSMUSG00000087095	Emx2os	0.982654419	0.386345	1.346794411	0.000224545	0.007376089	UP
ENSMUSG00000028024	Enpep	0.596962511	0.248334305	1.265356764	0.001396679	0.033144731	UP
ENSMUSG00000001036	Epn2	4.532499794	2.020169774	1.165830413	0.000000226	0.0000159	UP
ENSMUSG00000006154	Eps8l1	0.602292635	0.244286338	1.301891434	0.00011117	0.004016997	UP
ENSMUSG00000037482	Erv3	0.356386737	0	Inf	0.00000454	0.000244801	UP
ENSMUSG00000030199	Etv6	7.912596976	2.551393865	1.632865648	1.51E-13	3.02E-11	UP
ENSMUSG00000021492	F12	0.647523766	0	Inf	0.000000411	0.0000275	UP
ENSMUSG00000027249	F2	0.436344471	0.01134439	5.265416636	8.47E-10	9.77E-08	UP
ENSMUSG00000049119	Fam110b	1.351533104	0.249877982	2.435301158	9.59E-17	2.78E-14	UP
ENSMUSG00000027955	Fam198b	1.843044271	0.7645207	1.269463256	0.000000063	0.00000509	UP
ENSMUSG00000044966	Fbxo48	1.179520012	0.447845786	1.39712596	0.000412811	0.012218522	UP
ENSMUSG00000028001	Fga	1.31508201	0	Inf	1.36E-21	6.34E-19	UP
ENSMUSG00000033831	Fgb	3.238738527	0.032860022	6.622954754	4.03E-35	6.43E-32	UP
ENSMUSG00000005320	Fgfr4	1.291895251	0.609283171	1.084304302	0.001729523	0.039105194	UP
ENSMUSG00000033860	Fgg	2.448100444	0.183726788	3.736028853	2.2E-15	5.63E-13	UP
ENSMUSG00000074971	Fibin	7.885930364	2.595690844	1.603162407	1.28E-09	0.000000141	UP
ENSMUSG00000022215	Fitm1	3.602877441	1.356684747	1.409064056	0.000588384	0.016693552	UP
ENSMUSG00000028088	Fmo5	0.22153267	0.052022841	2.090302381	0.000382556	0.011555737	UP
ENSMUSG00000021250	Fos	0.60321024	0.148572013	2.021498544	0.000134587	0.004729305	UP
ENSMUSG00000042812	Foxf1	15.42842702	6.739546675	1.194867523	0.000000195	0.0000139	UP
ENSMUSG00000038663	Fsd2	2.02405905	0.942540824	1.102624368	0.000251338	0.008130695	UP
ENSMUSG00000066705	Fxyd6	71.75468544	32.26121043	1.153272471	4.92E-08	0.00000404	UP
ENSMUSG00000020159	Gabrp	4.224094642	1.973409077	1.097952109	0.0000234	0.001037633	UP
ENSMUSG00000033066	Gas7	0.953866589	0.244889726	1.961655253	6.05E-12	9.91E-10	UP
ENSMUSG00000021944	Gata4	5.169334846	1.587553291	1.703173635	9.93E-13	1.73E-10	UP
ENSMUSG00000005836	Gata6	13.56893592	5.076207538	1.418484629	6.2E-10	7.37E-08	UP
ENSMUSG00000042390	Gatad2b	6.238756615	1.983093177	1.653506063	5.6E-14	1.19E-11	UP
ENSMUSG00000000394	Gcg	1.796803006	0.193304194	3.216487403	2.23E-09	0.000000235	UP
ENSMUSG00000072625	Gdf2	0.825939384	0	Inf	2.8E-11	4.18E-09	UP
ENSMUSG00000029275	Gfi1	0.12056631	0	Inf	0.000667207	0.018491577	UP
ENSMUSG00000048731	Ggnbp2	3.854637172	0.747397835	2.366646779	2.61E-20	1.02E-17	UP

ENSMUSG00000036855	Gjd4	0.429784638	0.070573102	2.606423588	0.001488068	0.034831857	UP
ENSMUSG00000084897	Gm14226	18.30112747	8.609191635	1.087982845	0.000000713	0.0000446	UP
ENSMUSG00000090015	Gm15446	2.111231017	0.993656392	1.087265494	0.000270771	0.008671451	UP
ENSMUSG00000091462	Gm17084	0.778031708	0.040455628	4.265416636	6.6E-12	1.07E-09	UP
ENSMUSG00000092470	Gm20518	0.995055586	0.427084552	1.220255406	0.001557178	0.036235125	UP
ENSMUSG00000092390	Gm20541	0.470949269	0	Inf	0.001268294	0.030629386	UP
ENSMUSG00000096056	Gm21986	1.082610179	0.236443798	2.19494465	3.86E-09	0.000000389	UP
ENSMUSG00000043903	Gm22	0.732868442	0.254061832	1.528374587	0.0000491	0.001975638	UP
ENSMUSG00000058447	Gm26920	2.636951603	0.342787885	2.943483067	6.22E-13	1.16E-10	UP
ENSMUSG00000099041	Gm28035	0.539729408	0.132936544	2.021498544	0.000134587	0.004729305	UP
ENSMUSG00000098530	Gm28051	12.98553139	0	Inf	2.54E-22	1.25E-19	UP
ENSMUSG00000099908	Gm28539	3.915995474	1.448494386	1.434825005	0.000000413	0.0000276	UP
ENSMUSG00000099913	Gm28551	0.957888603	0.019923088	5.587344731	8.6E-12	1.37E-09	UP
ENSMUSG00000099906	Gm28653	0.330855595	0.083053144	1.994094982	0.000857787	0.022658584	UP
ENSMUSG00000102805	Gm37240	1.541460018	0.545728794	1.4980414	0.00000146	0.0000852	UP
ENSMUSG00000104184	Gm37818	1.101668741	0.434593203	1.341952971	0.000949785	0.024349831	UP
ENSMUSG00000103408	Gm37933	1.043100265	0.127621491	3.030934637	0.001187534	0.029383016	UP
ENSMUSG00000105053	Gm43064	0.957279259	0.258835381	1.886905013	0.0000569	0.002211393	UP
ENSMUSG00000106339	Gm43489	1.212360923	0.126079238	3.265416636	0.000243751	0.007952416	UP
ENSMUSG00000091721	Gm5549	0.436819447	0	Inf	0.0000996	0.003654715	UP
ENSMUSG00000087153	Gm6483	1.107078132	0.54078176	1.033638646	0.0000545	0.002133611	UP
ENSMUSG00000079471	Gm7325	2.156007508	0.060598588	5.152934213	4.09E-09	0.00000041	UP
ENSMUSG00000090555	Gm8893	1.066223799	0	Inf	0.00000136	0.0000804	UP
ENSMUSG00000050675	Gp1ba	0.521910865	0.156797387	1.734901919	0.000305231	0.00961429	UP
ENSMUSG00000045281	Gpr20	1.712584551	0.613165821	1.481826031	0.000260181	0.008360269	UP
ENSMUSG00000072966	Gprasp2	2.443553308	0.283731013	3.106384819	4.52E-27	3.09E-24	UP
ENSMUSG00000004341	Gpx6	0.520499273	0	Inf	0.000187	0.006282871	UP
ENSMUSG00000046182	Gsg1l	1.626433512	0.781896783	1.05666177	0.000944723	0.024308304	UP
ENSMUSG00000034450	Gulo	2.923289092	0.414558553	2.817944719	8.31E-13	1.47E-10	UP
ENSMUSG00000025075	Habp2	1.039107994	0	Inf	6.52E-20	2.45E-17	UP
ENSMUSG00000037335	Hand1	19.80182367	7.783637355	1.347116902	7.4E-10	0.000000087	UP
ENSMUSG00000038403	Hfe2	3.353965892	0.740839519	2.178635054	2.04E-09	0.000000218	UP

ENSMUSG00000039377	Hlx	5.757608516	2.407691075	1.257819402	0.00000101	0.0000615	UP
ENSMUSG00000017950	Hnf4a	0.926834524	0.015179035	5.932159779	1.63E-24	9.48E-22	UP
ENSMUSG00000059005	Hnrnpa3	8.726847473	2.165330611	2.010873264	2.37E-18	7.83E-16	UP
ENSMUSG00000059325	Hopx	5.931217106	2.819815323	1.072727501	0.000111439	0.004019138	UP
ENSMUSG00000038236	Hoxa7	8.022884678	2.39034987	1.746899262	7.82E-14	1.65E-11	UP
ENSMUSG00000020875	Hoxb9	73.17120798	33.84021058	1.11253753	0.000000095	0.00000727	UP
ENSMUSG00000001661	Hoxc6	19.34569653	6.914032784	1.484413324	3.82E-11	5.5E-09	UP
ENSMUSG00000001657	Hoxc8	6.768255428	1.239450099	2.449083827	2.14E-20	8.54E-18	UP
ENSMUSG00000036139	Hoxc9	9.225096756	2.268419636	2.023876493	1.53E-15	3.97E-13	UP
ENSMUSG00000027102	Hoxd8	2.323076473	1.125758151	1.045139724	0.000125654	0.004481193	UP
ENSMUSG00000043342	Hoxd9	16.51369224	8.235314953	1.003766993	0.0000102	0.000484003	UP
ENSMUSG00000038239	Hrc	5.483395326	1.986890762	1.464556932	0.000000101	0.0000077	UP
ENSMUSG00000078915	Hsp25-ps1	13.57891467	6.323274722	1.102624368	0.000251338	0.008130695	UP
ENSMUSG00000038086	Hspb2	9.960528468	3.949731483	1.334467711	0.0000131	0.000602665	UP
ENSMUSG00000006221	Hspb7	10.39088928	4.069343881	1.352451023	1.62E-08	0.00000144	UP
ENSMUSG00000008590	Htr3b	0.474349615	0	Inf	0.00000835	0.000407994	UP
ENSMUSG00000001741	Il16	0.297805787	0.107448688	1.470723925	0.000945626	0.024308304	UP
ENSMUSG00000024578	Il17b	3.479405769	0.633220217	2.458061714	0.00000945	0.000454781	UP
ENSMUSG00000026072	Il1r1	0.729804269	0.267874956	1.445949885	0.00000953	0.000455157	UP
ENSMUSG00000060477	Irak2	1.27303637	0.518412141	1.296102227	0.00000739	0.000368693	UP
ENSMUSG00000031621	Isx	0.315968625	0	Inf	0.000187	0.006282871	UP
ENSMUSG00000037254	Itih2	11.21899719	0.621561503	4.173902764	5.54E-45	2.65E-41	UP
ENSMUSG00000059742	Kcnh7	0.115509633	0.030889033	1.902846556	0.00041851	0.012311644	UP
ENSMUSG00000049265	Kcnk3	1.686754161	0.623388381	1.436046549	0.00000953	0.000455157	UP
ENSMUSG00000043673	Kcns3	0.806769167	0.163709294	2.301019716	0.0000255	0.001116966	UP
ENSMUSG00000028357	Kif12	0.456101512	0.079054946	2.528427319	0.0000176	0.000799049	UP
ENSMUSG00000029195	Klb	0.315282525	0.106339198	1.567971712	0.001648005	0.03779754	UP
ENSMUSG00000026308	Klhl30	2.007091496	0.891676715	1.170513737	0.000353354	0.010775617	UP
ENSMUSG00000044938	Klhl31	0.481992848	0.086754708	2.473997783	0.000000898	0.0000556	UP
ENSMUSG00000075307	Klhl41	2.903741619	0.816847614	1.829774216	7.45E-08	0.00000587	UP
ENSMUSG00000026639	Lamb3	0.596856495	0.209496476	1.510458123	0.0000613	0.002357923	UP
ENSMUSG00000029683	Lmod2	1.949582142	0.538548484	1.856016804	0.00001	0.000477453	UP

ENSMUSG00000036832	Lpar3	3.175780921	0.88920629	1.836521333	3.55E-08	0.00000302	UP
ENSMUSG00000093445	Lrch4	1.695853957	0.771601887	1.136083357	0.0000474	0.001921796	UP
ENSMUSG00000060187	Lrrc10	2.107623243	0.745218351	1.499881889	0.000704966	0.019369883	UP
ENSMUSG00000043110	Lrrn4	6.124645978	2.207437988	1.472253545	3.48E-09	0.000000355	UP
ENSMUSG00000001089	Luzp1	7.395892403	3.58658626	1.044112907	0.000000624	0.0000398	UP
ENSMUSG00000050447	Lypd6	1.541314518	0.432733613	1.832610192	2.98E-11	4.43E-09	UP
ENSMUSG00000029454	Mapkapk5	5.657857679	2.696865156	1.068972498	0.00000457	0.000245102	UP
ENSMUSG00000005583	Mef2c	0.786680574	0.199691174	1.978007389	9.88E-16	2.63E-13	UP
ENSMUSG00000038244	Mical2	2.928862119	1.072394685	1.449504303	3.96E-10	4.96E-08	UP
ENSMUSG00000025355	Mmp19	0.670783938	0.146858279	2.191423546	0.0000565	0.002197419	UP
ENSMUSG00000029061	Mmp23	1.591613476	0.651533328	1.288579134	0.000178834	0.006072434	UP
ENSMUSG00000009350	Mpo	1.025103457	0.377033074	1.44300653	0.0000954	0.003526003	UP
ENSMUSG00000005611	Mrvi1	0.552119363	0.272212159	1.020248691	0.000605775	0.017136187	UP
ENSMUSG00000026100	Mstn	1.642911123	0.751301175	1.128791174	0.000147222	0.005113801	UP
ENSMUSG00000002100	Mybpc3	19.19969231	6.437638386	1.576479746	7.44E-13	1.34E-10	UP
ENSMUSG00000042451	Mybph	3.792840184	1.044748324	1.860123138	5.74E-10	6.87E-08	UP
ENSMUSG00000000435	Myf5	3.872239709	1.202110421	1.687598843	0.000000185	0.0000132	UP
ENSMUSG00000035923	Myf6	2.990383615	0.652066057	2.197240542	0.00000093	0.0000572	UP
ENSMUSG00000018830	Myh11	0.60810726	0.169898997	1.839648481	0.00000782	0.000385798	UP
ENSMUSG00000020908	Myh3	7.732162579	3.084858344	1.32566773	3.58E-09	0.000000363	UP
ENSMUSG00000053093	Myh7	28.15895618	8.041142875	1.808121387	2.01E-17	6.41E-15	UP
ENSMUSG00000061816	Myl1	5.06738508	2.25856525	1.165834872	0.00000532	0.000277446	UP
ENSMUSG00000013936	Myl2	64.41115355	14.5738721	2.143926295	1.14E-22	5.76E-20	UP
ENSMUSG00000059741	Myl3	36.96314322	12.4272533	1.57257998	5.83E-13	1.1E-10	UP
ENSMUSG00000061086	Myl4	27.50611195	11.80007295	1.220956448	7.06E-09	0.00000069	UP
ENSMUSG00000031698	Mylk3	3.460604691	1.59842391	1.114374081	0.00000558	0.000289718	UP
ENSMUSG00000030672	Mylpf	19.37962016	8.439238349	1.199355589	0.000000238	0.0000166	UP
ENSMUSG00000009471	Myod1	2.077871798	0.405167259	2.358517144	0.000000191	0.0000136	UP
ENSMUSG00000026459	Myog	19.6081281	3.852289038	2.347663957	1.55E-19	5.58E-17	UP
ENSMUSG00000024049	Myom1	5.857241834	2.643448391	1.147800301	0.000000477	0.0000312	UP
ENSMUSG00000068697	Myoz1	1.442016756	0.435382591	1.727732301	0.001847602	0.041191405	UP
ENSMUSG00000028116	Myoz2	0.649321311	0.158040339	2.038639717	0.0000355	0.00149082	UP

ENSMUSG00000036098	Myrf	17.7118567	6.723743246	1.397378916	3.36E-11	4.87E-09	UP
ENSMUSG00000002881	Nab1	2.553966744	1.220940628	1.064746693	0.0000398	0.001655407	UP
ENSMUSG00000026950	Neb	0.15746868	0.052405823	1.587265881	0.000029	0.00124684	UP
ENSMUSG00000038624	Nepn	1.937197067	0.121361655	3.996586158	1.6E-14	3.57E-12	UP
ENSMUSG00000047180	Neurl3	1.519075045	0.526606013	1.528397242	0.0000144	0.000658179	UP
ENSMUSG00000044312	Neurog3	4.143180954	1.991805896	1.05666177	0.000944723	0.024308304	UP
ENSMUSG00000069670	Nkain2	0.178986787	0	Inf	0.000352487	0.010769073	UP
ENSMUSG00000044220	Nkx2-3	2.422541098	0.652095578	1.893365799	0.000000567	0.0000365	UP
ENSMUSG00000029361	Nos1	0.18616792	0.071550582	1.37956909	0.002254856	0.048684039	UP
ENSMUSG00000089837	Npcd	0.455715535	0	Inf	0.001268294	0.030629386	UP
ENSMUSG00000041616	Nppa	71.04894404	24.02683295	1.564166713	3.43E-12	5.81E-10	UP
ENSMUSG00000029019	Nppb	9.017405832	2.56492865	1.813793758	0.000000138	0.0000102	UP
ENSMUSG00000048938	Nr1h5	0.756714262	0.125196683	2.595552298	0.00000272	0.000151315	UP
ENSMUSG00000026751	Nr5a1	0.662989865	0.051453511	3.687645388	1.12E-11	1.77E-09	UP
ENSMUSG00000026398	Nr5a2	0.333423979	0.127376232	1.388261764	0.001587386	0.036626539	UP
ENSMUSG00000052854	Nrk	2.433792751	1.167896392	1.059294027	0.0000032	0.000176608	UP
ENSMUSG00000072944	Nup62cl	2.623820111	1.307044015	1.005361087	0.001160344	0.029010106	UP
ENSMUSG00000061462	Obscn	0.463094463	0.225871071	1.035807002	0.0000577	0.002233321	UP
ENSMUSG00000009654	Oit3	2.465937728	0.357344461	2.786749037	2.34E-13	4.58E-11	UP
ENSMUSG00000022330	Osr2	2.410529839	0.762987533	1.659618899	0.0000281	0.001218855	UP
ENSMUSG00000020787	P2rx1	0.835410312	0.243869847	1.776373561	0.0000457	0.001873815	UP
ENSMUSG00000027508	Pag1	4.750731448	2.346259942	1.017786797	0.00000279	0.000154662	UP
ENSMUSG00000064225	Paqr9	0.848907784	0.239417865	1.826077037	0.000000109	0.00000819	UP
ENSMUSG00000073591	Pcdhb22	0.291637458	0.056426674	2.369726815	0.0000115	0.000532158	UP
ENSMUSG00000103144	Pcdhga1	0.492831816	0.189245817	1.380833982	0.000395595	0.01187467	UP
ENSMUSG00000103332	Pcdhga2	6.906375686	1.935809108	1.834992121	5.58E-14	1.19E-11	UP
ENSMUSG00000103677	Pcdhga4	0.283309392	0.022664135	3.643895412	0.0000103	0.0004875	UP
ENSMUSG00000103897	Pcdhga8	0.610835315	0.026468643	4.528427319	2.41E-10	3.12E-08	UP
ENSMUSG00000037652	Phc3	1.471816596	0.447926786	1.716263062	1.98E-12	3.39E-10	UP
ENSMUSG00000037578	Pkd2l1	0.245314655	0.026854715	3.191386089	0.00041343	0.012218522	UP
ENSMUSG00000024247	Pkdcc	61.30403786	28.11478927	1.124652869	6.69E-08	0.00000538	UP
ENSMUSG00000009646	Pla2g12b	0.557006296	0	Inf	0.0000533	0.00210093	UP

ENSMUSG00000061082	Plac1	1.882923763	0.854107936	1.140484284	0.000719254	0.019649685	UP
ENSMUSG00000051413	Plagl2	11.6521327	3.211160381	1.85942741	2.42E-17	7.48E-15	UP
ENSMUSG00000059481	Plg	1.878992476	0	Inf	1.76E-20	7.18E-18	UP
ENSMUSG00000038583	Pln	6.844555781	1.716456418	1.995523685	4.94E-13	9.45E-11	UP
ENSMUSG00000018217	Pmp22	20.26493235	8.674755718	1.224090322	0.000000014	0.00000127	UP
ENSMUSG00000022803	Popdc2	7.53399028	2.76616784	1.445525476	7.82E-10	9.13E-08	UP
ENSMUSG00000027750	Postn	24.11615313	8.871097531	1.442815284	6.06E-12	9.91E-10	UP
ENSMUSG00000024500	Ppp2r2b	2.433972712	0.902459335	1.431379163	0.000000133	0.00000987	UP
ENSMUSG00000025467	Prap1	1.067991407	0	Inf	0.001268294	0.030629386	UP
ENSMUSG00000018259	Prl8a2	0.996584765	0	Inf	0.000000227	0.000016	UP
ENSMUSG00000059456	Ptk2b	0.531223171	0.252093599	1.075358588	0.000889021	0.023227344	UP
ENSMUSG00000017311	Pyy	0.620071501	0	Inf	0.000352487	0.010769073	UP
ENSMUSG00000019832	Rab32	4.083327526	1.989664884	1.037219831	0.000397953	0.011908122	UP
ENSMUSG00000033220	Rac2	1.962830865	0.907242876	1.113375134	0.000734829	0.020018077	UP
ENSMUSG00000009281	Rarres2	1.35097137	0.589749504	1.195822897	0.000878797	0.022991586	UP
ENSMUSG00000026430	Rassf5	1.564426038	0.759252743	1.042981334	0.001118568	0.02813951	UP
ENSMUSG00000024990	Rbp4	15.77924948	1.592758378	3.308429256	5.84E-27	3.86E-24	UP
ENSMUSG00000023070	Rgn	0.486950986	0	Inf	0.000352487	0.010769073	UP
ENSMUSG00000070327	Rnf213	2.096952136	1.009628411	1.054469518	0.00000273	0.000151315	UP
ENSMUSG00000108145	RP24-378N16.1	0.190383747	0	Inf	0.001268294	0.030629386	UP
ENSMUSG00000006586	Runx1t1	1.484094135	0.583546374	1.346663389	0.000000056	0.00000457	UP
ENSMUSG00000021313	Ryr2	1.418286945	0.668764509	1.084579254	0.00000674	0.000344982	UP
ENSMUSG00000009614	Sardh	0.895406194	0.313304969	1.514974648	0.00000757	0.000375373	UP
ENSMUSG00000045954	Sdpr	5.753334054	2.315482694	1.313085265	0.000000341	0.0000231	UP
ENSMUSG00000026589	Sec16b	1.107799946	0.459033467	1.271026128	0.0000461	0.001881986	UP
ENSMUSG00000028883	Sema3a	3.26448756	1.36803146	1.254755136	1.64E-08	0.00000145	UP
ENSMUSG00000029173	Sepsecs	2.156159724	0.949849932	1.18269255	0.00000138	0.0000815	UP
ENSMUSG00000061947	Serpina10	0.240645649	0	Inf	0.0000533	0.00210093	UP
ENSMUSG00000066366	Serpina1a	1.53840034	0	Inf	6.42E-16	1.78E-13	UP
ENSMUSG00000071178	Serpina1b	5.860477394	0.156272607	5.228881375	4.21E-37	8.06E-34	UP
ENSMUSG00000071177	Serpina1d	0.863611475	0.044905553	4.265416636	0.0000458	0.001873815	UP
ENSMUSG00000072849	Serpina1e	1.292693457	0	Inf	8.24E-11	1.14E-08	UP

ENSMUSG00000060807	Serpina6	6.07763877	0.095764928	5.987869721	5.08E-33	6.08E-30	UP
ENSMUSG00000026715	Serpinc1	0.324701287	0.03493211	3.216487403	2.23E-09	0.000000235	UP
ENSMUSG00000022766	Serpind1	1.731951293	0.032748282	5.724835315	6.02E-22	2.88E-19	UP
ENSMUSG00000038224	Serpinf2	5.870260641	0.191774342	4.935942945	2.15E-42	5.15E-39	UP
ENSMUSG00000070436	Serpinh1	553.8795727	248.0762184	1.158788893	1.64E-08	0.00000145	UP
ENSMUSG00000018822	Sfrp5	3.548223871	1.64735759	1.106943284	0.000539089	0.015432136	UP
ENSMUSG00000030638	Sh3gl3	2.189394037	1.050094591	1.060012338	0.0000715	0.002717766	UP
ENSMUSG00000005202	Shbg	0.688096072	0	Inf	0.00000248	0.000139419	UP
ENSMUSG00000050010	Shisa3	6.943686293	3.009835511	1.206017125	0.000000143	0.0000104	UP
ENSMUSG00000036330	Slc18a1	0.263326915	0.066101733	1.994094982	0.000857787	0.022658584	UP
ENSMUSG00000023169	Slc38a1	37.3455146	16.5545776	1.173704778	1.56E-08	0.0000014	UP
ENSMUSG00000035699	Slc51a	1.203866973	0.352115671	1.773554644	0.001279284	0.030855881	UP
ENSMUSG00000031596	Slc7a2	0.914574264	0.360894913	1.341521515	0.000000253	0.0000176	UP
ENSMUSG00000054640	Slc8a1	13.25319353	6.166127972	1.103903301	0.00000124	0.0000743	UP
ENSMUSG00000078350	Smim1	1.676350602	0.659655197	1.345539887	1.24E-08	0.00000115	UP
ENSMUSG00000041476	Smpx	2.933146532	0.978132109	1.584347909	0.000000051	0.00000417	UP
ENSMUSG00000055027	Smyd1	5.825159358	2.8025289	1.055568268	0.00000216	0.000123365	UP
ENSMUSG00000027488	Snta1	2.290779707	1.144167157	1.001540891	0.00046987	0.01365476	UP
ENSMUSG00000045314	Sowahb	2.685544643	0.770457112	1.801428148	2.82E-09	0.000000292	UP
ENSMUSG00000043461	Sptssb	0.41394877	0.050645907	3.030934637	0.001187534	0.029383016	UP
ENSMUSG00000002007	Srpk3	1.09675924	0.399956831	1.455330663	0.000127341	0.004516112	UP
ENSMUSG00000079442	St6galnac4	3.1383069	1.517220154	1.048556002	0.00000517	0.00027178	UP
ENSMUSG00000035459	Stab2	0.438287072	0.061594353	2.831006118	6.46E-10	7.63E-08	UP
ENSMUSG00000040287	Stac3	0.86655215	0.233638171	1.891010578	0.0000306	0.001309809	UP
ENSMUSG00000048277	Syngr2	4.688832918	2.158625368	1.119115988	0.00000693	0.000351917	UP
ENSMUSG00000039376	Synpo2l	2.824364371	1.152957884	1.292586408	3.63E-08	0.00000308	UP
ENSMUSG00000053580	Tanc2	1.97970981	0.936326101	1.080205992	0.00000132	0.0000786	UP
ENSMUSG00000031965	Tbx20	5.561146517	2.373417135	1.228416667	8.51E-08	0.00000662	UP
ENSMUSG00000018263	Tbx5	6.493082532	2.663567883	1.285543498	4.06E-08	0.00000339	UP
ENSMUSG00000007877	Tcap	4.38116341	1.782929924	1.297064025	0.000563342	0.016030548	UP
ENSMUSG00000045680	Tcf21	11.81224365	4.913841231	1.265359871	0.00000209	0.000119966	UP
ENSMUSG00000028011	Tdo2	0.180870552	0.012539732	3.850379137	0.000794618	0.021343233	UP

ENSMUSG00000023990	Tfeb	0.548459563	0.156180085	1.812174755	0.00000142	0.0000835	UP
ENSMUSG00000029999	Tgfa	0.594096772	0.111552905	2.412969871	2.95E-08	0.00000252	UP
ENSMUSG00000021702	Thbs4	5.046538416	1.553771492	1.699519789	1.51E-10	1.99E-08	UP
ENSMUSG00000029390	Tmed2	17.22607034	1.453289126	3.567199973	8.59E-45	2.74E-41	UP
ENSMUSG00000034850	Tmem127	13.60241144	1.716989078	2.985909668	9.2E-35	1.36E-31	UP
ENSMUSG00000061451	Tmem151a	1.223118388	0.365119649	1.744122837	0.000000533	0.0000346	UP
ENSMUSG00000029569	Tmem168	5.876190029	2.681238765	1.131981354	0.000000554	0.0000358	UP
ENSMUSG00000084845	Tmem240	0.222054728	0.042764272	2.376437405	0.000313333	0.009821012	UP
ENSMUSG00000028364	Tnc	12.16163635	4.863367167	1.322309938	9.03E-10	0.000000102	UP
ENSMUSG00000091898	Tnnc1	49.03293886	21.9979934	1.156379303	0.000000105	0.00000788	UP
ENSMUSG00000017300	Tnnc2	13.76917979	6.259990017	1.137210361	0.0000444	0.001826887	UP
ENSMUSG00000026418	Tnni1	19.69682494	7.694339947	1.356093615	4.79E-10	5.81E-08	UP
ENSMUSG00000031097	Tnni2	1.487545588	0.501157049	1.569599202	0.0000494	0.001984147	UP
ENSMUSG00000035458	Tnni3	7.852959965	3.554327439	1.143659948	0.00000205	0.000118436	UP
ENSMUSG00000026414	Tnnt2	15.05004038	6.870473175	1.13128599	8.37E-08	0.00000654	UP
ENSMUSG00000061723	Tnnt3	1.929690286	0.752571081	1.358469558	0.000134283	0.004729305	UP
ENSMUSG00000026848	Tor1b	4.782386684	1.966310841	1.282239379	7.23E-09	0.0000007	UP
ENSMUSG00000019787	Trdn	0.280024919	0.055470396	2.335765296	0.001695242	0.038511961	UP
ENSMUSG00000032554	Trf	9.154640028	2.10326669	2.121871366	8.67E-19	2.97E-16	UP
ENSMUSG00000024457	Trim26	4.880967804	1.692041842	1.528401991	1.48E-11	2.3E-09	UP
ENSMUSG00000028834	Trim63	1.52156837	0.645617343	1.236807922	0.0000779	0.002931878	UP
ENSMUSG00000070332	Trim80	0.342375735	0	Inf	0.001268294	0.030629386	UP
ENSMUSG00000031997	Trpc6	1.152587732	0.496012725	1.216427531	0.00228741	0.049220428	UP
ENSMUSG00000047907	Tshz2	3.287254364	1.211314913	1.440309115	2.58E-10	3.29E-08	UP
ENSMUSG00000051747	Ttn	0.621056904	0.252213225	1.300081535	2.16E-09	0.00000023	UP
ENSMUSG00000061808	Ttr	6.764945325	0.205408832	5.041508157	7.32E-24	4.01E-21	UP
ENSMUSG00000029260	Ugt2b34	0.260159846	0.010020544	4.6983655	0.000000908	0.0000561	UP
ENSMUSG00000107705	Umad1	2.936509992	0	Inf	4.24E-47	2.71E-43	UP
ENSMUSG00000018845	Unc45b	6.569873601	2.926421266	1.166728151	0.000000573	0.0000368	UP
ENSMUSG00000042985	Upk3b	17.69989177	6.189060143	1.515948292	1.37E-11	2.15E-09	UP
ENSMUSG00000037568	Vash2	14.37554278	5.2576521	1.451125842	2.24E-11	3.41E-09	UP
ENSMUSG00000091243	Vgll3	2.283074372	1.09296463	1.062731142	0.000509216	0.014642633	UP

ENSMUSG00000073131	Vma21	5.195504307	1.914474292	1.440315505	4.71E-10	5.74E-08	UP
ENSMUSG00000050666	Vstm4	2.180911385	0.864042791	1.335756485	0.0000592	0.002279811	UP
ENSMUSG00000005124	Wisp1	5.146912214	1.915976426	1.425627366	1.02E-09	0.000000114	UP
ENSMUSG00000029671	Wnt16	2.034576129	0.866630167	1.231239901	0.0000105	0.000494011	UP
ENSMUSG00000010797	Wnt2	18.31158253	4.94138205	1.889769969	2.26E-15	5.7E-13	UP
ENSMUSG00000016458	Wt1	1.80126563	0.508639387	1.82429586	8.47E-12	1.36E-09	UP
ENSMUSG00000057836	Xlr3a	0.742550223	0.231676568	1.680376472	0.00000764	0.000377934	UP
ENSMUSG00000073125	Xlr3b	0.899556105	0.43657573	1.042981334	0.001118568	0.02813951	UP
ENSMUSG00000037638	Zbtb42	0.963366602	0.246078325	1.96896732	2.22E-08	0.00000192	UP
ENSMUSG00000044646	Zbtb7c	1.227535571	0.410342797	1.580863297	0.00000254	0.000142919	UP
ENSMUSG00000047003	Zfp41	13.67290171	5.935211944	1.203947995	2.07E-08	0.00000181	UP
ENSMUSG00000045639	Zfp629	11.49088258	5.46630037	1.071852968	0.000000438	0.0000291	UP
ENSMUSG00000022306	Zfpm2	1.476389737	0.7299615	1.016181334	0.000943825	0.024308304	UP
ENSMUSG00000002266	Zim1	6.417259369	2.987965016	1.102794038	0.000000354	0.0000239	UP

Table S3-overlapped DEGs

gene_id	gene_name
ENSMUSG00000026621	01-Mar
ENSMUSG00000079502	1700101E01Rik
ENSMUSG00000052419	2610001J05Rik
ENSMUSG00000041789	2700046A07Rik
ENSMUSG00000026227	2810459M11Rik
ENSMUSG00000059920	4930453N24Rik
ENSMUSG00000085488	4930557F10Rik
ENSMUSG00000052595	A1cf
ENSMUSG00000048636	A730049H05Rik
ENSMUSG00000026782	Abi2
ENSMUSG00000026842	Abl1
ENSMUSG00000037872	Ackr1
ENSMUSG00000059430	Actg2
ENSMUSG00000036545	Adamts2
ENSMUSG00000039313	AF529169
ENSMUSG00000029369	Afm
ENSMUSG00000054932	Afp
ENSMUSG00000093738	AI606473
ENSMUSG00000071551	Akr1c19
ENSMUSG00000029368	Alb
ENSMUSG00000053279	Aldh1a1
ENSMUSG00000015134	Aldh1a3
ENSMUSG00000075296	Aldh3b2
ENSMUSG00000032845	Alpk2
ENSMUSG00000028356	Ambp
ENSMUSG00000032083	Apoa1
ENSMUSG00000005681	Apoa2
ENSMUSG00000020609	Apob
ENSMUSG00000040564	Apoc1
ENSMUSG00000024391	Apom
ENSMUSG00000037509	Arhgef4
ENSMUSG00000035277	Arx
ENSMUSG00000021200	Asb2
ENSMUSG00000059995	Atxn7l3
ENSMUSG00000026432	Avpr1b
ENSMUSG00000097428	AW047730
ENSMUSG00000069920	B3gnt9
ENSMUSG00000061132	Blnk
ENSMUSG00000025105	Bnc1
ENSMUSG00000106775	C130093G08Rik
ENSMUSG00000058914	C1qtnf3
ENSMUSG00000024164	C3
ENSMUSG00000034031	Ccdc182
ENSMUSG00000049305	Ccdc71
ENSMUSG00000031962	Cdh15
ENSMUSG00000036510	Cdh8
ENSMUSG00000029646	Cdx2
ENSMUSG00000031326	Cdx4
ENSMUSG00000038192	Cer1
ENSMUSG00000090231	Cfb
ENSMUSG00000058952	Cfi

ENSMUSG00000031273	Col4a6
ENSMUSG00000038591	Colec10
ENSMUSG00000030785	Cox6a2
ENSMUSG00000039714	Cplx3
ENSMUSG00000030905	Crym
ENSMUSG00000030470	Csrp3
ENSMUSG00000024987	Cyp26a1
ENSMUSG00000063415	Cyp26b1
ENSMUSG00000062432	Cyp26c1
ENSMUSG00000026832	Cytip
ENSMUSG00000069045	Ddx3y
ENSMUSG00000090063	Dlx6os1
ENSMUSG00000028707	Dmbx1
ENSMUSG00000048138	Dmrt2
ENSMUSG00000042372	Dmrt3
ENSMUSG00000047143	Dmrta2
ENSMUSG00000085256	Dmrta2os
ENSMUSG00000069049	Eif2s3y
ENSMUSG00000032446	Eomes
ENSMUSG00000001036	Epn2
ENSMUSG00000006154	Eps8l1
ENSMUSG00000030199	Etv6
ENSMUSG00000021492	F12
ENSMUSG00000027249	F2
ENSMUSG00000044966	Fbxo48
ENSMUSG00000029697	Fezf1
ENSMUSG00000021743	Fezf2
ENSMUSG00000028001	Fga
ENSMUSG00000033831	Fgb
ENSMUSG00000022101	Fgf17
ENSMUSG00000033860	Fgg
ENSMUSG00000021250	Fos
ENSMUSG00000044518	Foxe3
ENSMUSG00000048377	Foxi2
ENSMUSG00000034227	Foxj1
ENSMUSG00000070880	Gad1
ENSMUSG00000044576	Gareml
ENSMUSG00000033066	Gas7
ENSMUSG00000021944	Gata4
ENSMUSG00000005836	Gata6
ENSMUSG00000072625	Gdf2
ENSMUSG00000090015	Gm15446
ENSMUSG00000090639	Gm20425
ENSMUSG00000092470	Gm20518
ENSMUSG00000093593	Gm20683
ENSMUSG00000095134	Gm21857
ENSMUSG00000096056	Gm21986
ENSMUSG00000098530	Gm28051
ENSMUSG00000097156	Gm3764
ENSMUSG00000104184	Gm37818
ENSMUSG00000105053	Gm43064
ENSMUSG00000091721	Gm5549

ENSMUSG00000074634	Gm7120
ENSMUSG00000090555	Gm8893
ENSMUSG00000072966	Gprasp2
ENSMUSG00000046182	Gsg1l
ENSMUSG00000034450	Gulo
ENSMUSG00000025075	Habp2
ENSMUSG00000037335	Hand1
ENSMUSG00000023781	Hes7
ENSMUSG00000017950	Hnf4a
ENSMUSG00000059005	Hnrnpa3
ENSMUSG00000086903	Hotair
ENSMUSG00000055408	Hottip
ENSMUSG00000086427	Hoxa11os
ENSMUSG00000038236	Hoxa7
ENSMUSG00000085696	Hoxaas3
ENSMUSG00000049604	Hoxb13
ENSMUSG00000020875	Hoxb9
ENSMUSG00000001656	Hoxc11
ENSMUSG00000050328	Hoxc12
ENSMUSG00000001655	Hoxc13
ENSMUSG00000001661	Hoxc6
ENSMUSG00000001657	Hoxc8
ENSMUSG00000036139	Hoxc9
ENSMUSG00000001823	Hoxd12
ENSMUSG00000001819	Hoxd13
ENSMUSG00000027102	Hoxd8
ENSMUSG00000038239	Hrc
ENSMUSG00000025491	Ifitm1
ENSMUSG00000026072	Il1r1
ENSMUSG00000054667	Irs4
ENSMUSG00000037254	Itih2
ENSMUSG00000059742	Kcnh7
ENSMUSG00000056673	Kdm5d
ENSMUSG00000026308	Klhl30
ENSMUSG00000044938	Klhl31
ENSMUSG00000026639	Lamb3
ENSMUSG00000026890	Lhx6
ENSMUSG00000096225	Lhx8
ENSMUSG00000036832	Lpar3
ENSMUSG00000043110	Lrrn4
ENSMUSG00000001089	Luzp1
ENSMUSG00000005583	Mef2c
ENSMUSG00000030544	Mesp1
ENSMUSG00000038244	Mical2
ENSMUSG00000025355	Mmp19
ENSMUSG00000029061	Mmp23
ENSMUSG00000047002	Msgn1
ENSMUSG00000002100	Mybpc3
ENSMUSG00000020908	Myh3
ENSMUSG00000053093	Myh7
ENSMUSG00000061086	Myl4
ENSMUSG00000030672	Mylpf

ENSMUSG00000024049	Myom1
ENSMUSG00000002881	Nab1
ENSMUSG00000038624	Nepn
ENSMUSG00000069670	Nkain2
ENSMUSG00000017837	Nkiras2
ENSMUSG00000048528	Nkx1-2
ENSMUSG00000001496	Nkx2-1
ENSMUSG00000054160	Nkx2-4
ENSMUSG00000044186	Nkx2-6
ENSMUSG00000022061	Nkx3-1
ENSMUSG00000029361	Nos1
ENSMUSG00000068302	Noto
ENSMUSG00000048938	Nr1h5
ENSMUSG00000019803	Nr2e1
ENSMUSG00000026398	Nr5a2
ENSMUSG00000052854	Nrk
ENSMUSG00000040258	Nxph4
ENSMUSG00000061462	Obscn
ENSMUSG00000009654	Oit3
ENSMUSG00000005917	Otx1
ENSMUSG00000021848	Otx2
ENSMUSG00000064225	Paqr9
ENSMUSG00000073591	Pcdhb22
ENSMUSG00000103144	Pcdhga1
ENSMUSG00000103897	Pcdhga8
ENSMUSG00000103037	Pcdhgb1
ENSMUSG00000005615	Pcyt1a
ENSMUSG00000024901	Peli3
ENSMUSG00000009646	Pla2g12b
ENSMUSG00000051413	Plagl2
ENSMUSG00000059481	Plg
ENSMUSG00000027750	Postn
ENSMUSG00000059456	Ptk2b
ENSMUSG00000019832	Rab32
ENSMUSG00000009281	Rarres2
ENSMUSG00000024518	Rax
ENSMUSG00000046667	Rbm12b1
ENSMUSG00000024990	Rbp4
ENSMUSG00000023070	Rgn
ENSMUSG00000006586	Runx1t1
ENSMUSG00000021313	Ryr2
ENSMUSG00000009614	Sardh
ENSMUSG00000029173	Sepsecs
ENSMUSG00000061947	Serpina10
ENSMUSG00000066366	Serpina1a
ENSMUSG00000071178	Serpina1b
ENSMUSG00000071177	Serpina1d
ENSMUSG00000072849	Serpina1e
ENSMUSG00000060807	Serpina6
ENSMUSG00000026715	Serpinc1
ENSMUSG00000022766	Serpind1
ENSMUSG00000038224	Serpinf2

ENSMUSG00000070436	Serpinh1
ENSMUSG00000018822	Sfrp5
ENSMUSG00000005202	Shbg
ENSMUSG00000062713	Sim2
ENSMUSG00000038805	Six3
ENSMUSG00000093460	Six3os1
ENSMUSG00000021099	Six6
ENSMUSG00000020261	Slc36a1
ENSMUSG00000023169	Slc38a1
ENSMUSG00000034224	Slc38a8
ENSMUSG00000097769	Snhg4
ENSMUSG00000075304	Sp5
ENSMUSG00000035459	Stab2
ENSMUSG00000017548	Suz12
ENSMUSG00000053025	Sv2b
ENSMUSG00000062327	T
ENSMUSG00000009097	Tbx1
ENSMUSG00000031965	Tbx20
ENSMUSG00000030699	Tbx6
ENSMUSG00000028011	Tdo2
ENSMUSG00000055320	Tead1
ENSMUSG00000023990	Tfeb
ENSMUSG00000029999	Tgfa
ENSMUSG00000021702	Thbs4
ENSMUSG00000034850	Tmem127
ENSMUSG00000029569	Tmem168
ENSMUSG00000028364	Tnc
ENSMUSG00000017300	Tnnc2
ENSMUSG00000032554	Trf
ENSMUSG00000024457	Trim26
ENSMUSG00000030523	Trpm1
ENSMUSG00000052387	Trpm3
ENSMUSG00000061808	Ttr
ENSMUSG00000029260	Ugt2b34
ENSMUSG00000018845	Unc45b
ENSMUSG00000046269	Usp27x
ENSMUSG00000068457	Uty
ENSMUSG00000006270	Vax1
ENSMUSG00000050666	Vstm4
ENSMUSG00000029671	Wnt16
ENSMUSG00000010797	Wnt2
ENSMUSG00000036961	Wnt8b
ENSMUSG00000057836	Xlr3a
ENSMUSG00000073125	Xlr3b
ENSMUSG00000037638	Zbtb42
ENSMUSG00000047003	Zfp41
ENSMUSG00000046311	Zfp62
ENSMUSG00000098022	Zfp82
ENSMUSG00000022306	Zfpm2

Table S4. 24 downregulated and 11 upregulated Homeobox genes in *Ube3d*$^{-/-}$ mice at E10.5. The genes highlighted in red are among the top 30 pathway.

Gene name	Log2FC (vs WT)	p_value	q_value	Up/down
Hoxa5	-1.824655347	1.60E-13	2.44E-11	DOWN
Hoxa6	-1.906457598	1.50E-06	9.19E-05	DOWN
Hoxa9	-1.768991881	7.88E-13	1.09E-10	DOWN
Hoxa10	-1.867918238	9.97E-15	1.65E-12	DOWN
Hoxa11	-1.58023	1.81E-10	2.00E-08	DOWN
Hoxa13	-1.998732667	1.91E-06	0.000113586	DOWN
Hoxb5	-1.618537088	6.14E-11	7.13E-09	DOWN
Hoxb6	-2.40368222	3.31E-19	9.57E-17	DOWN
Hoxb7	-2.025417063	8.08E-11	9.32E-09	DOWN
Hoxb8	-2.70174496	1.07E-22	3.82E-20	DOWN
Hoxc10	-7.113478502	5.12E-69	2.48E-65	DOWN
Hoxd1	-2.095515329	2.04E-09	1.97E-07	DOWN
Hoxd10	-2.474942494	8.48E-19	2.28E-16	DOWN
Hoxd11	-2.19639009	5.28E-18	1.28E-15	DOWN
Alx1	-1.693896105	3.59E-11	4.26E-09	DOWN
Alx3	-1.571407454	4.23E-11	4.99E-09	DOWN
Emx2	-1.124200335	5.48E-06	0.000298875	DOWN
Lhx2	-1.608237327	1.42E-12	1.95E-10	DOWN
Meox2	-1.212556648	5.69E-06	0.000306997	DOWN
Meox1	-2.097452361	2.63E-20	8.36E-18	DOWN
Mnx1	-1.879437671	1.29E-05	0.000643992	DOWN
Vax2os	-3.015976487	0.002009464	0.041593325	DOWN
Hoxb5os	-4.515879041	8.01E-37	7.76E-34	DOWN
Hoxd3os1	-5.109130796	2.06E-14	3.30E-12	DOWN
Phox2a	1.035452	0.001212	0.027791	UP
Hmx2	2.113516	0.00082	0.020383	UP
Prox2	1.531579	0.000675	0.017434	UP
Hnf1a	2.534976	0.000422	0.011746	UP
Nkx2-5	1.074782	0.000187	0.006204	UP
Gsx1	1.601554	5.94E-06	0.000319	UP
Hoxa2	1.257986	1.99E-07	1.51E-05	UP
Gbx1	1.935471	3.05E-10	3.25E-08	UP
Hoxb3os	2.057982	3.4E-12	4.46E-10	UP
Msx3	2.090027	1.41E-17	3.33E-15	UP
Phox2b	2.78416	6.9E-26	2.97E-23	UP

Table S5. All primers used in this study.

Category	Name	Sequence
Primers for plasmids construction	EcoRI-UBE3D-F	5’-gaattcatggcggcttctgcggcggag-3’
	UBE3D-NotI-R	5’-gcggccgcttacatcttcaaaaggccac-3’
	UBE3D-N-F	5'-caaggacgacgatgacaagcttatggccat-3’
	UBE3D-N-NotI-R	5'-ttgcggccgcttactccactggggatagtt-3’
	HindIII-UBE3D-C-F	5'-ccaagcttatccccagtggagatgtgct-3’
	UBE3D-C-R	5'-atccccgcggccgcttacatcttca-3’
	UBE3D-dm-F	5’-ctgccctctgcagccagcttggagctgctgt-3’
	UBE3D-dm-R	5’-acagcagctccaagctggctgcagagggcag-3’
	HindIII-CPSF3-F	5’-ccaagcttcccaagctggctagttaagct-3’
	CPSF3-NotI-R	5’-ttgcggccgcatcagcgggtttaaactcaa-3’
	CPSF3-H73A-F	5’-ttaattagtcatttcgctttggatcactgtgg-3’
	CPSF3-H73A-R	5’-ccacagtgatccaaagcgaaatgactaattaa-3’
	CPSF3-dm-F	5’-agtcatttccatttgaaagcctgtggagctctgcc-3’
	CPSF3-dm-R	5’-ggcagagctccacaggctttcaaatggaaatgact-3’
Primers for qRT-PCR in mouse	*Gapdh*-F	5’-tggagaaacctgccaagtatg-3’
	Gapdh-R	5’-ggagacaacctggtcctcag-3’
	Ube3d-F	5’-cgatggtttacatctgcgctt-3’
	Ube3d-R	5’-tcctgtcctttatcgtgacttca-3’
	Cpsf3-F	5’-gtgaatgccatgaacgacaa-3’
	Cpsf3-R	5’-aacagctctctggataagcc-3’
	Hoxa5-F	5’-tcagccccagatctacccct-3’
	Hoxa5-R	5’-gccttctgggccacctatatt-3’
	Hoxa9-F	5’-aatgccgagaatgagagcgg-3’
	Hoxa9-R	5’-tttgtgtaggggcatcgctt-3’
	Hoxa10-F	5’-cagccccttcagaaaacagtaa-3’
	Hoxa10-R	5’-tctcgagtaaggtacatgttga-3’
	Hoxa11-F	5’-gagtcgtcttccggccac-3’
	Hoxa11-R	5’-tataagggcagcgctttttgc-3’
	Hoxa13-F	5’-gccaaatgtactgccccaaa-3’
	Hoxa13-R	5’-gagctggcgtctgaaggatg-3’
	Hoxc10-F	5’-gacacctcggataacgaagc-3’
	Hoxc10-R	5’-tccttccgctctttgctgtc-3’
	Hoxd10-F	5’-tgaggtttccgtgtccagtc-3’
	Hoxd10-R	5’-agccaattgctggttggagt-3’
	Hoxd11-F	5’-ggcgggggccgaaaag-3’
	Hoxd11-R	5’-cggatctggtacttggtgtagg-3’
	Emx2-F	5’-tccaagggaacgacacaagtc-3’
	Emx2-R	5’-tagccttaaaagctgggacgg-3’
	Lhx2-F	5’-tgcaagctcaacctggagtc-3’

	Lhx2-R	5'-cagagaaccgcctgtagtagt-3'
	Mnx1-F	5'-gtacctgtctcgacccaagc-3'
	Mnx1-R	5'-ttggcctttttgctgcgttt-3'
	Nkx2.5-F	5'-gaccctcgggcggataaaaa-3'
	Nkx2.5-R	5'-acttgtagcgacggttctgg-3'
Primers for qRT-PCR in Human cells	*ACTB*-F	5'-caccattggcaatgagcggttc-3'
	ACTB-R	5'-aggtctttgcggatgtccacgt-3'
	GAPDH-F	5'-ggagcgagatccctccaaaat-3'
	GAPDH-R	5'-ggctgttgtcatacttctcatgg-3'
	UBE3D-F	5'- atcctgggagaaccgaaag-3'
	UBE3D-R	5'-cctaattttgcttgcgtctg-3'
	CPSF3-F	5'-gcacgtttacagcaagaggttgg-3'
	CPSF3-R	5'-aaggttggcagttttcccgtcc-3'
	HOXA5-F	5'-atgcgcaagctgcacataag-3'
	HOXA5-R	5'-cgggtcaggtaacggttgaa-3'
	HOXA9-F	5'-aaaaacaacccagcgaaggc-3'
	HOXA9-R	5'-accgctttttccgagtggag-3'
	HOXA10-F	5'-ggattccctgggcaattcca-3'
	HOXA10-R	5'-ctaatctctaggcgccgctc-3'
	HOXA11-F	5'-cttccggccacactgagga-3'
	HOXA11-R	5'-cgctgaagaagaactcccgtt-3'
	HOXA13-F	5'-ctctggaagtccactctgcc-3'
	HOXA13-R	5'-attcgtggcgtattcccgtt-3'
	HOXC10-F	5'-cacctcggataacgaagcga-3'
	HOXC10-R	5'-tccaattccagcgtctggtg-3'
	HOXD10-F	5'-cgtgtccagtcccgaagtg-3'
	HOXD10-R	5'-gtgcttagtgtaagggcacc-3'
	HOXD11-F	5'-cagcagcgcagttgcc-3'
	HOXD11-R	5'-tgtacacgttaaagaaaaactcgc-3'
	EMX2-F	5'-cgggatccgtccaccttcta-3'
	EMX2-R	5'-gcaaaaggaaactctcgggg-3'
	LHX2-F	5'-ctttgccattaaccacaacc-3'
	LHX2-R	5'-gtgttttcctgccgtaagag-3'
	MNX1-F	5'-gcaccagttcaagctcaacaa-3'
	MNX1-R	5'-tccatttcatccgccggttc-3'
	NKX2.5-F	5'-ccgaaaagaaagagctgtgcg-3'
	NKX2.5-R	5'-gtggacgtgagtttcagcac-3'
Primers for PCR in mouse	*Ube3d*-id-603-F	5'-ctgggtgggccagatgaaggag-3'
	Ube3d-id-603-R	5'ccttggtatgttaggaaaacttc-3'
