# Supplementary material for: Deficiency of UBE3D in mice leads to severe embryonic abnormalities and disrupts the mRNA of Homeobox genes via CPSF3
Source: Cell Death Discov. 2025 Mar 12;11:99. doi: 10.1038/s41420-025-02387-y (PMC11904178; doi:10.1038/s41420-025-02387-y)

Original Western Blots

Fig. 1d

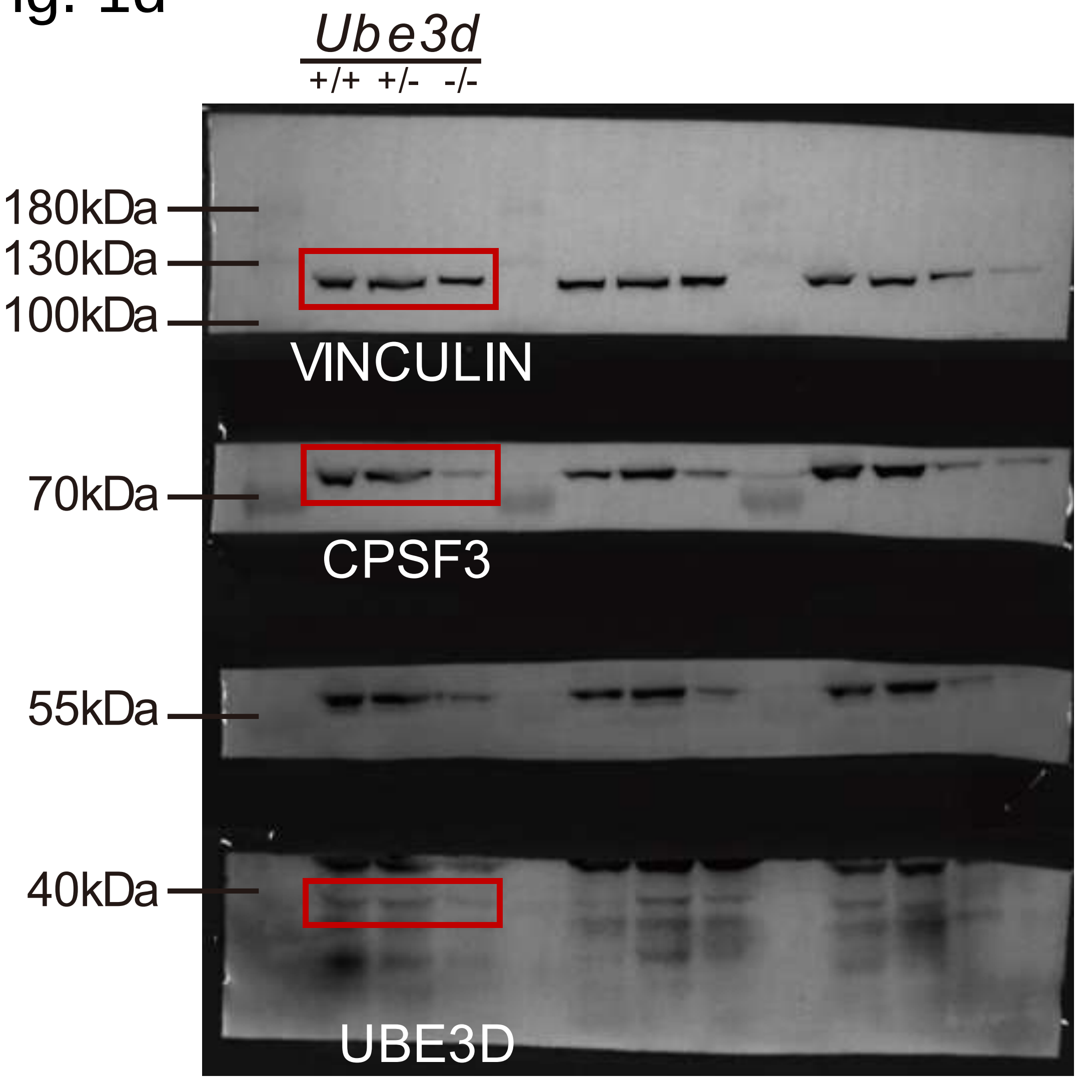

Original Western Blots

Fig. 6a

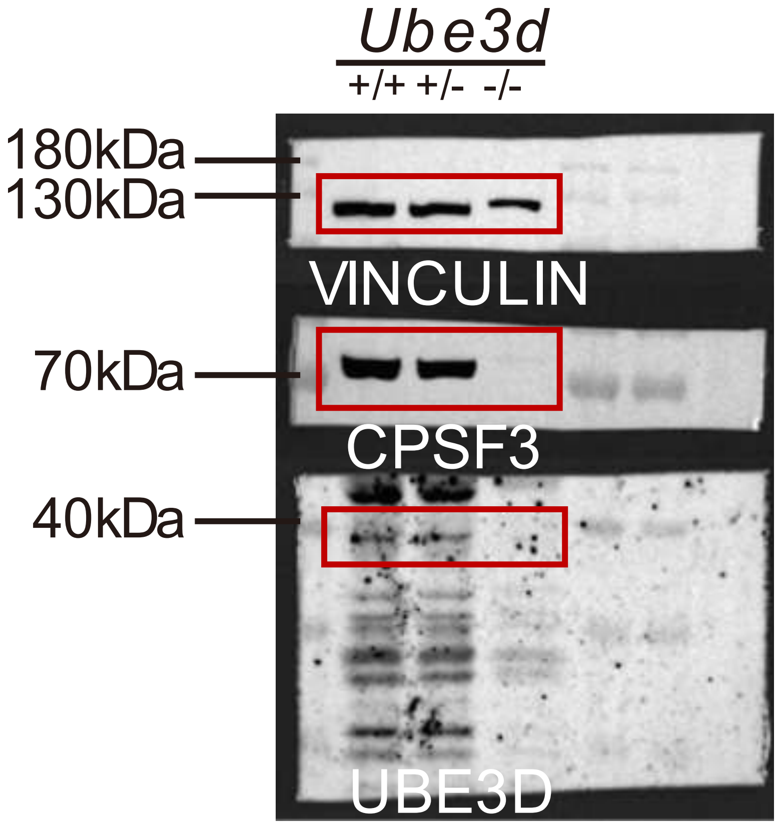

Fig. 6d

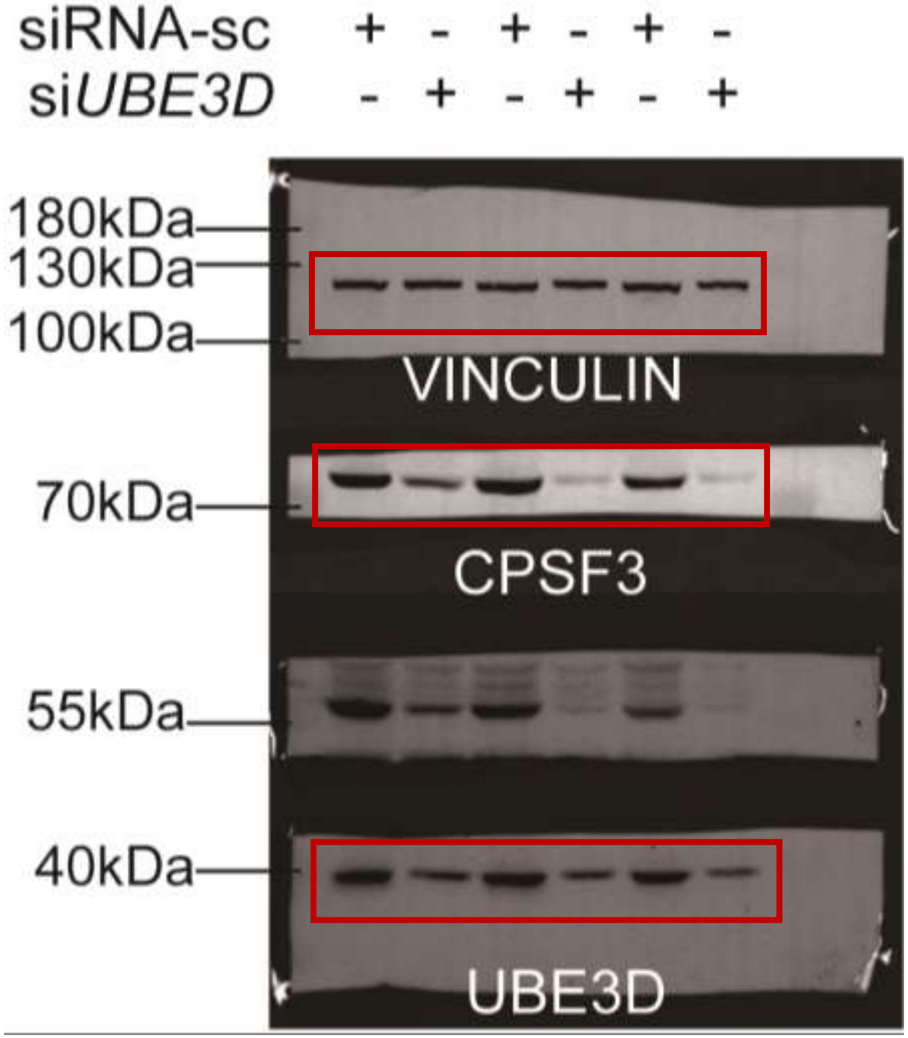

Original Western Blots

Fig. 6g

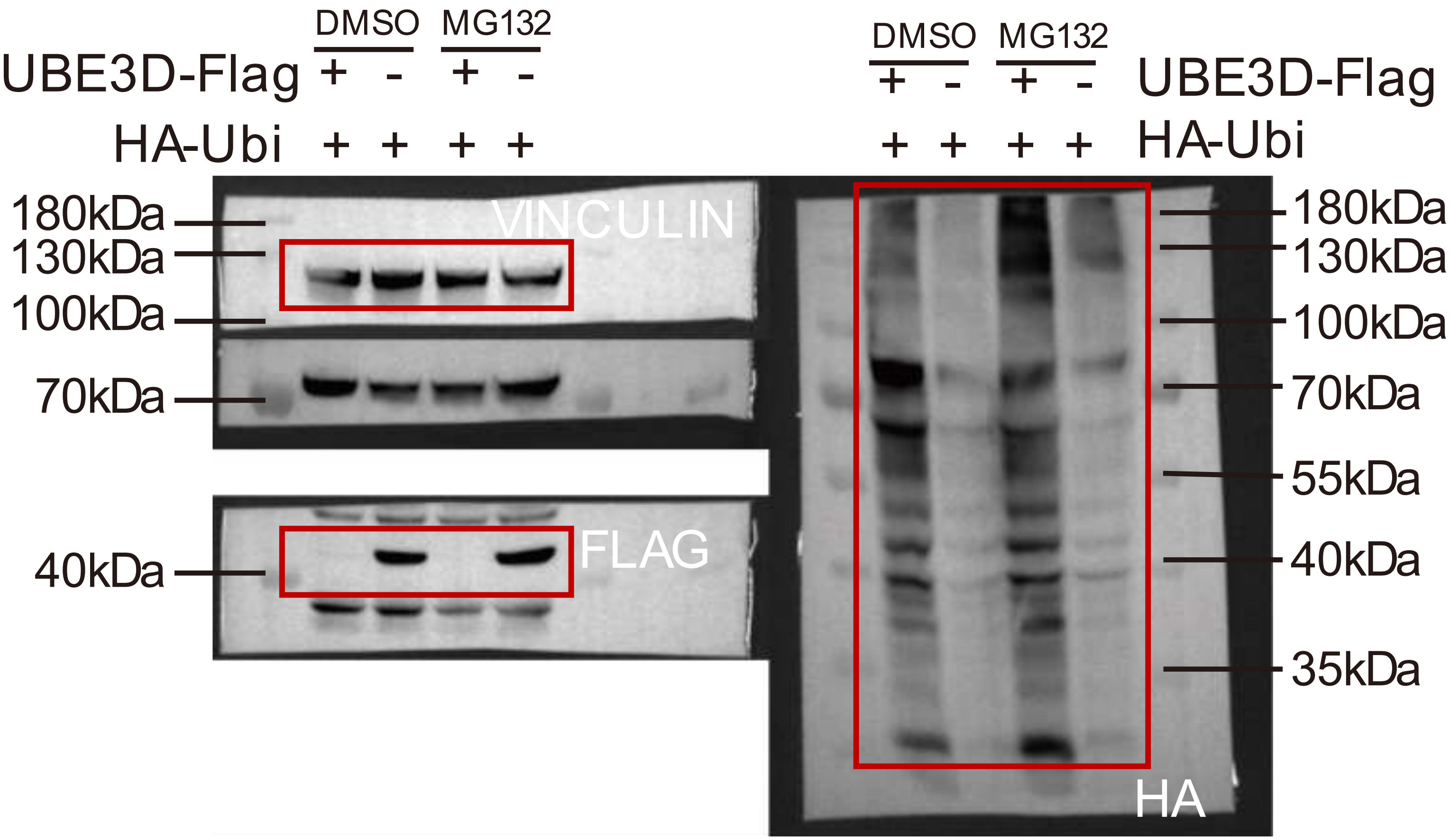

Original Western Blots

Fig. 6h

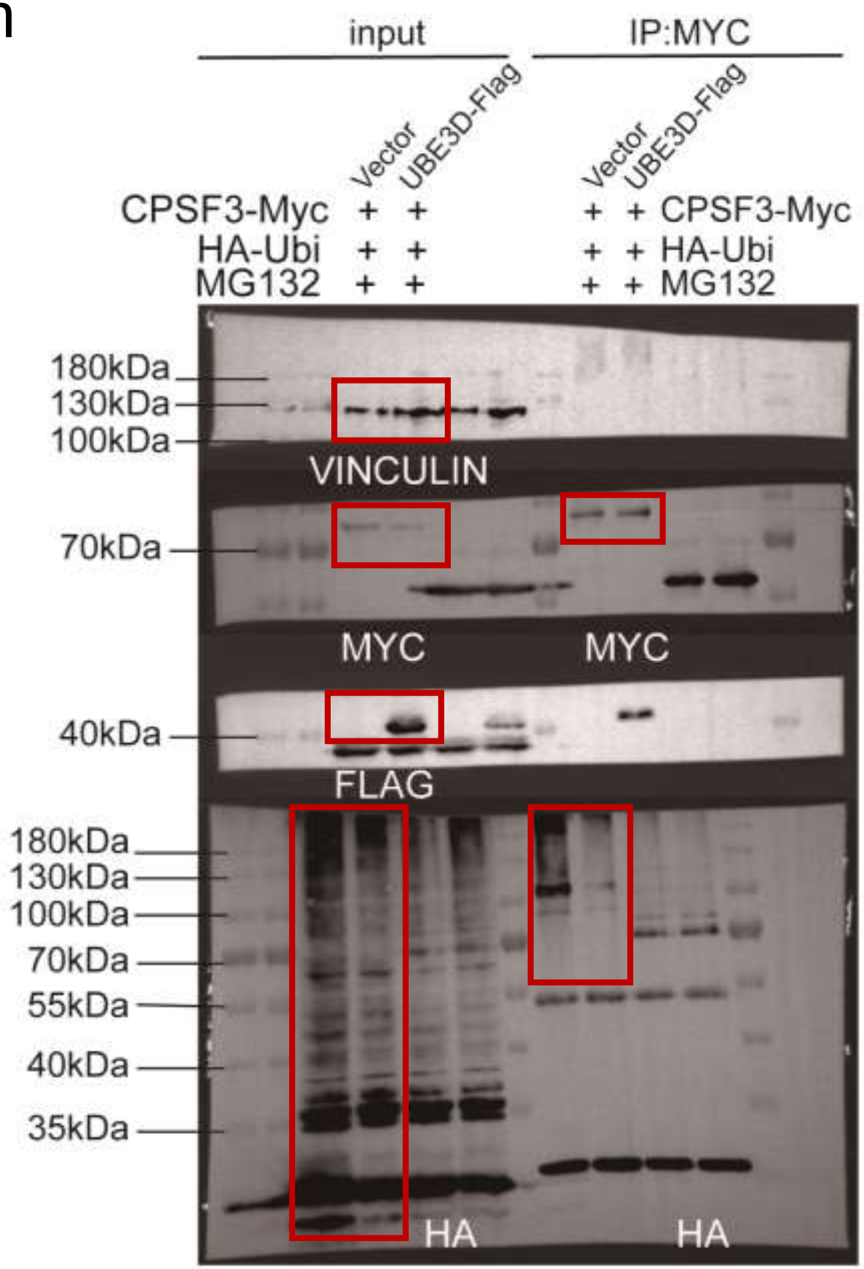

Original Western Blots

Fig. 7b

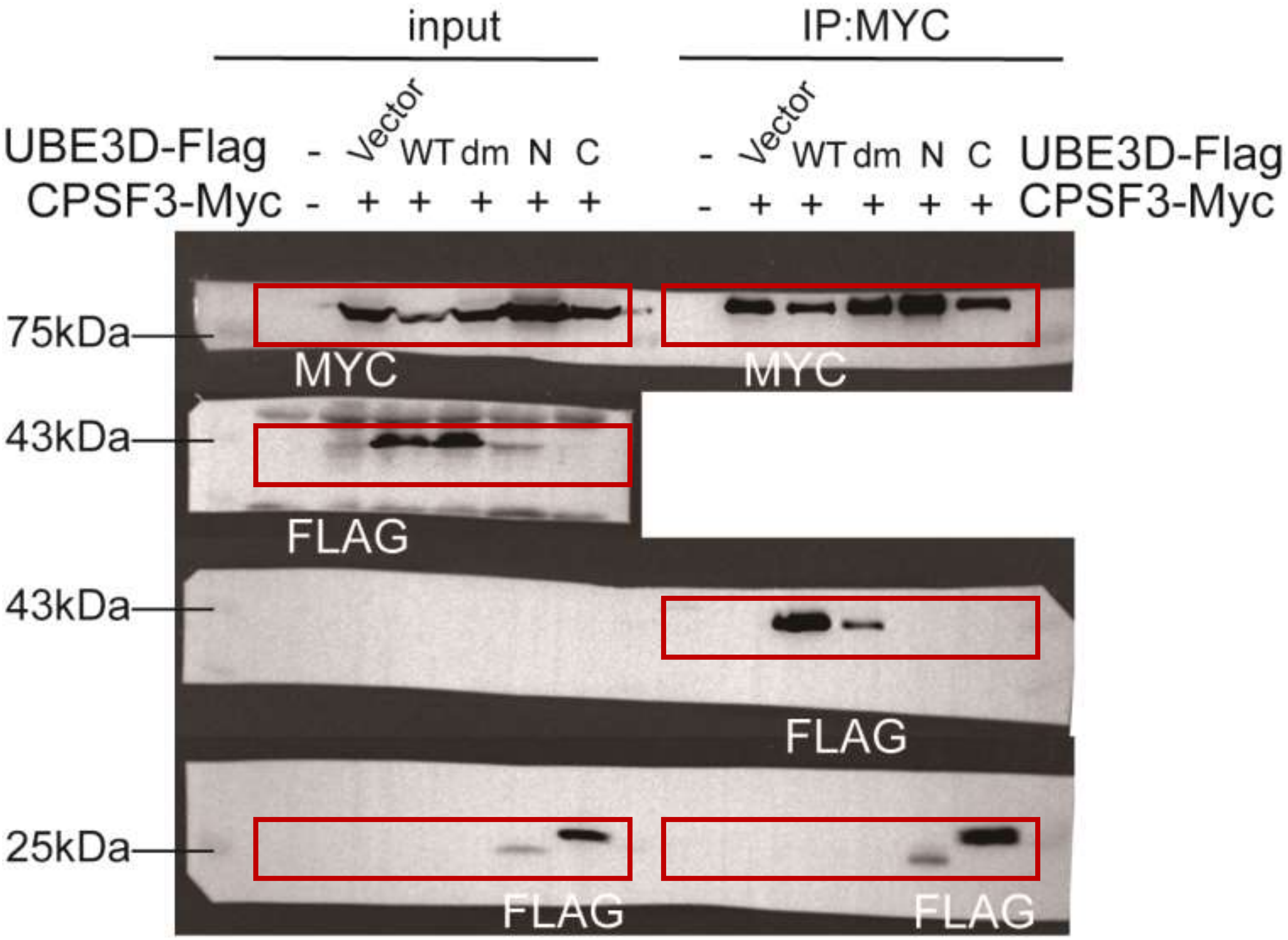

Original Western Blots

Fig. 7c

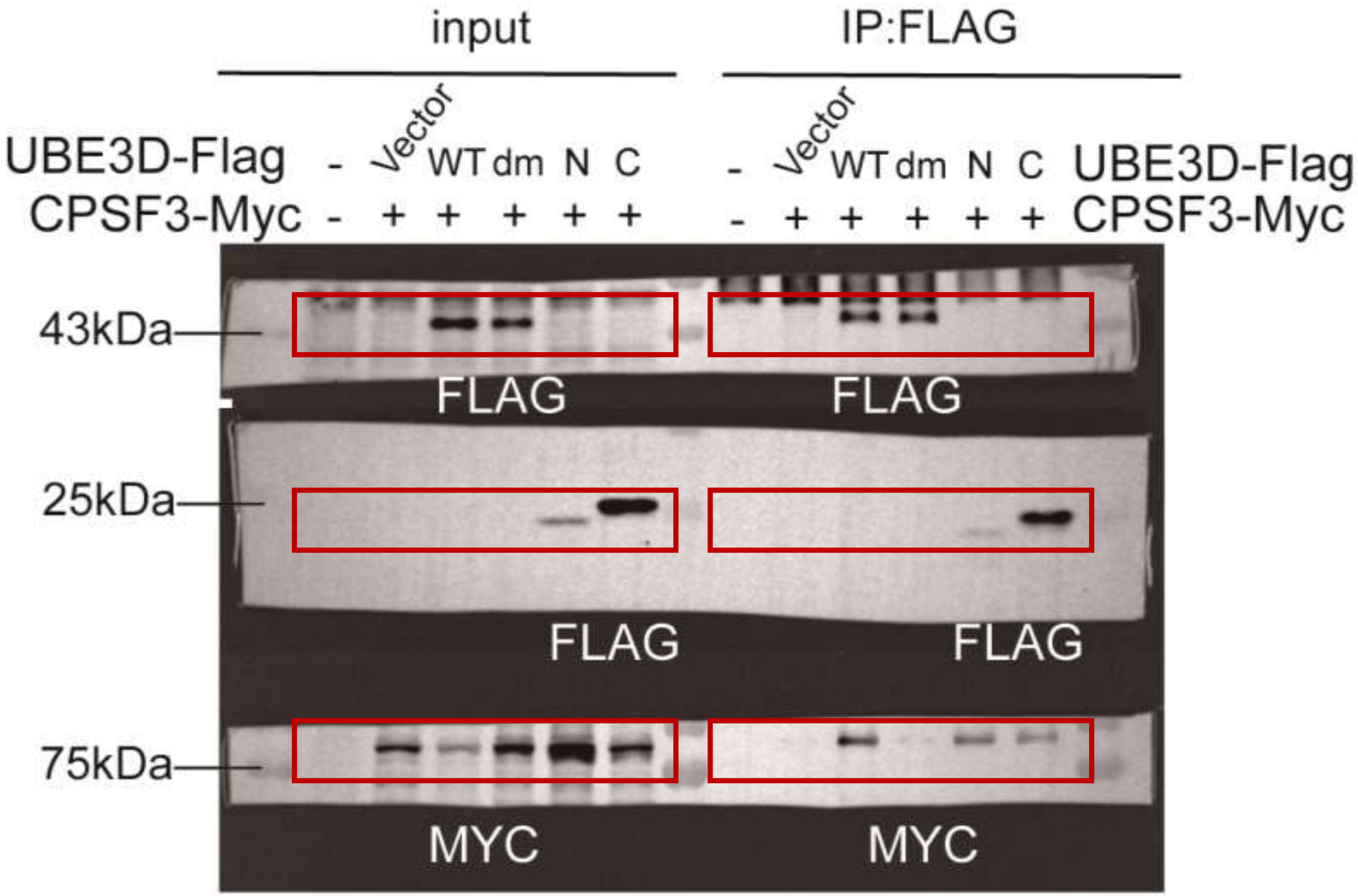

Original Western Blots

Fig. 7d

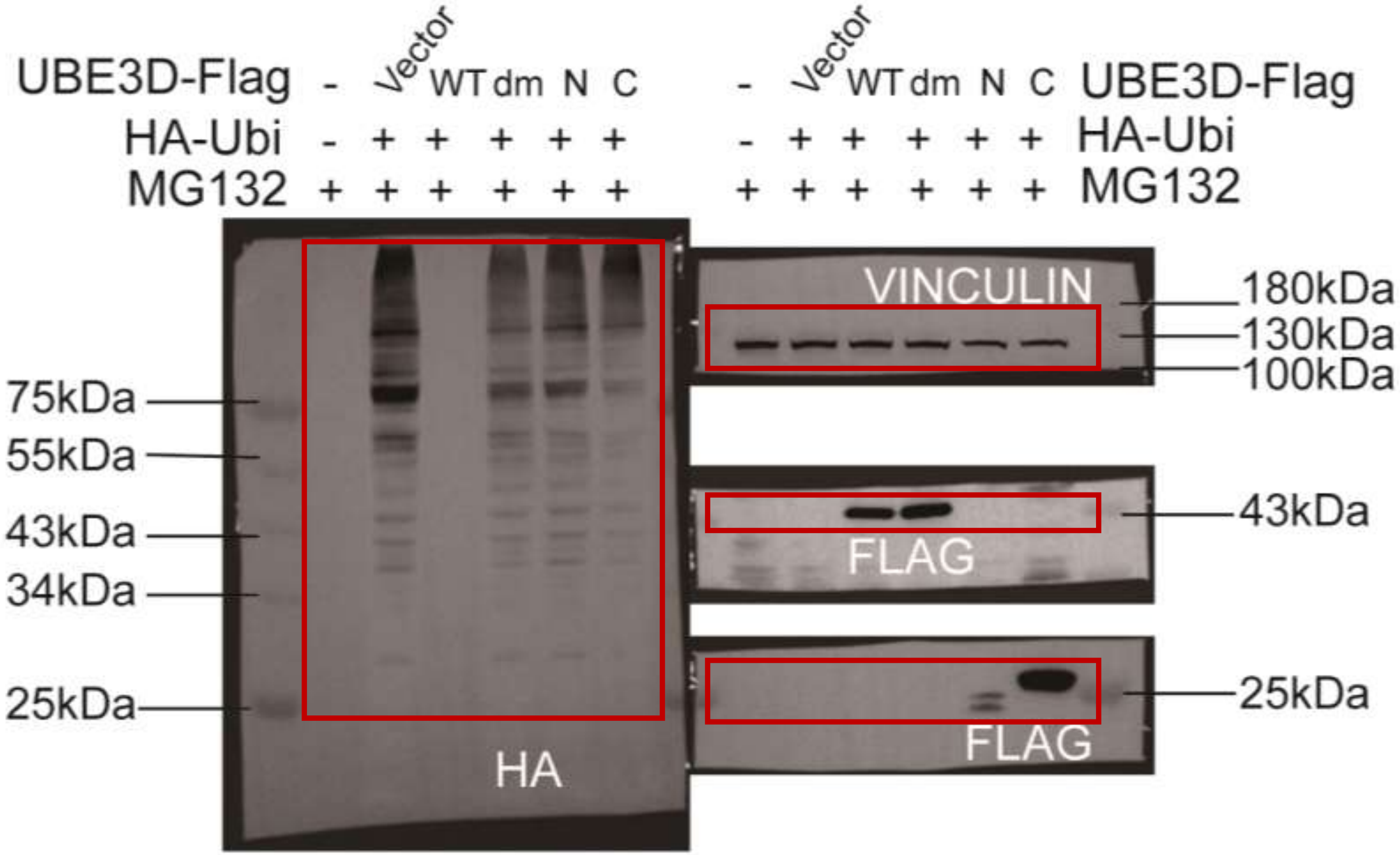

Original Western Blots

Fig. 7f

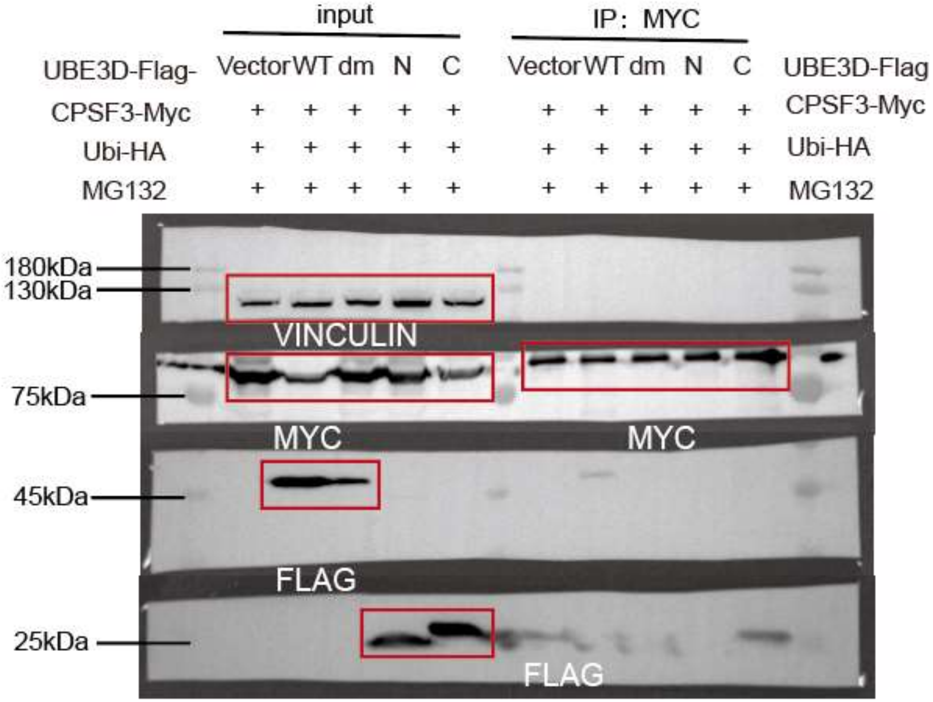

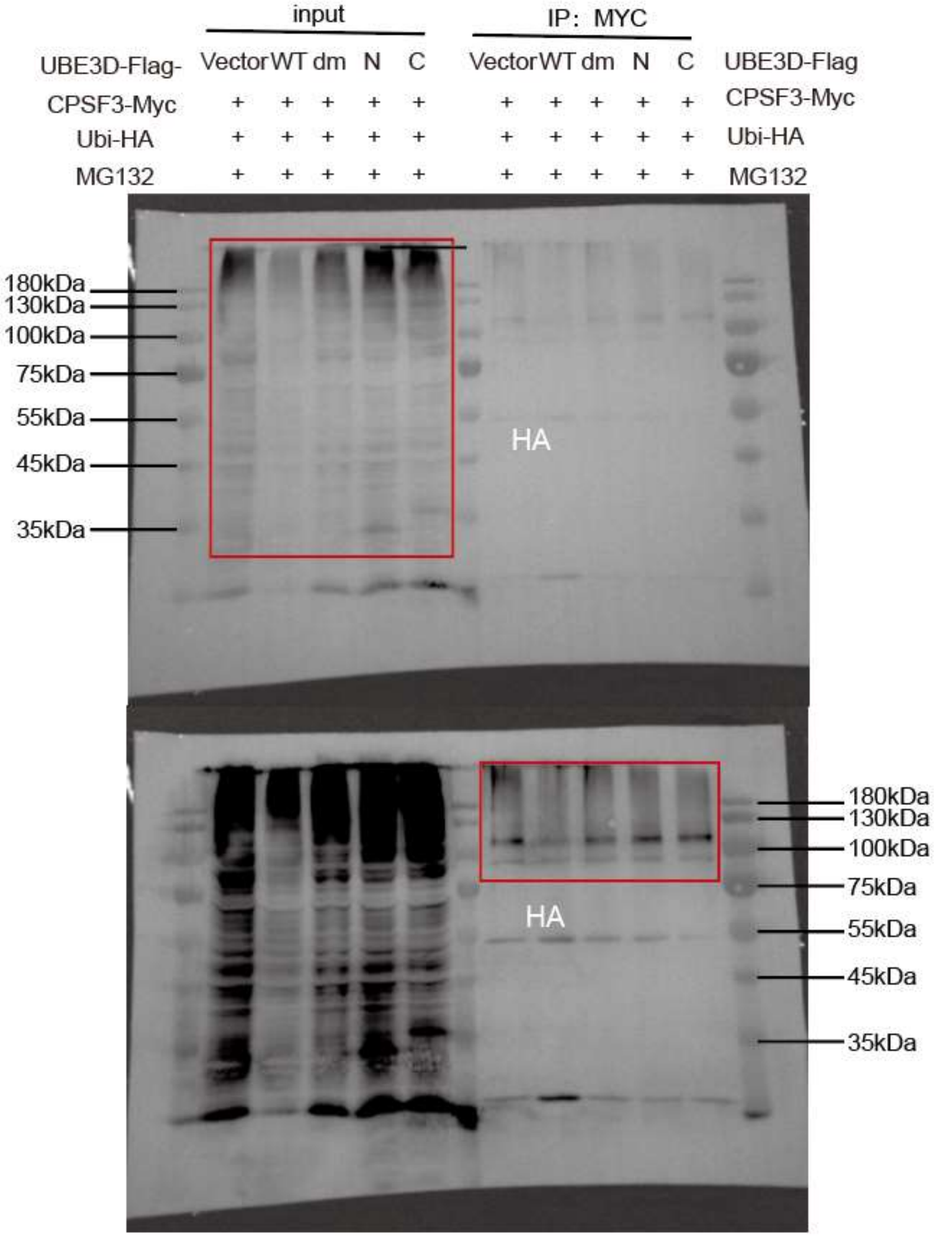

Original Western Blots

Fig. 8b

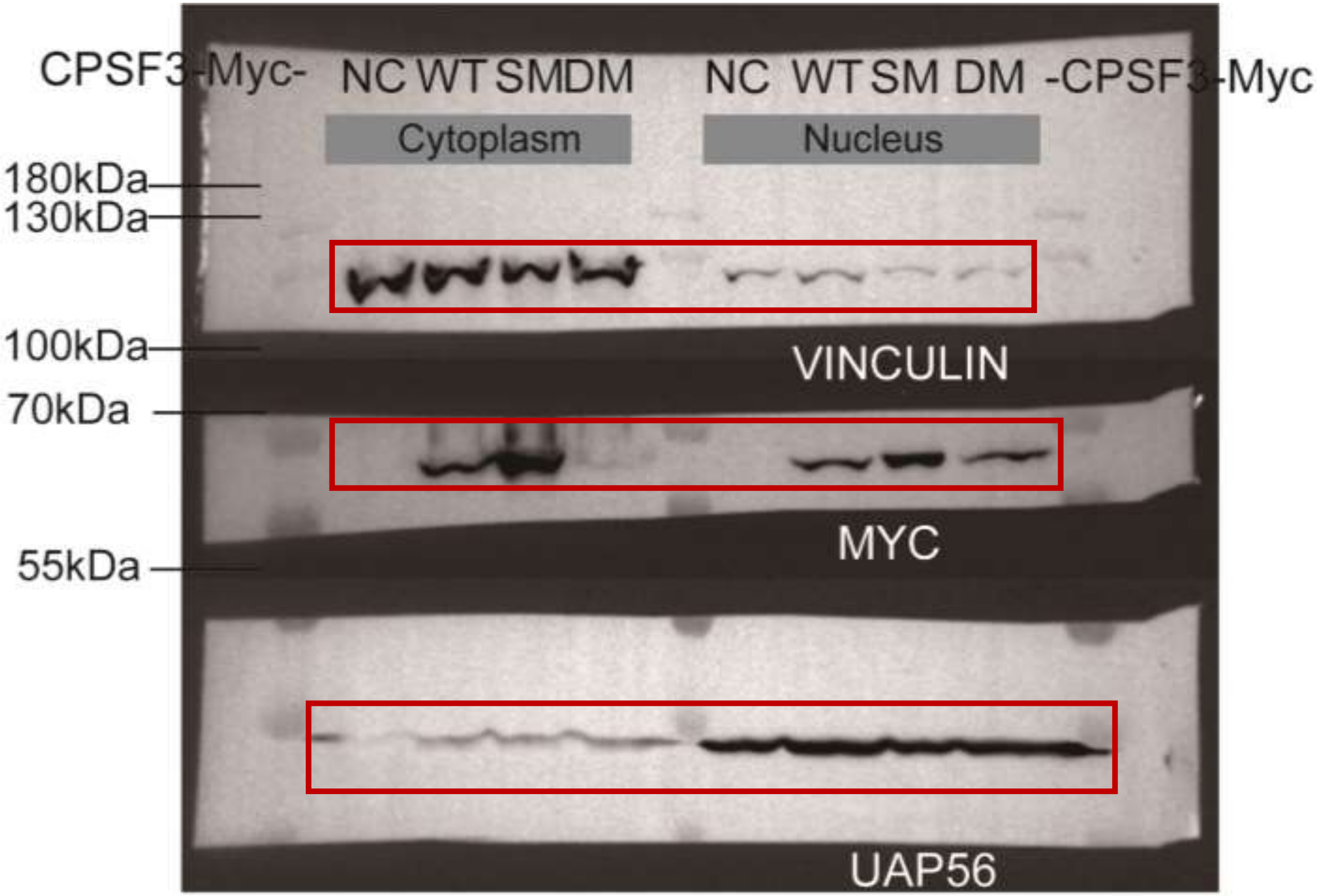

Original Western Blots

Fig. 8c

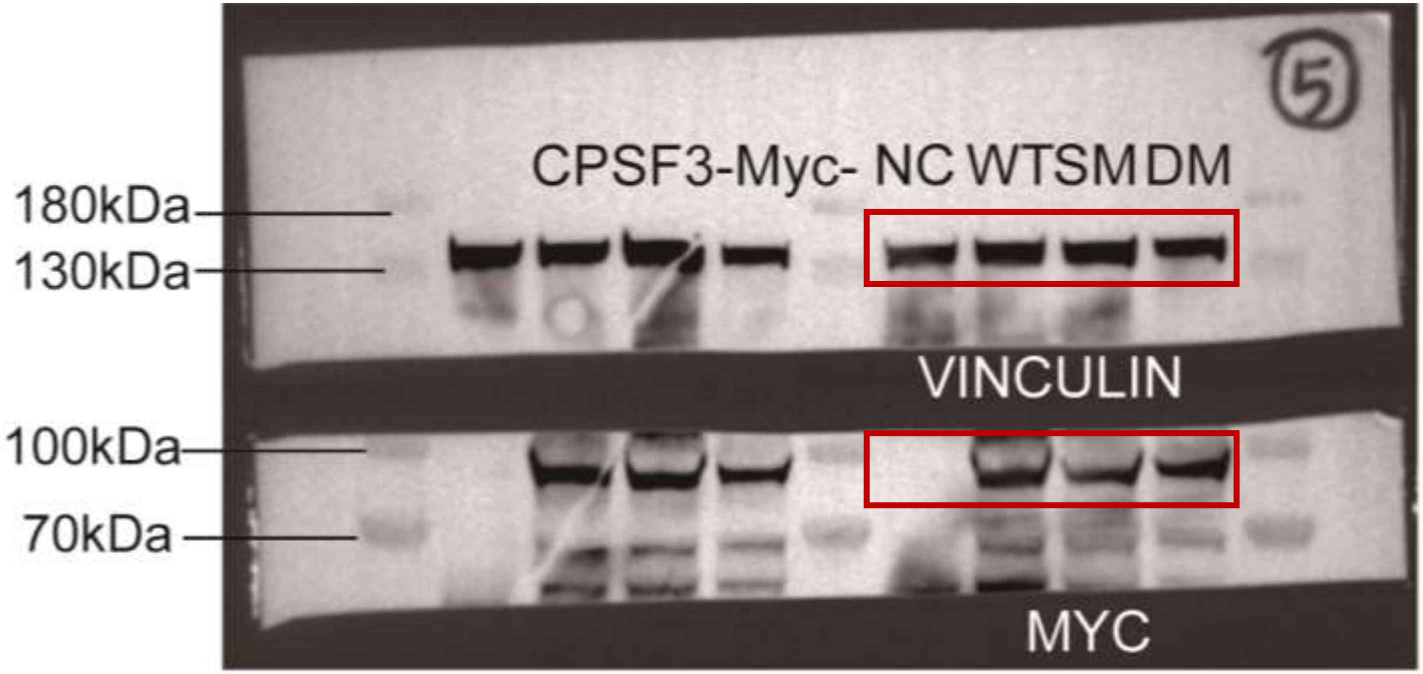

Original Western Blots

Fig. S4a

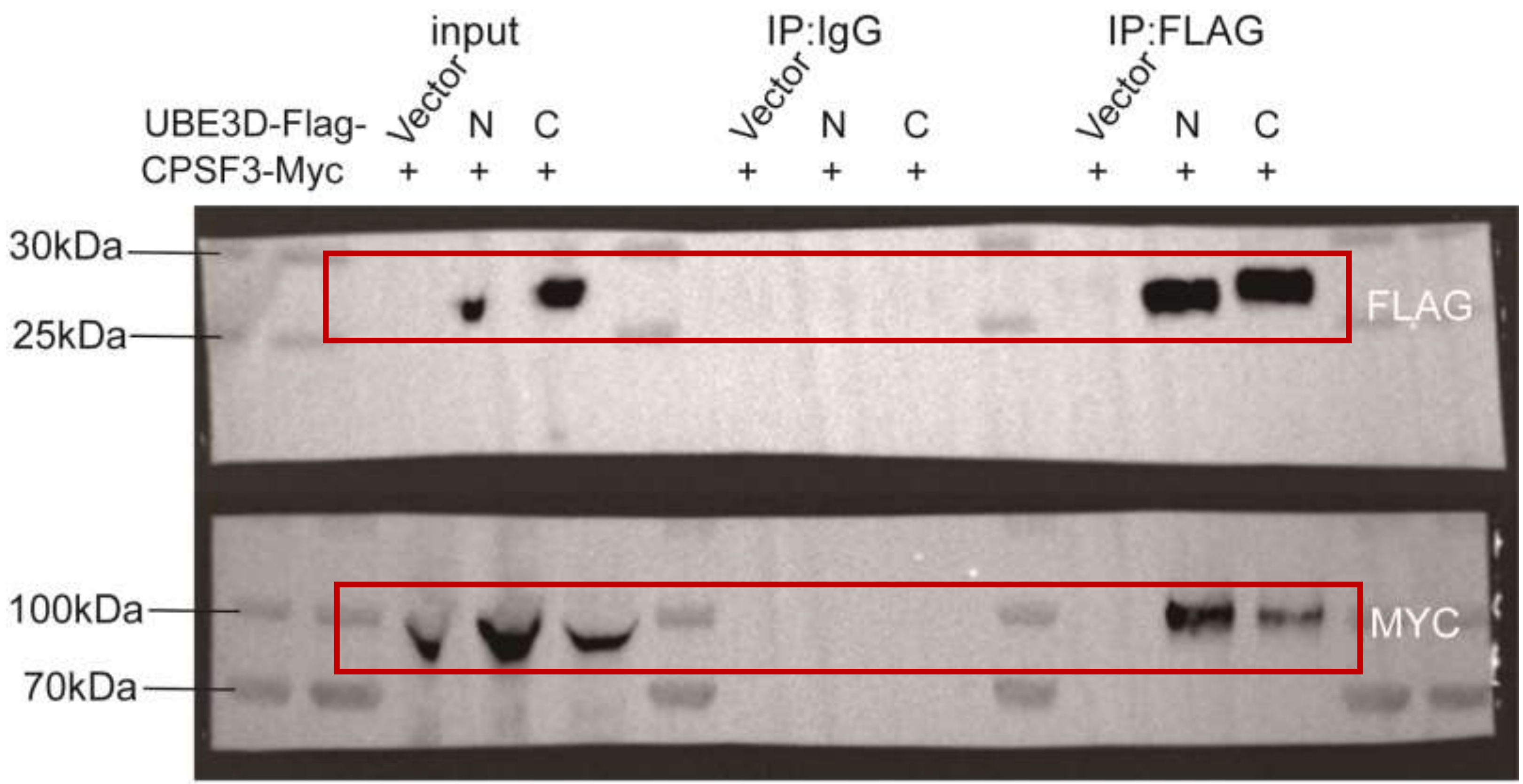

Original Western Blots

Fig. S4b

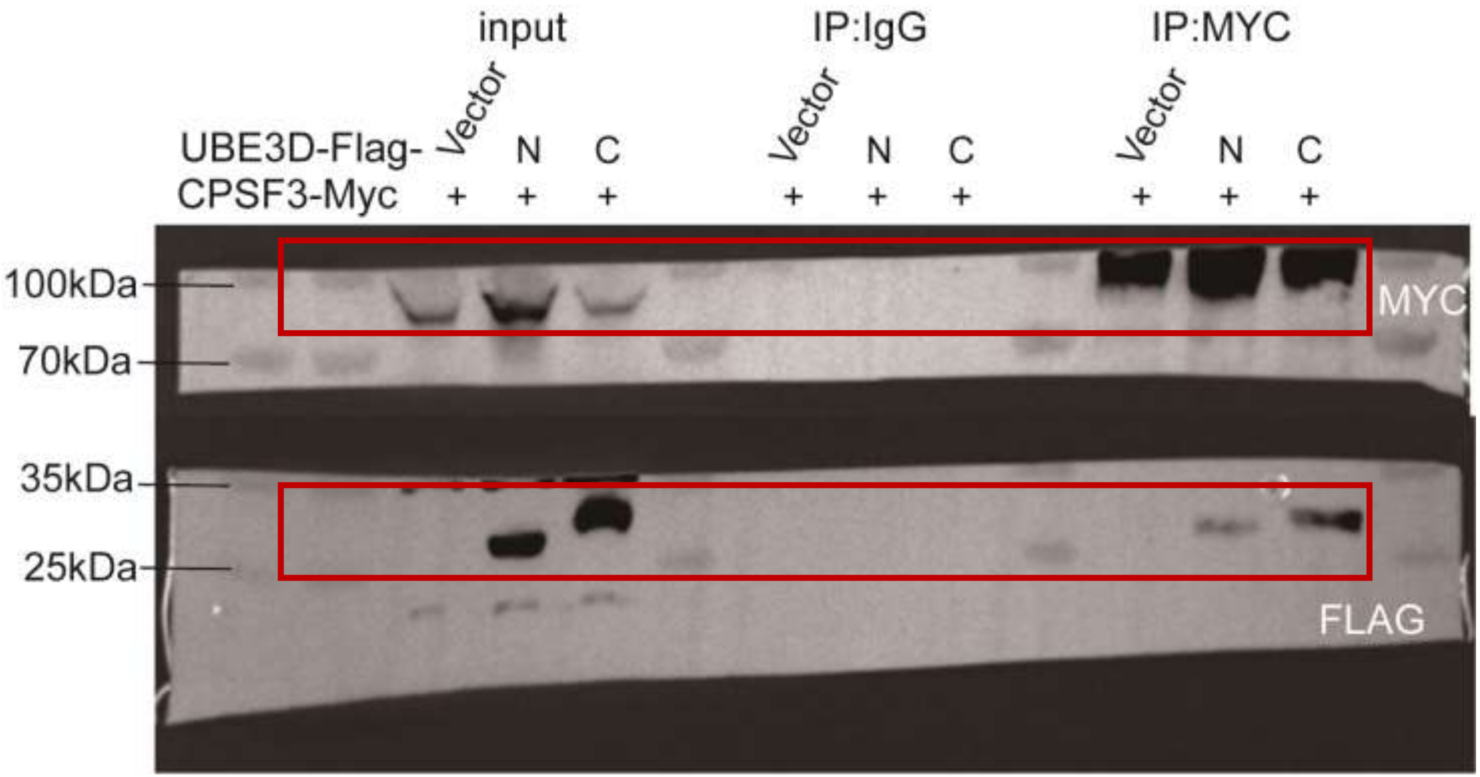

Supplement: Supplementary file 3 — Original Western blots [file 41420_2025_2387_MOESM3_ESM.pdf]
